# Functional MXene Inks for Printed Electronics: Chemistry, Rheology, and Devices

**DOI:** 10.1002/advs.77906

**Published:** 2026-10-26

**Authors:** Shahzaib Ali, Faisal Shahzad, Hamza Hussain, Muhammad Abbas, Dae‐Hyun Cho, Doyoung Byun, Hassan A. Arafat

**Affiliations:** ^1^ Department of Mechanical Engineering Sungkyunkwan University (SKKU) Suwon‐si Gyeonggi‐do Republic of Korea; ^2^ Research and Innovation Center for Graphene and 2D Materials (RIC2D) Khalifa University Abu Dhabi United Arab Emirates; ^3^ Department of Mechatronics Engineering Gyeongsang National University Jinju Gyeongnam Republic of Korea; ^4^ Department of Energy System Engineering Gyeongsang National University Jinju Gyeongnam Republic of Korea; ^5^ Department of Display Engineering Sungkyunkwan University (SKKU) Suwon Republic of Korea

**Keywords:** Additive manufacturing, Functionalization, MXene inks, MXetronics, Printing

## Abstract

MXene‐based functional inks have emerged as a promising platform for printed electronics by combining high electrical conductivity, tunable surface chemistry, and solution processability. This review establishes a unified framework linking MXene synthesis, surface engineering, ink rheology, printing behavior, and device performance across diverse additive manufacturing technologies. Particular emphasis is placed on how chemical functionalization, composite design, and interfacial engineering influence colloidal stability, printability, and functionality. By elucidating structure–ink–process–property relationships, the review reveals how MXene chemistry and rheology govern printed microstructure and performance in sensors, energy‐storage devices, and electromagnetic interference shielding systems. Despite rapid progress, widespread implementation remains constrained by three interconnected challenges: ensuring long‐term chemical and colloidal stability, achieving printability without sacrificing electrical transport, and establishing scalable, reproducible manufacturing routes. Future advances will depend on oxidation‐resistant formulations, manufacturing‐aware rheological design, sustainable processing strategies, and standardized evaluation protocols. These developments provide a roadmap for translating MXene inks from laboratory‐scale demonstrations to reliable, high‐performance flexible and multifunctional electronic systems.

## Graphical abstract

Functional MXene inks are emerging as building blocks for MXetronics, connecting MXene synthesis, surface functionalization, ink formulation, and advanced printing with device‐level applications. This review maps how composition, stability, rheology, and processability govern printed structures and enable high‐performance sensors, energy‐storage devices, electromagnetic shielding, heaters, and flexible electronics.

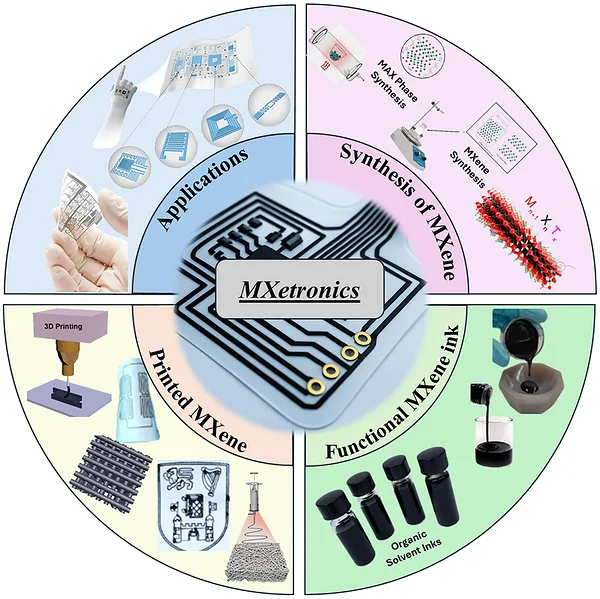

## 1 Introduction

The growing demand for flexible, miniaturized electronics has accelerated interest in additive manufacturing routes that can deliver scalable, high‐performance devices from two‐dimensional (2D) materials [1, 2, 3, 4, 5, 6, 7, 8]. Among the 2D materials, MXenes (2D transition metal carbides, nitrides, and carbonitrides) derived from MAX phases have gained attention for their metallic conductivity, hydrophilicity, solution processability, and tunable surface terminations [9, 10, 11, 12, 13, 14, 15, 16, 17, 18]. These properties uniquely position MXenes for the development of functional inks compatible with advanced printing techniques [1, 7, 19, 20, 21, 22, 23]. Recent studies have further established MXenes as versatile building blocks for printed and flexible electronics by demonstrating their utility across sensing, energy‐storage, and bioelectronic platforms [24]. For example, MXene‐based sensing systems have shown high sensitivity toward chemical, biological, and mechanical stimuli, while MXene electrodes and hybrid architectures have been widely explored for supercapacitors, micro‐energy devices, and multifunctional wearable systems. These studies collectively highlight that the device performance of MXene‐based printed electronics is not determined by intrinsic conductivity alone, but by the combined control of flake quality, surface terminations, colloidal stability, rheological behavior, interfacial adhesion, and post‐printing microstructure [25]. Therefore, a review that connects MXene chemistry, ink formulation, printability, and device‐level performance is necessary to guide the transition from isolated demonstrations toward reproducible printed MXetronics systems [26]. Translating colloidal MXenes into printable architectures requires coupling ink formulation with rheological control and surface chemistry so that printing windows and device performance are met simultaneously in diverse electronic applications, including sensors, energy storage, electromagnetic interference (EMI) shielding, and beyond [14, 27, 28, 29, 30, 31, 32, 33, 34]. The solution processability of MXenes has spurred innovative approaches for formulating functional inks, thereby enabling a myriad of printing techniques such as inkjet, screen, blade, spray, direct ink writing (DIW), and electrohydrodynamic (EHD) printing [1, 13, 19, 35, 36, 37]. Key rheological descriptors like viscosity, surface tension, and particle size distribution must be tuned to enable stable droplet/filament formation, suppress nozzle clogging, and achieve high‐resolution patterning [5, 19, 38, 39, 40, 41]. In particular, the inverse Ohnesorge number (*Z* = 1/Oh) can be modulated through MXene concentration and dispersion quality, providing a convenient printability metric for drop‐on‐demand and related processes [42, 43, 44, 45]. Such control is imperative for maintaining the performance and reproducibility of printed microelectronic architectures [46].

MXenes are traditionally produced via two broad routes: (i) top–down etching of MAX and non‐MAX precursors, (ii) bottom–up deposition routes such as chemical vapor deposition (CVD) and salt‐templated growth [11, 13, 47, 48, 49, 50, 51, 52, 53, 54]. However, pristine MXenes are hampered by rapid oxidative degradation, restacking‐driven loss of surface area, inadequate mechanical flexibility, and poor non‐aqueous dispersibility that leads to aggregation and sedimentation [55, 56, 57, 58, 59]. These deficiencies restrict their utility across a variety of emerging technologies [48, 50, 60, 61, 62]. Recent breakthroughs have addressed these challenges on two complementary fronts: optimizing the rheological properties of printable inks and tailoring the surface chemistry of individual nanosheets [11, 28, 63, 64, 65, 66]. In particular, surface functionalization has become a critical step in MXene processing [38, 67, 68, 69]. In this regard, organic ligands, particularly the catechol‐based moieties, which can anchor strongly to the MXene surface, can suppress oxidation, prevent restacking, and stabilize the dispersions in both aqueous and non‐aqueous media without sacrificing intrinsic properties [20, 70, 71, 72, 73, 74]. By simultaneously tuning viscosity and eliminating the need for sacrificial additives, these ligand shells enable direct formulation of binder‐free, low‐temperature inks compatible with flexible substrates [2, 75, 76, 77]. Such advances have propelled MXene inks into high‐value printed‐electronics applications, including micro‐supercapacitors, micro‐batteries, thin‐film transistors (TFTs), integrated sensor arrays, EMI shielding, and energy harvesting, underscoring the indispensable role of surface chemistry in translating MXenes from laboratory curiosity to practical device platforms [12, 28, 55, 64, 78, 79, 80, 81].

Moreover, the interplay between MXene ink formulation and printing methodologies has catalyzed the emergence of novel microelectronic components characterized by intricate architecture and multifunctionality [63, 82, 83, 84]. The integration of MXene‐based electrodes with complementary devices, such as nanogenerators, solar cells, and flexible displays, underscores the potential of these materials to revolutionize the field of wearable and portable electronics [85, 86, 87]. Despite significant progress, from the early adoption of DIW to more sophisticated approaches such as EHD, electrospinning [25], and other methods highlighted in Figure 1, several critical challenges persist. These challenges include the precise tuning of ink rheology to match specific printing techniques, the mitigation of interfacial issues between printed layers and substrates, and the stabilization of MXene structures against environmental degradation [88].

**FIGURE 1:**
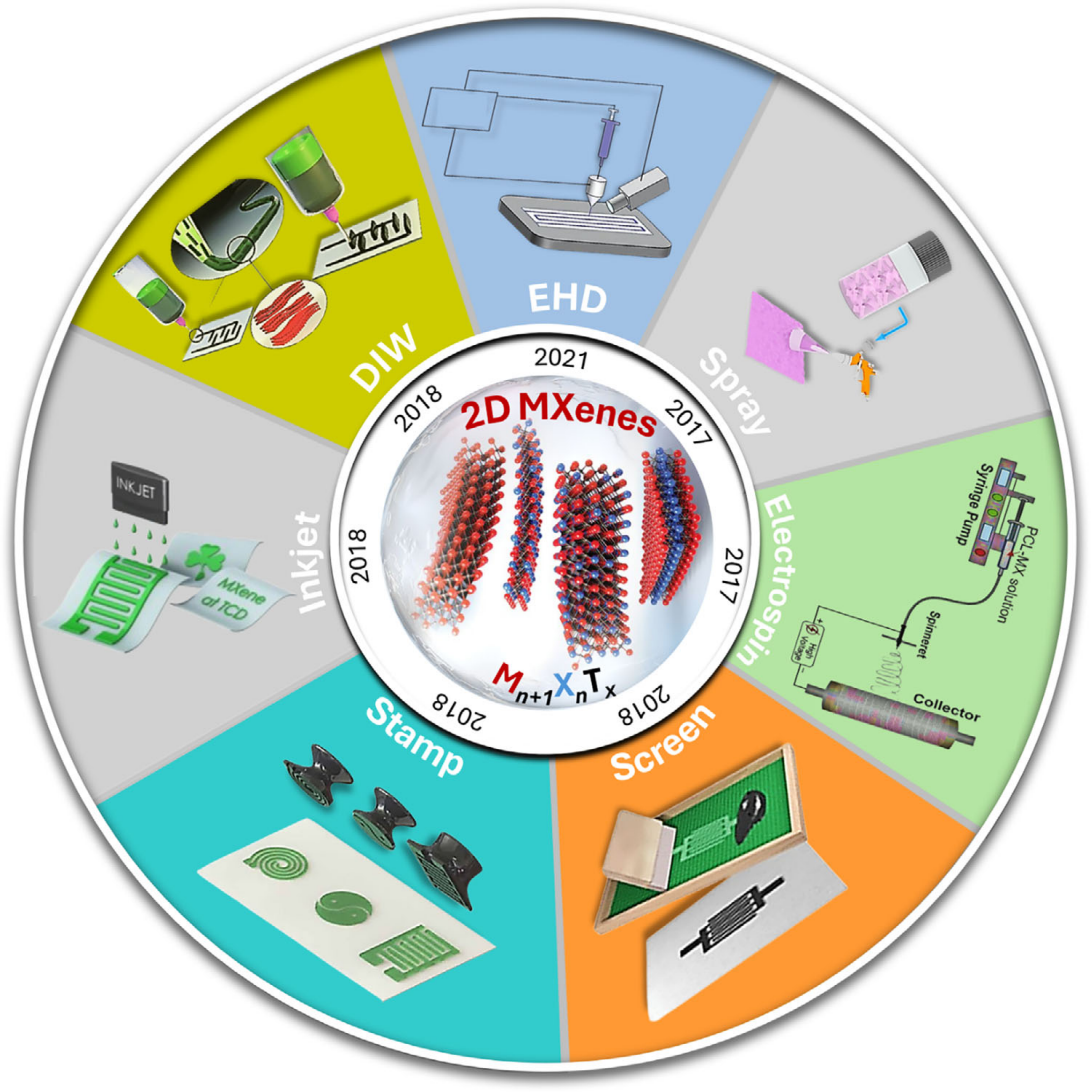
Overview of MXene printing techniques. Inkjet printing, Reproduced with permission [22]. Copyright 2019, Nature. DIW, reproduced with permission [89]. Copyright 2020, ACS. EHD printing, reproduced with permission [90]. Copyright 2025, Elsevier. Spray coating, reproduced with permission [91]. Copyright 2020, ACS. Stamp printing, reproduced with permission [92]. Copyright 2018, Wiley. Screen printing, reproduced with permission [46].Copyright 2018, Wiley. Electrospinning, reproduced with permission [93]. Copyright 2020, Elsevier.

Previous reviews have summarized MXene synthesis, ink formulation, printing methods, device applications, and the challenges of large‐area and high‐resolution integration. Building on these studies, the present Review provides an updated and integrated assessment of functional MXene inks across the full pathway from synthesis and surface functionalization to colloidal behavior, quantitative rheology, printing compatibility, printed microstructure, and device performance, as summarized in Scheme 1. Particular emphasis is placed on dispersion stability, the conductivity–printability trade‐off, and applications in sensors, energy storage, EMI shielding, wireless electronics, nanogenerators, TFTs, and memory devices. The Review further examines manufacturing‐oriented challenges, including ink–substrate adhesion, multi‐nozzle EHD scalability, long‐term reliability, sustainability, commercialization, and the technology‐readiness levels of printed MXene technologies.

**SCHEME 1:**
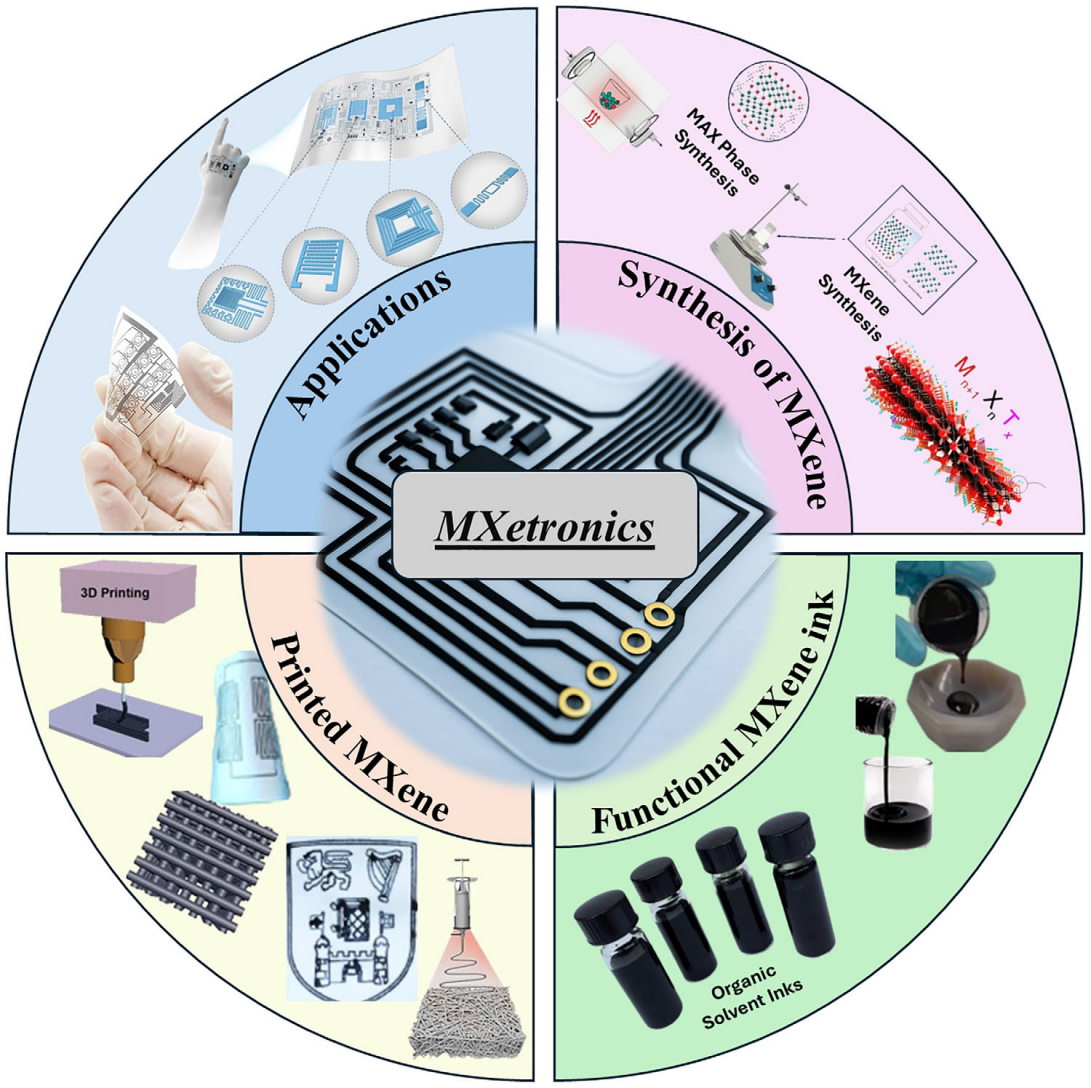
Schematic illustration of functional MXene inks for printed MXetronics. Applications, Reproduced with permission [94]. Copyright 2022, Nature. Synthesis of MXenes, Reproduced with permission [95]. Copyright 2022, Nature. Functional MXene ink, Reproduced with permission [22, 96]. Copyright 2019, Nature. Printed MXene, Reproduced with permission [97]. Copyright 2019, Wiley.

To compare process capabilities, six representative techniques, namely DIW, blade coating, screen printing, spray coating, inkjet printing, and EHD printing, are evaluated across resolution, print speed, material compatibility, layer‐thickness control, scalability, and viscosity tolerance (Figure 2). DIW accommodates high‐viscosity inks but offers moderate resolution; blade and spray coating provide rapid large‐area deposition with limited patterning precision; screen printing combines scalability with broad viscosity tolerance; inkjet printing enables precise deposition but requires low‐viscosity, well‐dispersed inks; and EHD printing provides exceptionally fine resolution, although its scale‐up is limited by throughput and multi‐nozzle synchronization challenges. These comparisons highlight the need to match MXene ink chemistry and rheology with process‐specific requirements to enable reliable and scalable printed devices.

**FIGURE 2:**
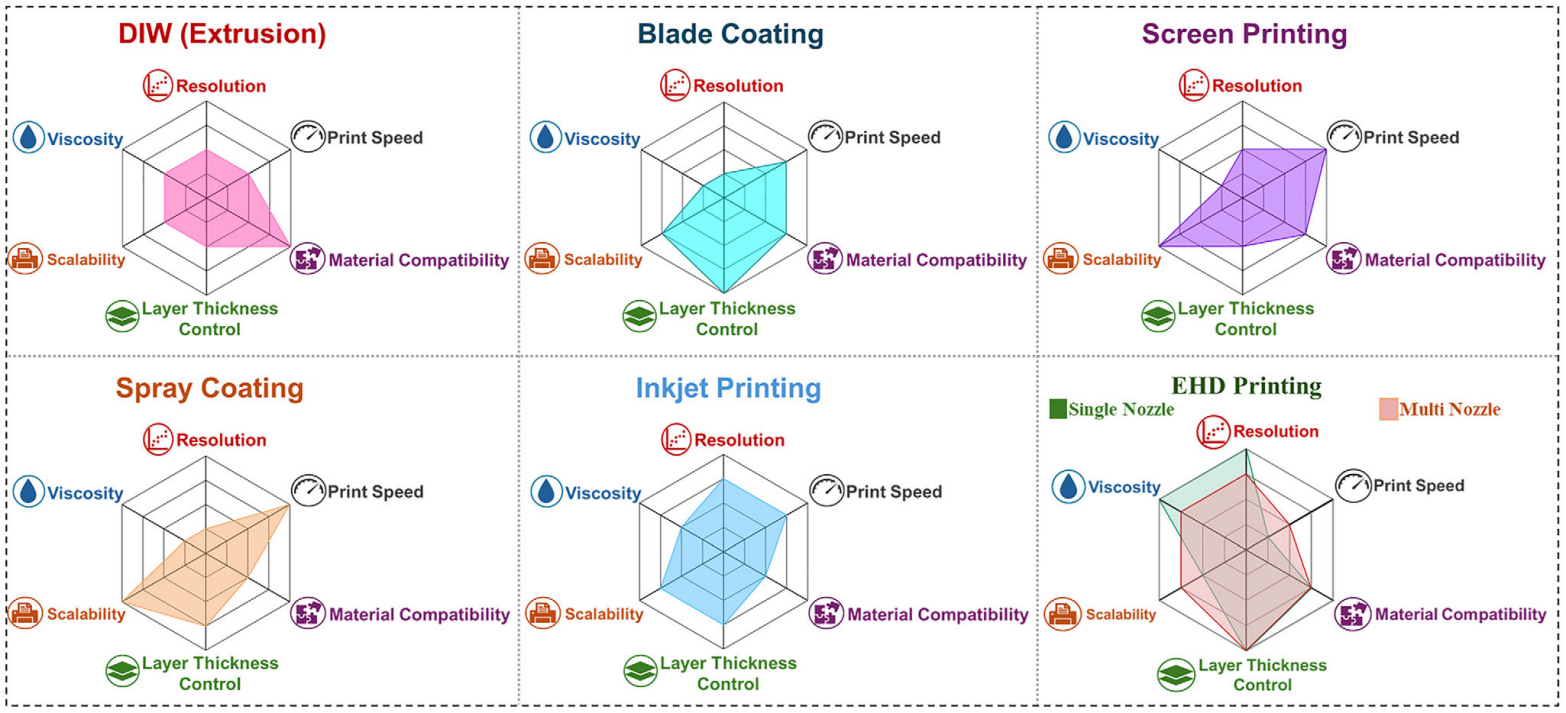
Spider charts comparing six printing techniques including blade coating (cyan), direct‐ink‐writing/extrusion (magenta), screen printing (purple), spray coating (orange), EHD jet printing (Single nozzle: green, multi‐nozzle: red), and inkjet printing (blue) across six key performance metrics: resolution, print speed, material compatibility, layer‐thickness control, scalability, and viscosity. Higher values on each axis denote stronger performance at that attribute, highlighting the trade‐offs inherent to each method. Note: Scores represent a semi‐quantitative author assessment on an ordinal scale from 1 (relatively limited) to 5 (comparatively strong), informed by the literature and reported process characteristics. For resolution, a higher score denotes finer achievable features. The scores are intended to show relative trends and are not derived from statistical normalization, weighting, or direct head‐to‐head experimental comparisons.

This scalability advantage of multi‐nozzle EHD printing, however, should be interpreted as an engineering opportunity rather than a solved manufacturing route. Multi‐nozzle EHD printing has been proposed to overcome the intrinsically low throughput of single‐nozzle EHD systems by increasing the number of simultaneously operating nozzles; for example, multi‐nozzle EHD heads have demonstrated reduced printing time compared with single‐nozzle operation while maintaining pattern fidelity. Nevertheless, parallel EHD operation introduces synchronization and uniformity challenges that are fundamentally linked to electrostatic jet formation. Each nozzle must maintain a stable Taylor cone or cone‐jet regime under comparable voltage, flow rate, nozzle–substrate gap, hydraulic resistance, and charge‐relaxation conditions. In multi‐nozzle arrays, small differences in meniscus geometry, local flow resistance, ink concentration, solvent evaporation, or partial nozzle clogging can lead to asynchronous jet initiation, unequal deposition rates, nonuniform line widths, variable film thickness, and spatially heterogeneous sheet resistance. More critically, neighboring nozzles can interact through their electric fields, producing electrostatic cross‐talk that distorts the field distribution around each meniscus, weakens or deflects the emitted jets, and reduces accuracy. For MXene inks, these issues are further amplified by flake‐size distribution, sedimentation, oxidation, and concentration‐dependent viscosity and conductivity. Therefore, scalable EHD printing of MXene electronics requires not only increasing nozzle number, but also optimized nozzle spacing, flow‐resistance balancing, independent or segmented voltage control, integrated or ring‐shaped counter electrodes, electrostatic shielding, substrate charge management, real‐time jet‐current monitoring, machine‐vision feedback, and closed‐loop calibration to maintain synchronized jetting and array‐level device uniformity.

## 2 Synthesis and Functionalization of MXenes for Ink Formulation

From an ink‐engineering perspective, the environmental merits of an MXene synthesis route must be considered together with its influence on nanosheet quality, surface chemistry, delamination efficiency, and colloidal stability. Conventional HF and LiF/HCl routes typically produce hydrophilic Ti_3_C_2_T_*x*_ nanosheets with mixed ─F, ─OH, and ─O terminations that disperse readily in aqueous media. However, excessive etching or sonication can reduce lateral flake dimensions and introduce oxidation‐prone defects. In contrast, Lewis‐acid molten‐salt routes avoid the direct use of HF and can produce MXenes with more uniform surface terminations, including Cl‐terminated surfaces, potentially improving chemical and thermal stability. Nevertheless, these routes generally require high‐temperature processing, residual‐salt removal, and additional intercalation or delamination before stable colloidal inks can be obtained. Their suitability therefore remains printing‐process dependent: large, minimally defective flakes are preferred for conductive films, screen printing, and DIW, whereas inkjet and EHD printing require narrower flake‐size distributions to ensure nozzle reliability and stable jetting. A comparative summary of the major synthesis routes and their printing compatibility is provided in Table 1.

**TABLE 1:** Comparison of MXene synthesis routes and their implications for ink formulation and printing‐process compatibility.

Route	Representative chemistry	Ink‐relevant advantages	Ink‐relevant liabilities	Printing implications	Typical compatibility with printing processes^a^ [85, 98]	Refs
**HF direct etching**	Direct selective removal of the A‐layer using concentrated HF	Historically established route; generally high electrical conductivity; extensive precedent for Ti_3_C_2_T_*x*_ ink preparation	High handling hazard; fluoride‐rich waste; sensitivity to over‐etching, defect formation, mixed terminations, and oxidation	Provides a widely used baseline for printable MXene inks, although flake‐size control and oxidation management are required	Inkjet: moderate; EHD: moderate to high. Screen printing: high at elevated solids loading. DIW: high after concentration‐induced network formation. Spray/blade coating: high. Aerosol jet: moderate after atomization‐compatible formulation.	[99, 100, 101]
**LiF/HCl in situ etching/intercalation**	Fluoride generated in a milder acidic medium, with simultaneous intercalation assistance	Lower handling risk than concentrated HF; effective delamination; large and relatively high‐quality flakes commonly obtained; suitable for additive‐free aqueous dispersions	Remains fluoride‐containing; extensive washing is required; termination chemistry remains route‐sensitive; large flakes may obstruct fine nozzles	Particularly suitable for concentrated aqueous inks and processes benefiting from large‐flake percolation, although size fractionation may be needed for fine‐nozzle printing	Inkjet: moderate after centrifugation or size fractionation. EHD: moderate to high after flake‐size and electrical‐property optimization. Screen printing: high. DIW: high. Spray/blade coating: high. Aerosol jet: moderate to high after atomization‐compatible size control.	[95]
**Electrochemical etching**	Bias‐assisted selective removal of the A‐element, sometimes combined with simultaneous delamination or intercalation	Potentially reduced chemical hazard; controllable etching conditions; opportunities for continuous processing and improved batch reproducibility	Process conditions and product quality are not yet standardized; delamination efficiency and surface chemistry vary among reported methods; limited printing‐specific demonstrations	Promising for scalable ink manufacture, provided that reproducible delamination, termination control, and colloidal stabilization are achieved	Inkjet: conditional, dependent on stable delamination and narrow flake‐size distribution. EHD: conditional to moderate. Screen printing: moderate after concentration and rheology adjustment. DIW: moderate where concentrated. Spray/blade coating: moderate to high. Aerosol jet: conditional, requiring atomization‐compatible dispersions.	[102, 103]
**Molten‐salt‐assisted routes**	Salt‐mediated etching, element replacement, or synthesis under a controlled high‐temperature environment	Opportunities for controlled or more uniform surface terminations; defect and morphology engineering; reduced reliance on direct HF use; potential access to MXenes difficult to obtain through aqueous etching	High‐temperature processing; residual‐salt removal; frequently requires additional delamination, intercalation, or surface conversion; colloidal ink preparation remains less mature	Particularly promising where termination control, thermal stability, or low‐fluorine surfaces are required, but additional processing is generally needed before solution printing	Inkjet: low to conditional unless efficient delamination and size selection are achieved. EHD: conditional because colloidal stability and electrical relaxation are optimized. Screen printing: moderate after conversion into concentrated pastes. DIW: moderate where stable high‐solids dispersions are achievable. Spray/blade coating: moderate after suitable dispersion treatment. Aerosol jet: conditional.	[104, 105]
**Bottom‐up growth or CVD‐like synthesis**	Direct growth of MXene or MXene‐like layers rather than etching and delaminating a bulk precursor	Potentially cleaner surfaces; improved structural and thickness control; reduced dependence on wet‐chemical etching; attractive for high‐quality electronic interfaces	Generally substrate‐bound rather than colloidal; limited production area and throughput; not presently suited to large‐volume ink manufacture	Better suited to direct‐growth electronics, transfer processes, and patterned thin‐film fabrication than to conventional solution‐based printing	Inkjet: low unless the grown material is subsequently released and dispersed. EHD: low for conventional ink‐based EHD printing. Screen printing: low. DIW: low. Spray/blade coating: low for solution coating. Direct patterning/transfer printing: high relative compatibility.	[51]

^a^ Compatibility indicates the typical suitability of MXenes produced by each route after appropriate downstream processing, including washing, delamination, flake‐size selection, solvent exchange, concentration adjustment, and rheological modification. Actual printability depends more directly on flake dimensions, dispersion stability, viscosity, surface tension, electrical conductivity, and solids loading than on the synthesis route alone [17, 106].

MXenes are synthesized via selective etching of the “A” element from their parent M*_n_*_+__1_AX*_n_* phases, taking advantage of the comparatively weak M─A bonds versus the robust M─X bonds [95, 107]. In a typical protocol, Ti_3_AlC_2_ powders are immersed in a solution of hydrofluoric acid (HF, ∼48 wt.%) at room temperature for 24 h. The reaction involves the selective removal of the Al layers (Equations (1), (2), (3), (4)) and introduces surface terminations (*T_x_* = ─F, ─OH, ─O) (Figure 3a) [48, 108, 109, 110, 111].

(1) $${\mathrm{Ti}}_{3}{\mathrm{AlC}}_{2}+3\mathrm{HF}\to {\mathrm{Ti}}_{3}C_{2}+{\mathrm{AlF}}_{3}+3/2H_{2}$$

(2) $${\mathrm{Ti}}_{3}C_{2}+2H_{2}O\to {\mathrm{Ti}}_{3}C_{2}{\left(\mathrm{OH}\right)}_{2}+H_{2}$$

(3) $${\mathrm{Ti}}_{3}C_{2}+2H_{2}O\to {\mathrm{Ti}}_{3}C_{2}{\left(O\right)}_{2}+2H_{2}$$

(4) $${\mathrm{Ti}}_{3}C_{2}+2\mathrm{HF}\to {\mathrm{Ti}}_{3}C_{2}F_{2}+H_{2}$$

**FIGURE 3:**
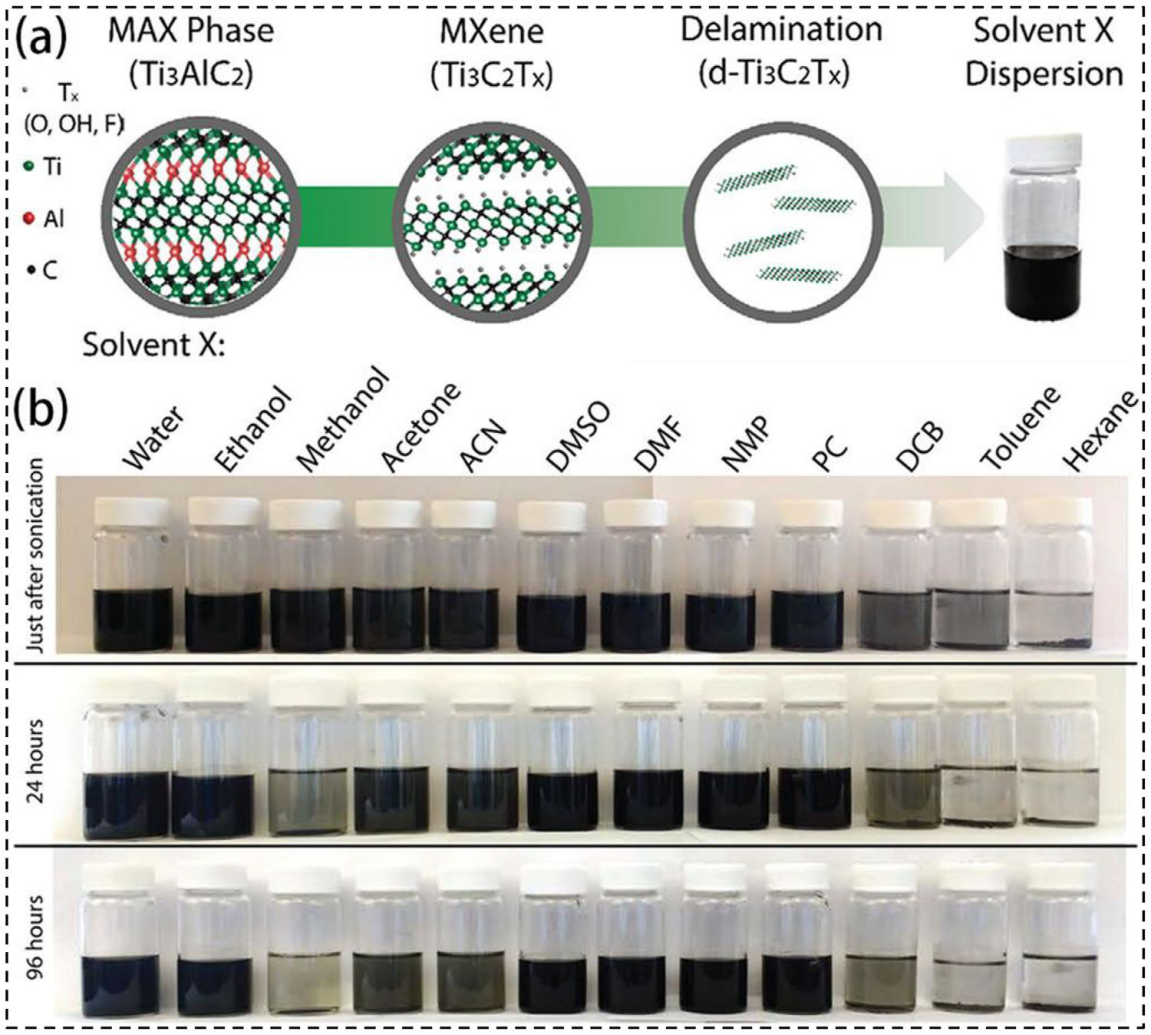
(a) Schematic illustration of the MXene synthesis and dispersion process: selective etching of Al from the Ti_3_AlC_2_ MAX phase yields multilayer Ti_3_C_2_T*_x_* MXene, followed by delamination to form single‐ or few‐layer d‐Ti_3_C_2_T*_x_* nanosheets, subsequently dispersed in various organic solvents (denoted as Solvent X). (b) Photographic comparison of Ti_3_C_2_T*_x_* MXene dispersions in different solvents immediately after sonication, after 24 h, and after 96 h. Reproduced with permission [112]. Copyright 2017, ACS.

After repeated centrifugation and washing to neutralize the pH, the resulting multilayer Ti_3_C_2_T*_x_* sediment could be redispersed in common solvents such as DMSO and gently stirred for 12 h, allowing the solvent molecules to intercalate between the MXene sheets. A subsequent mild sonication step delaminates the MXene stacks into predominantly single‐ and few‐layer flakes, yielding stable colloidal suspensions [11].

To formulate inks suitable for printing or thin‐film deposition, the delaminated Ti_3_C_2_T*_x_* nanosheets could be dispersed in a range of organic and aqueous solvents at 1–2 mg/mL. Figure 3b tracks dispersion stability in solvents spanning dielectric constants ε from 1.9 (hexane) to 78 (water). Immediately after sonication, all polar media (water, DMSO, DMF, NMP, ethanol, acetonitrile) produce uniformly dark suspensions, whereas low‐polarity solvents (toluene, hexane, acetone) appear hazy or partly flocculated. After 24 h, some suspensions begin to sediment, and by 96 h, only few solvents retain their initial colloidal concentration. This solvent‐dependent behavior correlates with (i) dielectric screening of inter‐flake electrostatic repulsion, (ii) hydrogen‐bonding interactions with ─OH terminations, and (iii) solvation of fluorine‐ and oxygen‐terminated surfaces. The clear disparity in aging profiles underscores the necessity of matching solvent properties to MXene surface chemistry when engineering inks for high‐resolution printing, layer‐by‐layer assembly, and scalable device fabrication.

The development of MXene functional inks is a multifaceted challenge that encompasses both the precise control of ink rheology and the targeted modification of MXene surface chemistry. At the heart of this endeavor lies the necessity to tailor MXene nanosheets for enhanced dispersion stability, controlled inter‐flake interactions, and improved compatibility with a range of printing technologies. This is achieved through a variety of functionalization strategies that modify the surface terminations of MXenes, thereby directly influencing their physicochemical properties and the performance of the resulting inks. Different types of studies on MXene‐based functional inks, printing parameters, and applications have been enlisted and summarized (Table 2). These representative studies on MXene functional inks outline printing techniques, formulation strategies, solvents, MXene type and concentration, substrates, and target applications.

**TABLE 2:** Summary of reported MXene‐based functional inks with printing methods, formulations, substrates, and applications.

Printing technique	Additive (binder)	Solvent	MXene type	Substrate	Application	Ref
**Direct ink writing (extrusion)** – also inkjet in the same work	No binder	DI water; NMP	Ti_3_C_2_T*_*x*_*	Plastic/paper	MSCs	[22]
**Direct ink writing**	No binder	DI water	Ti_3_C_2_T_*x*_	Glass/polymer films/paper, and Si/SiO_2_ wafer	MSCs	[89]
**Direct ink writing (high‐precision)**	No binder	DI water	Ti_3_C_2_T_*x*_	Flexible substrates	Wireless sensing electronics	[94]
**Direct ink writing** **(3D)**	AlOOH nanoparticle additive	DI water	Ti_3_C_2_T*_x_*	Glass	EMI shielding aerostructures	[113]
**Direct ink writing** **(3D)**	PEDOT: PSS	DI water; ethylene glycol	Ti_3_C_2_T*_x_*	PET	MSCs	[40]
**Stamp/hand printing** (pen writing)	No binder	DI water	Ti_3_C_2_T_*x*_	Paper/textile/wood/plastic	Flexible circuits	[114]
**Stamp**	No binder	DI water	Ti_3_C_2_T*_x_*	Plate, PET paper, glass	MSCs	[92]
**Stamp**	No binder	DI water	Ti_3_C_2_T*_x_*	PET/SiO_2_	MSCs	[92]
**Stamp**	No binder	DI water	Ti_3_C_2_T*_x_*	Paper	MSCs	[92]
**Screen printing**	Lithium titanate and lithium iron phosphate	DI water	Ti_3_C_2_T_*x*_	A4 paper, wood, fabric, SiO*_x_*‐coated stainless steel	MSCs	[46]
**Screen printing**	Xanthan gum	DI water	Ti_3_C_2_T_*x*_	PET, PI, paper	EMI shielding, pressure sensor, Joule heater	[115]
**Screen printing**	No binder	DI water	Ti_3_C_2_T_*x*_	PET, PI, Photographic Paper….	RFID tag antenna	[116]
**Screen printing**	Acetylene black and polyvinylidene fluoride	Di water	Ti_3_C_2_T_*x*_	A4 paper/PET films	Hybrid devices for force sensor	[117]
**Screen printing**	Functionalized with chitosan	DI water	Ti_3_C_2_T*_x_*	Carbon disk	Detection of sarcosine (SAR)	[118]
**Electrohydrodynamic (EHD) printing**	Alkyl functionalization	Ethanol	Ti_3_C_2_T*_x_*	SiO_2_/Si	Thin‐film transistors (TFTs)	[38]
**Electrohydrodynamic (EHD) printing**	No binder	Ethanol	Ti_3_C_2_T*_x_*	SiO_2_	Field‐effect transistor for probing aflatoxin B1	[119]
**Electrohydrodynamic (EHD) printing**	Hydrogel precursor solutions	Glycerol / DI water	Ti_3_C_2_T*_x_*	Flexible ecoflex mold	Flexible strain sensor	[120]
**Inkjet printing**	No binder	Ethanol	Ti_3_C_2_T*_x_*	silicate glass/SiO_2_/Si/PET	Ultrafast photonics devices	[121]
**Inkjet printing**	No binder	DI water	Ti_3_C_2_T*_x_*	PET	Transparent conductive films	[122]
**Spray coating** (airbrush deposition)	No binder	DI water	Ti_3_C_2_T*_x_*	PET	Tunable EMI shielding coating	[123]
**Spray coating**	Gelatin	DI water	Ti_3_C_2_T*_x_*	Paper	3D Origami based NH_3_ gas sensor	[124]
**Spray coating**	Graphene and PVP	DI water	Ti_3_C_2_T*_x_*	PET	Terahertz shielding	[125]

The comparison underscores how ink design, ranging from binder‐free dispersions to polymer composites, directly influences printability, substrate compatibility, and device performance, providing a practical reference for tailoring MXene inks to specific application needs. Furthermore, these functionalization strategies can be divided into:

### 2.1 Intrinsic Surface Chemistry, Colloidal Behavior, and Interfacial Interactions

The intrinsic surface chemistry of MXenes, typically dominated by ─OH, ─O, and ─F terminations, plays a decisive role in their interparticle interactions, colloidal stability, and environmental behavior. The negative zeta potential associated with these surface groups generates electrostatic repulsion between adjacent flakes and promotes initial dispersion stability in aqueous media. However, oxidation is primarily governed by reactive edge sites, structural defects, exposed transition‐metal sites, dissolved oxygen, water, temperature, and solution chemistry, rather than by zeta potential itself. Colloidal stability is further influenced by pH and ionic strength, which alter the protonation of surface terminations and the thickness of the electrical double layer. Increasing ionic strength, particularly in the presence of multivalent ions, can screen electrostatic repulsion and promote flake aggregation, restacking, or sedimentation. Flake size, aspect ratio, and concentration also govern dispersion behavior, with large nanosheets tending to form interconnected or liquid‐crystalline networks, while smaller flakes generally remain more readily dispersed but contain a higher proportion of reactive edge sites. These colloidal characteristics directly influence ink viscosity, yield behavior, storage stability, nozzle reliability, and printed‐film uniformity. Approaches to counteract oxidation include:

- *Controlled etching and post‐treatment*: Adjusting etching conditions (e.g., using milder or fluoride‐free etchants, electrochemical or molten salt methods) can modulate the relative concentrations of surface terminations. Subsequent treatments such as vacuum annealing can selectively remove ─OH groups, thereby enhancing electronic conductivity [126, 127, 128].
- *Protective functional layers*: Surface functionalization with hydrophobic agents (e.g., Perfluorooctyl trichlorosilane (FOTS)) has been shown to effectively shield MXene flakes from water uptake [129].
- *Antioxidant and pH control*: The incorporation of antioxidants (e.g., sodium *L‐*ascorbate) or the use of polyanionic salts can cap reactive edge sites, suppress oxidation, and extend the colloidal stability of MXene inks [130, 131, 132, 133].

In addition to oxidation‐control strategies, alternative functionalization approaches have been developed to simultaneously tailor the rheological, colloidal, and interfacial properties of MXene inks. These approaches include ligand‐based surface modification and the intercalation of ions, such as H^+^, Li^+^, and Na^+^, or organic molecules between adjacent MXene layers. Intercalation expands the interlayer spacing, weakens intersheet interactions, facilitates delamination, and improves dispersion quality. In simultaneous etching–intercalation processes, intercalating species are introduced during selective removal of the A‐element from the MAX phase, enabling etching, layer expansion, and surface modification within an integrated processing route. Compared with harsher direct‐etching conditions, carefully controlled etching–intercalation can reduce structural damage and produce relatively large, high‐quality flakes with tunable surface chemistry. These surface and structural modifications not only govern dispersion stability and ink rheology but also determine the strength and nature of interactions formed between printed MXene layers and their supporting substrates [30, 55].

Interfacial adhesion is therefore a critical factor controlling the mechanical robustness, electrical continuity, and long‐term reliability of printed MXene devices. At the molecular level, adhesion arises through several complementary mechanisms that depend on MXene surface terminations, functional ligands, binder chemistry, and substrate composition [134]. The abundant ─OH and ─O terminations on MXene surfaces can form hydrogen bonds with hydroxyl‐rich substrates, including cellulose, paper, cotton, and poly(vinyl alcohol)‐based surfaces. Catechol‐containing ligands, silane coupling agents, and other interfacial modifiers can further promote coordination‐assisted or covalent anchoring, particularly on oxidized or oxide‐containing surfaces [135]. Electrostatic attraction and ionic crosslinking may also strengthen the interface when negatively charged MXene flakes interact with positively charged polymers, polyelectrolytes, or multivalent ions. In binder‐containing formulations, polymer‐chain entanglement and physical crosslinking enhance stress transfer, improve cohesion, and suppress delamination [136]. Porous and fibrous substrates, such as paper and textiles, additionally promote mechanical interlocking through ink infiltration and anchoring within the substrate microstructure [137]. In contrast, comparatively inert polymer substrates, including PET, PI, and particularly low‐surface‐energy PDMS, often require plasma treatment, UV–ozone activation, primers, or adhesive interlayers to achieve durable adhesion. Collectively, these chemical and physical interactions govern film continuity, interfacial and contact resistance, bending durability, and environmental stability in printed MXene electronics [138, 139, 140].

Although intrinsic surface chemistry provides MXenes with favorable colloidal and interfacial characteristics, unmodified dispersions may not satisfy the rheological, stability, and substrate‐compatibility requirements of all printing processes. Intentional strategies involving intercalants, ligands, surfactants, polymers, and other additives are therefore employed to tailor these properties, as discussed in Section 2.2.

### 2.2 Engineered Surface Functionalization for Stability and Performance Control

Chemical modification extends beyond the control of oxidation and dispersion stability to include the fine‐tuning of MXene's electronic, mechanical, and interfacial properties. Some of the techniques include:

- Organic ligands functionalization: A primary approach involves the use of organic ligands to modify the MXene surface. By exploiting the inherent reactivity of surface terminations such as ─OH, ─O, and ─F, ligands like catechol derivatives have been employed to create noncovalent interactions that significantly enhance dispersion stability in both aqueous and organic solvents. Alkylated 3,4‐dihydroxy‐L‐phenylalanine (ADOPA), for instance, adsorbs spontaneously via catechol and π–π interactions, leading to enhanced dispersion in organic solvents and prolonged environmental stability even under ambient conditions. 3,4‐dihydroxy‐L‐phenylalanine (DOPA) and ADOPA have been shown to rapidly functionalize MXene surfaces, simultaneously introducing a hydrophilic catechol head and a hydrophobic fluoroalkyl tail (Figure 4a). This dual functionality not only stabilizes the dispersion in volatile organic media, thus mitigating issues such as the coffee‐ring effect during printing, but also preserves high electrical conductivity by minimizing inter‐flake resistances. The resultant ADOPA‐functionalized MXene inks exhibit excellent oxidation resistance and maintain their conductive properties even under challenging environmental conditions. In a different approach, a non‐ionic surfactant (Hexaethylene glycol monododecyl ether, C_12_E_6_) could be adsorbed onto Ti_3_C_2_T_*x*_ lamellae, enabling the formation of a lyotropic liquid‐crystalline phase in which MXene sheets align along a common direction (Figure 4b). Such ordered assemblies are advantageous for producing free‐standing, mechanically robust films with anisotropic electrical and mechanical properties. Another study reports an aqueous, anaerobic strategy for tailoring exfoliated Ti_3_C_2_T_*x*_ MXene via spontaneous catechol functionalization. Beginning with Ti_3_AlC_2_‐derived Ti_3_C_2_T_*x*_, the freeze‐dried MXene powders are re‐suspended in degassed water, then mixed with deoxygenated dopamine (DA) or pyrocatechol (PCAT) solutions. Figure 4c illustrates the synthesis and surface modification of Ti_3_C_2_T_*x*_ MXene via catechol binding. The catechol molecules are anchored to the MXene surface through various interaction modes, including hydrogen bonding, mixed hydrogen/covalent bonding, covalent monodentate, and covalent bidentate coordination with surface Ti sites. Functional catechol derivatives such as DA and PCAT enable further tuning of MXene's surface chemistry, enhancing stability, dispersibility, and compatibility with diverse solvent systems.
- Macromolecule functionalization: Leveraging the abundant ─OH groups, in situ polymerization of monomers (e.g., pyrrole or DMAEMA) on MXene surfaces can be initiated to form polymeric networks intercalated between MXene layers. This strategy reinforces the mechanical integrity of MXene films and provides a platform for subsequent functionalization, as demonstrated in MXene/polymer composites, where hydrogen bonding and electrostatic interactions foster well‐defined core–shell architectures. Figure 5 illustrates three representative strategies for integrating Ti_3_C_2_T*_x_* MXenes with conductive or structural polymer matrices to enhance their processability, mechanical durability, and electronic properties for multifunctional applications. Figure 5a highlights the in situ chemical polymerization of pyrrole monomers onto MXene surfaces to form polypyrrole (PPy) coatings, which improve interlayer conductivity and provide electrochemical stability. The molecular‐level interaction between the conjugated polymer and the MXene surface fosters electron delocalization and structural cohesion. In Figure 5b, the fabrication of MXene/PANI composites is shown: Initially, the MXene flakes were produced via selective etching of Ti_3_AlC_2_ and then dispersed in aqueous solution and reacted with aniline monomers under acidic and low‐temperature conditions to yield PANI‐coated MXene films. The resulting freestanding films combine the high conductivity of MXenes with the flexibility and electroactivity of PANI, making them suitable for supercapacitor and wearable electronics applications. Figure 5c presents a hybridization approach with cellulose nanofibers (CNFs), leveraging the mechanical reinforcement and film‐forming capabilities of CNFs. Delaminated Ti_3_C_2_T*_x_* nanosheets are blended with CNFs to form homogeneous composite suspensions that can be vacuum filtered into dense, uniform films. The visual and microscopic characterization in the lower row confirms the successful integration of the two phases. Collectively, these composite strategies offer scalable paths to robust, multifunctional MXene‐based materials for next‐generation flexible electronics, energy storage, and structural applications. It is important to note that optimized MXene chemistry is crucial in developing homogeneous dispersions or stable inks that could be transformed to create well‐structured composite materials.
  These polymer‐based approaches yield composites with improved interfacial adhesion, controlled stacking, and tunable porosity, making them particularly attractive for applications in energy storage, catalysis, and sensor devices.
- Doping and heteroatom incorporation for enhanced performance: In addition to catechol‐based functionalization and macromolecular integration strategies illustrated in Figures 4 and 5, heteroatom doping has emerged as a powerful method to tailor the physicochemical properties of MXenes at the atomic scale. By introducing non‐metal dopants such as nitrogen (N), phosphorus (P), or sulfur (S) into the MXene lattice or onto its surface, it is possible to modulate the local electronic structure, surface chemistry, and interlayer environment in ways that surpass the capabilities of surface terminations alone. For instance, N‐doping of Nb_2_CT_*x*_ and V_2_CT_*x*_ MXenes achieved via hydrothermal treatment in ammonia solutions or high‐temperature annealing under NH_3_ has been shown to expand the interlayer spacing, thereby facilitating faster ion diffusion pathways in energy storage applications. This structural expansion reduces steric hindrance for electrolyte ions, a particularly important factor for high‐rate lithium‐ion and sodium‐ion batteries. Simultaneously, N atoms introduce localized electronic states near the Fermi level, increasing the density of states and improving charge carrier mobility, which translates into higher electrical conductivity and faster charge–discharge kinetics.

**FIGURE 4:**
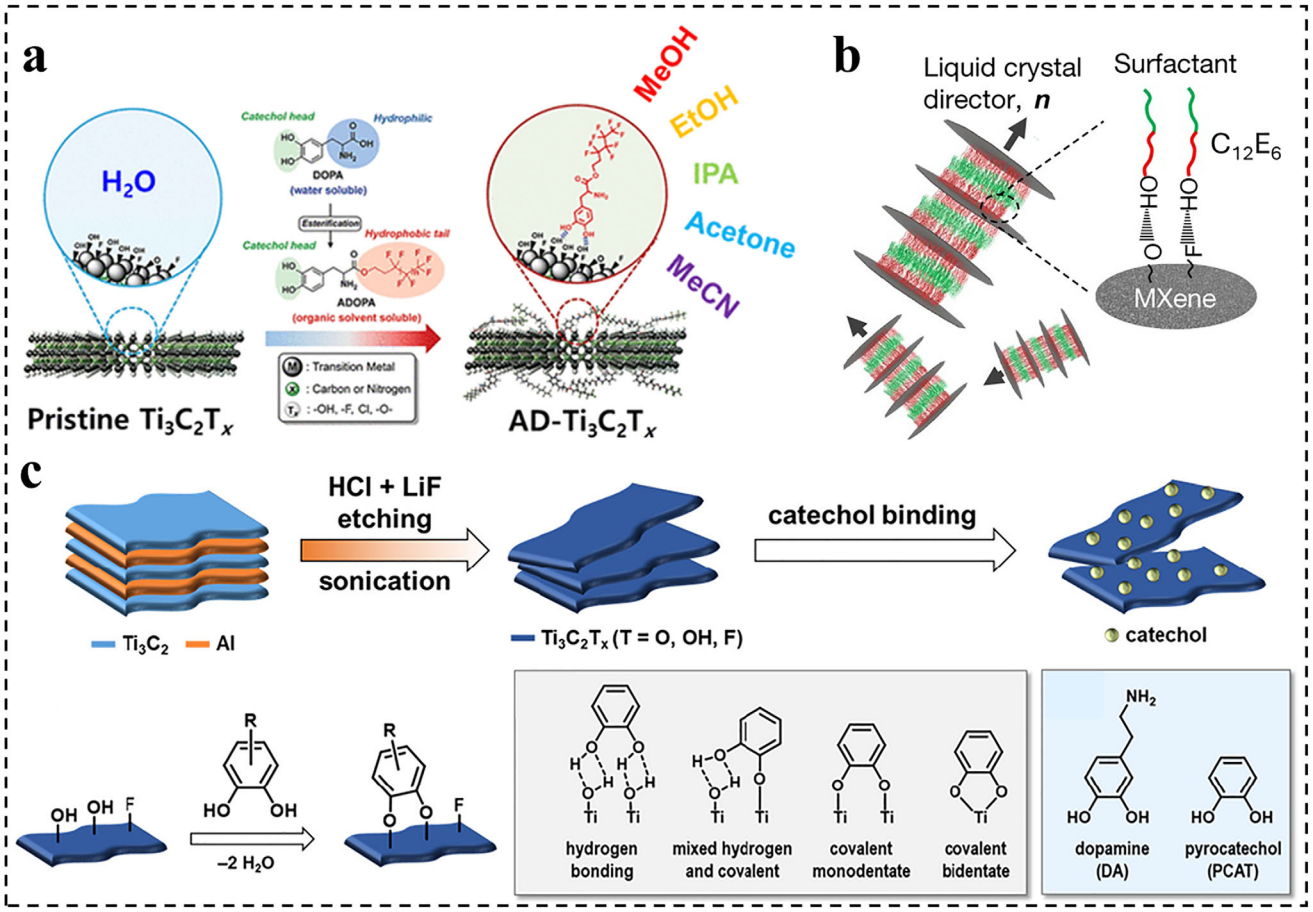
Surface Modification and Composite Fabrication Strategies for Ti_3_C_2_T*_x_* MXene; (a) Schematic of catechol‐based amphiphilic ligand (ADOPA) grafting onto pristine Ti_3_C_2_T*_x_*. Reproduced with permission [30]. Copyright 2022, ACS. (b) Illustration of liquid‐crystalline self‐assembly of C_12_E_6_‐modified MXene sheets. Reproduced with permission [141]. Copyright 2018, Nature. (c). Workflow for MXene delamination and ligand functionalization: (i) etching of Ti_3_AlC_2_ with HCl + LiF to produce multilayer Ti_3_C_2_T_*x*_, (ii) DMSO intercalation and mild sonication to yield few‐layer flakes (d‐Ti_3_C_2_T*_x_*), and (iii) subsequent catechol binding to form AD‐Ti_3_C_2_T*_x_*, followed by representative catechol‐MXene binding motifs: pure hydrogen bonding; mixed hydrogen/covalent coordination; covalent monodentate; and covalent bidentate interactions along with chemical structures of the two catechol ligands used: dopamine (DA) and pyrocatechol (PCAT). Reproduced with permission [31]. Copyright 2021, ACS.

**FIGURE 5:**
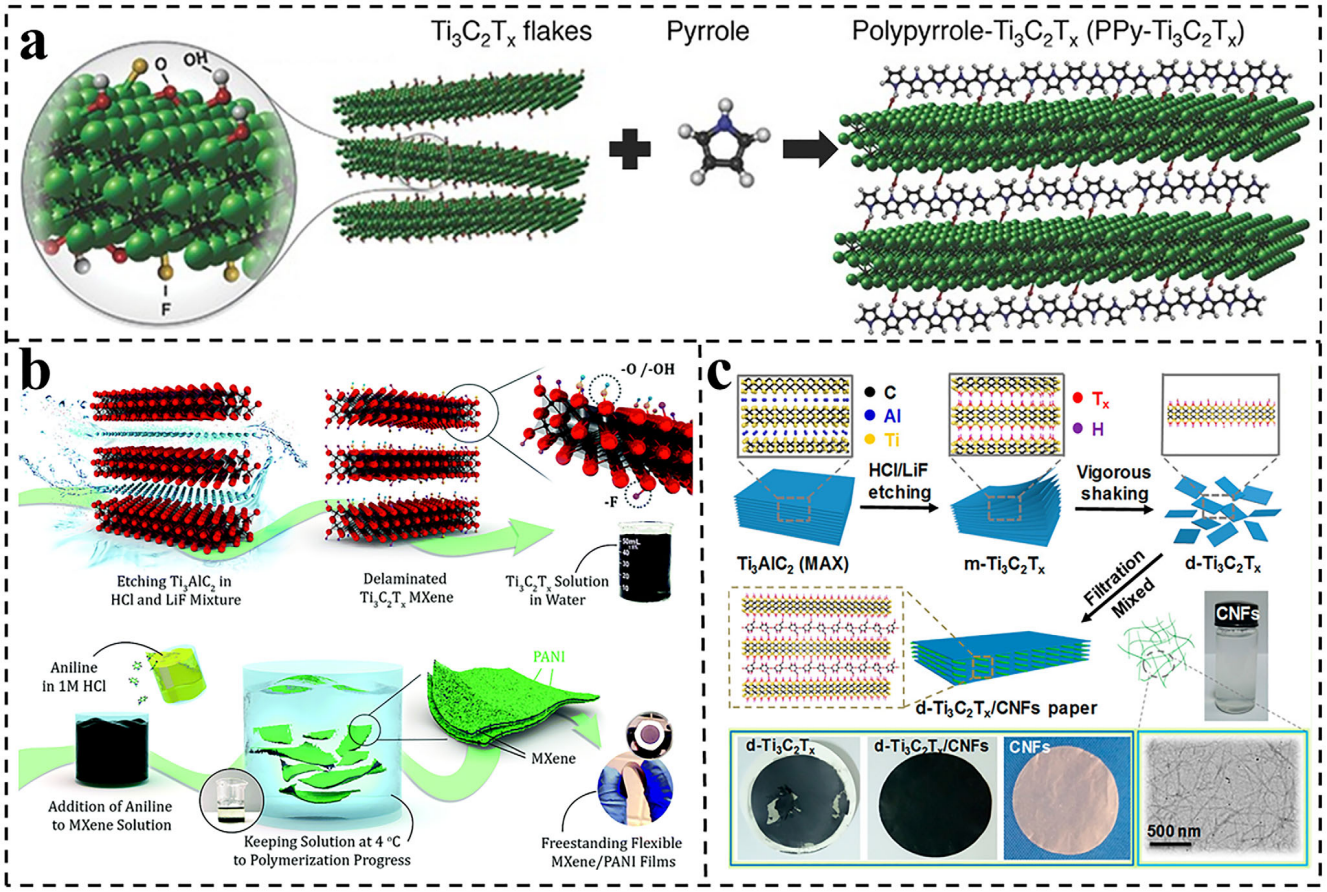
MXene‐polymer composites for enhanced mechanical and electrical properties; (a) Schematic representation of in situ polymerization of pyrrole monomers in the presence of Ti_3_C_2_T*_x_* flakes forming polypyrrole‐coated MXene nanosheets (PPy‐Ti_3_C_2_T*_x_*). Reproduced with permission [142]. Copyright 2016, Wiley. (b) Stepwise synthesis of polyaniline (PANI)‐functionalized MXene: Ti_3_AlC_2_ is etched using an HCl/LiF mixture to produce multilayer Ti_3_C_2_T*_x_*, which is subsequently delaminated in water. Reproduced with permission [143]. Copyright 2018, RSC. (c) Fabrication route for MXene‐cellulose nanofiber (CNF) hybrid films: MAX phase (Ti_3_AlC_2_) is etched and delaminated to form d‐Ti_3_C_2_T*_x_* nanosheets. These are mixed with CNFs under vigorous stirring and filtered into uniform d‐Ti_3_C_2_T*_x_*/CNF composite films. Reproduced with permission [144]. Copyright 2018, ACS.

Phosphorus doping, often introduced through phosphoric acid treatment or red phosphorus thermal decomposition, can induce electron‐rich sites that enhance catalytic activity for reactions such as hydrogen evolution and oxygen reduction (ORR). Similarly, sulfur doping, performed through thiourea decomposition or H_2_S treatment, has been observed to increase the hydrophobicity of MXene surfaces, improving their compatibility with non‐aqueous electrolytes and expanding their functionality in flexible supercapacitors and lithium–sulfur batteries. When integrated with molecular‐level functionalization, such as catechol binding, heteroatom doping enables a synergistic enhancement of performance. The catechol moieties improve colloidal stability and dispersion across various solvents, while the dopants intrinsically alter the MXene's conductive and structural properties. This multi‐tiered modification approach has yielded electrodes with significantly higher areal capacitances, improved cycling stability, and superior rate capability compared to their pristine counterparts [145]. Such results underscore the potential of combining electronic tuning via doping with interface engineering through surface chemistry, paving the way for next‐generation MXene‐based materials optimized for high‐performance, large‐scale energy and electronic applications.

## 3 Formulation of Inks and Comparison of Different Printing Techniques

The formulation of MXene inks is inherently multifactorial, relying on a synergistic balance among active MXene flakes, binders, additives, and solvents. In an additive‐free formulation, MXene serves as both the functional and conductive agent, while water‐based or organic solvents ensure dispersion stability and optimal rheological properties. The ink's viscosity, governed by parameters such as solid content, nanoplate size, thixotropy, pH, and temperature, is critical for printing methods ranging from inkjet and spray coating to screen and DIW printing. Dimensionless groups (Reynolds, Weber, and Ohnesorge numbers) provide a quantitative basis for predicting droplet formation and pattern resolution [42]. Adjustments to these parameters, for example, through tailored solvent polarity or binder removal, can mitigate common defects like the “coffee ring” effect by promoting uniform deposition via mechanisms such as Marangoni flow.

### 3.1 Ink Rheology and Stability

MXene inks exhibit both pseudoplastic (shear‐thinning) and thixotropic behavior. In shear experiments, the relationship between viscosity (*η*) and shear rate is pivotal; shear thinning allows the ink to flow under the high shear rates imposed by a printing nozzle while rapidly recovering its high viscosity upon deposition. This duality is crucial for maintaining patterned structures post‐printing. Viscoelastic parameters, specifically the elastic (*G*′) and viscous (*G*″) moduli, provide further insights. A higher *G*′ relative to *G*″ under low shear stress indicates solid‐like behavior, which is beneficial in processes that require shape retention (e.g., extrusion printing). Conversely, dominance of *G*″ supports a liquid‐like state conducive to rapid spreading and film formation (e.g., spray coating) [19, 44, 146]. Notably, the transition from elastic‐dominated to viscous‐dominated behavior often occurs at a critical yield stress. For example, single‐layer MXene dispersions have demonstrated yield stresses ranging from as low as 24 Pa (derived via the Herschel–Bulkley model) to values exceeding 200 Pa when the concentration is increased or when MXene flakes with higher aspect ratios are employed.

The rheological properties of MXene inks are strongly dependent on ink concentration and the morphological characteristics of the MXene flakes. Systematic studies have revealed that both single‐layer and multilayer dispersions exhibit shear‐thinning behavior; however, the viscosity increases markedly with concentration. In multilayer dispersions, the viscosity at high shear rates may span three orders of magnitude between dilute and highly concentrated systems. The critical influence of rheological properties on the printability and performance of Ti_3_C_2_T*_x_* MXene inks across various deposition methods has been explained in Figure 6. Printable MXene formulations must strike a delicate balance between fluid‐like and solid‐like behaviors, characterized by a sufficiently high storage modulus (*G*′) that imparts structural integrity and shape retention, coupled with a yield stress low enough to enable controlled flow through nozzles under moderate shear conditions (Figure 6a). A hallmark of printable MXene inks is pronounced shear‐thinning, a phenomenon driven by the progressive disruption of interconnected flake networks upon application of shear. Such shear‐induced network breakdown facilitates smooth flow during printing, followed by rapid structural recovery when shear forces cease, ensuring printed features maintain their shape without spreading or sagging. Figure 6b,c illustrates viscosity profiles of single‐layer and multilayer Ti_3_C_2_T*_x_* inks at different solids loadings, underscoring the strong correlation between increased MXene concentration and intensified shear‐thinning behavior. Single‐layer MXene dispersions at moderate concentrations, making them particularly suitable for inkjet printing, spray coating, and wet spinning. In contrast, highly concentrated multilayer suspensions (50–70 wt.%) exhibit significantly elevated viscosities and storage moduli, limiting their utility primarily to extrusion‐based deposition methods, such as DIW or 3D printing, where the robust elastic properties (high *G*′/*G*″ ratios) are essential for shape fidelity and mechanical integrity post‐printing.

**FIGURE 6:**
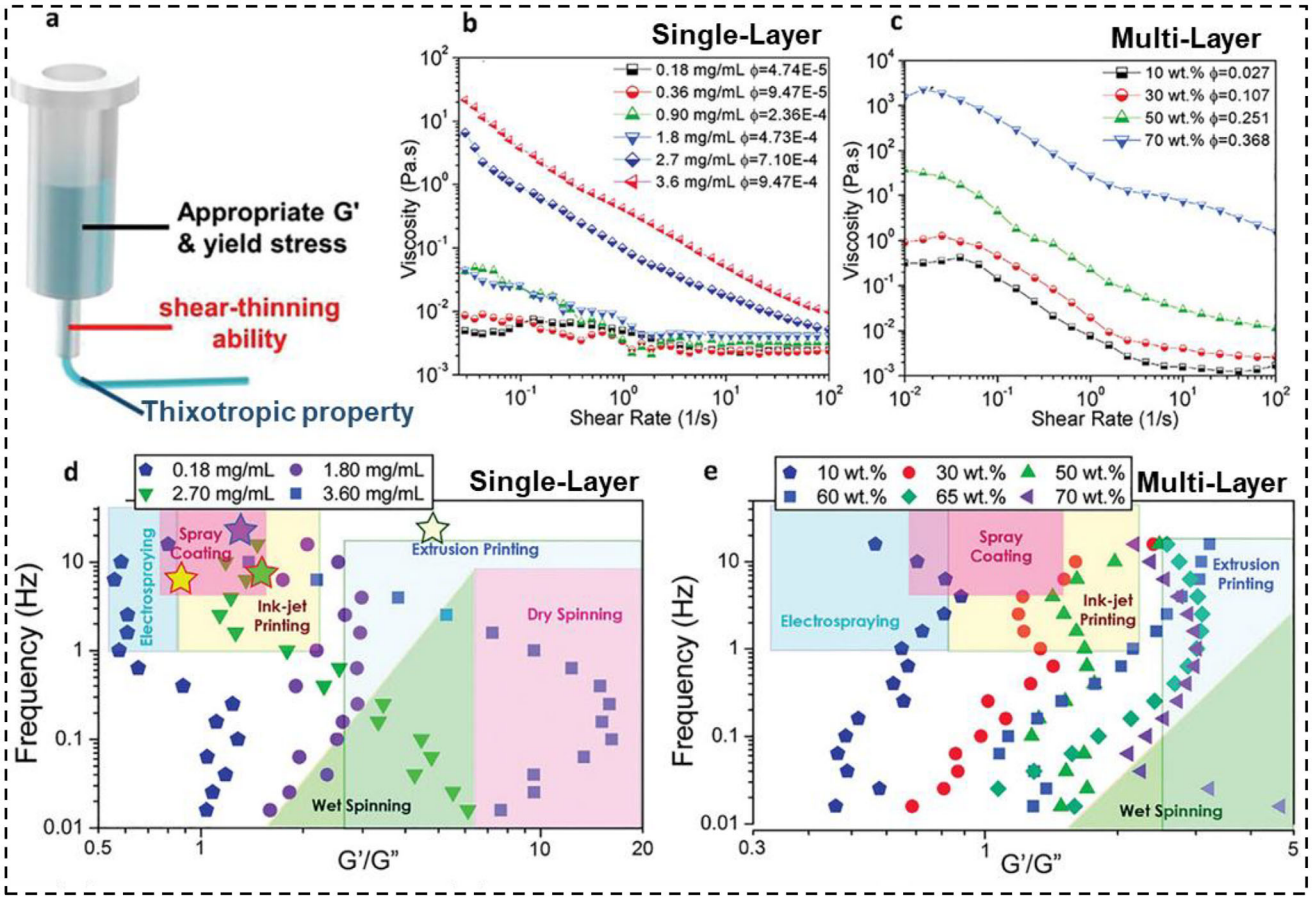
Rheological characteristics and printability of MXene inks: (a) Schematic requirements for ideal ink rheology, including appropriate storage modulus (*G*′), yield stress, shear‐thinning behavior, and thixotropic recovery. Reproduced with permission [147]. Copyright 2022, Wiley. (b) Viscosity profiles of single‐layer Ti_3_C_2_T*_x_* MXene inks with varying concentrations (0.18–3.6 mg/mL) as a function of shear rate, exhibiting concentration‐dependent shear‐thinning behavior. (c) Viscosity profiles of multi‐layer MXene dispersions with different solid contents (10–70 wt.%) highlight enhanced viscosity and shear‐thinning at higher concentrations. Printability windows for (d) single‐layer and (e) multi‐layer Ti_3_C_2_T*_x_* dispersions across different techniques (e.g., inkjet printing, spray coating, extrusion printing), plotted as frequency versus *G*′/*G*″ ratios. Each printing method corresponds to specific viscoelastic and frequency requirements. Reproduced with permission [33]. Copyright 2018, ACS.

To make these rheological descriptors practically useful for ink selection, Table 3 summarizes representative printability windows for the major MXene‐compatible printing techniques. These ranges should be interpreted as approximate processing windows rather than universal limits, because the optimal values depend strongly on MXene flake size, concentration, solvent composition, binder content, surface functionalization, substrate wettability, and drying conditions. For droplet‐based methods such as inkjet printing, stable printing is commonly evaluated using dimensionless groups such as the Reynolds number, Weber number, Ohnesorge number, and inverse Ohnesorge number. In contrast, extrusion‐based methods such as DIW and screen printing are governed more strongly by yield stress, shear‐thinning index, storage/loss moduli, and thixotropic recovery. EHD printing occupies an intermediate regime because electrostatic stress can overcome some viscosity limitations of conventional inkjet printing, although cone‐jet stability, charge relaxation, nozzle‐substrate gap, and flow‐rate control become critical. Therefore, a complete assessment of MXene ink printability requires technique‐specific rheological metrics rather than a single universal *Z*‐number criterion.

**TABLE 3:** Critical factors affecting ink performance.

Printing/coating method	Viscosity range (mPa s)	Surface tension range (mN/m)	printability metrics	rheological behavior	limitations	Ref.
**Inkjet printing**	∼1–30	∼25–45	Oh, *Z *= 1/Oh, commonly *Z* ≈ 1–10 for stable droplet formation	Low‐viscosity Newtonian or weakly shear‐thinning ink	Nozzle clogging, satellite droplets, coffee‐ring deposition, limited solids loading	[19]
**Electrohydrodynamic printing**	∼1–10^3^, depends on voltage, flow rate, nozzle diameter, and gap	∼20–50	Taylor‐cone stability, cone‐jet regime, voltage, flow rate, nozzle‐substrate gap, charge relaxation; Oh/Z useful but not sufficient alone	Stable electrically driven meniscus; controlled cone‐jet or pulsating cone‐jet	Jet instability, nozzle clogging, multi‐nozzle electric‐field cross‐talk	[1, 38, 119]
**Direct ink writing/extrusion**	∼10^2^–10^6^	Less restrictive than droplet printing; often ∼30–70 depending on solvent/additives	Yield stress, G′/G″, shear‐thinning index, thixotropic recovery, filament shape retention	Strong shear‐thinning during extrusion and rapid recovery after deposition; sufficiently high G′ and yield stress for shape retention	Filament spreading, collapse, poor layer stacking	[89, 94, 113]
**Screen printing**	∼10^3^–10^5^	∼30–60, substrate‐ and mesh‐dependent	Yield stress, viscosity recovery, mesh release, line‐edge fidelity	Paste‐like pseudoplastic behavior; viscosity decreases under squeegee shear and recovers after mesh release	Mesh clogging, edge spreading, incomplete transfer	[115, 116, 117]
**Spray coating**	∼1–100	∼20–50	Atomization stability, droplet size distribution, Oh, evaporation rate, substrate wetting	Low‐to‐medium viscosity; stable atomization; good wetting and controlled drying	Overspray, roughness, poor edge definition, nonuniform film thickness, coffee‐ring‐like drying defects	[124, 125]
**Blade coating/bar/slot‐die‐related coating**	∼10–10^4^	Wetting‐dependent, commonly ∼20–50	Capillary number, coating gap, coating speed, wet‐film thickness, leveling time, contact angle	Controlled leveling without dewetting; suitable viscosity for continuous film formation	Thickness gradients, edge bead, dewetting	[148, 149]

The intrinsic oxidative susceptibility of aqueous MXene colloids poses a challenge for their long‐term stability and practical deployment. To overcome this issue, MXene inks are frequently formulated in polar organic solvents such as dimethylformamide (DMF), *N*‐methyl‐2‐pyrrolidone (NMP), dimethyl sulfoxide (DMSO), and propylene carbonate (PC), which form protective solvation layers around MXene flakes, significantly enhancing oxidative resistance [22, 112, 150, 151]. Such solvents typically exhibit nearly linear viscosity increases with rising MXene concentrations, promoting predictable and uniform ink flow. Optimal solvents are selected based on surface tensions ranging from approximately 35–42 mN/m and carefully tuned Hansen solubility parameters, ensuring maximized dispersion quality, minimized sedimentation, and prolonged colloidal stability. Interestingly, single‐layer MXene flakes with higher aspect ratios can form interconnected networks or even liquid‐crystalline phases at relatively modest concentrations (∼15 mg/mL), closely resembling the rheological characteristics typically observed in denser multilayer suspensions (∼70 wt.%). This feature allows for the formulation of mechanically robust inks suitable for techniques requiring higher structural stability, such as wet spinning and extrusion printing, without resorting to prohibitively high solid loadings, thus preventing nozzle clogging and simplifying processing. The overall printability and printing outcomes of MXene inks are further optimized through targeted modifications, including the incorporation of surfactants and intercalants like tetrabutylammonium hydroxide (TBAOH). These additives adjust ink surface tension and inter‐flake interactions, effectively controlling droplet formation, substrate wetting behavior, and minimizing undesirable effects such as coffee‐ring formation. Consequently, tailored MXene ink formulations can be systematically developed to meet application‐specific printing requirements, enabling high‐resolution patterning for microscale energy storage devices like micro‐supercapacitors and batteries, as well as facilitating large‐area deposition processes essential for flexible electronics and multifunctional coatings.

### 3.2 AI‐Assisted Rheology and Closed‐Loop Ink Optimization

The formulation of MXene inks involves a high‐dimensional design space in which MXene concentration, flake size, surface termination, solvent composition, binder/additive loading, pH, evaporation rate, and printing parameters jointly determine viscosity, surface tension, yield stress, thixotropic recovery, printed‐line morphology, and electrical performance. Conventional optimization is usually performed by one‐factor‐at‐a‐time screening; however, this approach is inefficient because printability and conductivity often vary nonlinearly and can show competing dependencies. Therefore, AI‐assisted and data‐driven optimization has recently emerged as a useful strategy for accelerating functional ink design. Here, this review presents a prospective workflow informed by machine‐learning applications in related printed‐material systems as shown in Table 4.

**TABLE 4:** AI‐assisted ink rheology for printing systems.

Study/approach	Printing system	ML/data‐driven method	Optimized or predicted variables	Refs.
ML‐assisted E‐jet printing of organic flexible biosensors	EHD/e‐jet printing	Supervised learning; KNN, random forest, AdaBoost	Nozzle speed, flow rate, voltage → printed circuit conductivity	[3]
Physics‐informed Bayesian learning of EHD polymer jet printing	EHD polymer jet printing	Machine vision + physics‐based modeling + Bayesian learning	Jet features and process dynamics for closed‐loop calibration	[152]
ML‐assisted extrusion printing of thermoelectric materials	Extrusion/DIW printing	High‐throughput experimentation + Bayesian optimization + Gaussian process regression	Ink formulation and printing parameters → power factor and shape fidelity	[153]
Bayesian optimization for materials design	Screen printing	Gaussian process regression; expected improvement/knowledge‐gradient methods	Data‐driven processing increases data efficiency	[154]

Machine‐learning‐assisted printing studies have shown that experimental datasets linking formulation/process parameters to printed outcomes can be used to predict conductivity, shape fidelity, and process stability. For example, ML‐assisted electrohydrodynamic jet printing has been used to correlate nozzle speed, ink flow rate, and applied voltage with the electrical conductivity of printed organic flexible biosensors, demonstrating the ability of supervised learning models to guide high‐resolution printed electronics fabrication. Physics‐informed Bayesian learning has also been applied to EHD polymer jet printing, where machine‐vision‐extracted jet features, physics‐based models, and Bayesian learning were combined to enable closed‐loop process calibration. In extrusion‐based printing, high‐throughput experimentation combined with Bayesian optimization and Gaussian process regression has been used to simultaneously optimize ink formulation and printing parameters for printed thermoelectric materials, balancing functional performance with shape fidelity. These studies demonstrate that AI‐assisted ink design can move beyond empirical trial‐and‐error toward predictive and closed‐loop optimization.

For MXene inks, such approaches are particularly valuable because the same formulation variables that improve printability can compromise charge transport. For instance, increasing polymer or surfactant content may improve viscosity recovery, wetting, and adhesion, but can also increase interflake contact resistance and reduce conductivity. A practical AI‐assisted MXene‐ink workflow should therefore use formulation descriptors, including MXene concentration, lateral flake size, termination chemistry, solvent polarity/Hansen parameters, binder fraction, zeta potential, viscosity, surface tension, *G*′/*G*″, yield stress, and drying rate, as input features. The target outputs should include printability window, line width, coffee‐ring suppression, film roughness, sheet resistance, adhesion, cycling stability, and device‐level performance. Multi‐objective optimization methods such as Bayesian optimization are especially suitable because they can balance competing targets, such as high conductivity, stable rheology, fine resolution, long shelf life, and scalable throughput. In this context, AI‐assisted rheology design should be viewed not as a replacement for physical understanding but as a tool for integrating ink chemistry, colloidal stability, process parameters, and device metrics into a single optimization framework.

### 3.3 Conductivity‐Printability Trade‐Off in MXene Inks

A central challenge in MXene ink formulation is balancing printability with electrical conductivity. Polymeric binders, surfactants, and rheology modifiers can improve dispersion stability, viscosity, shear‐thinning behavior, substrate wetting, adhesion, and post‐deposition shape retention. However, excessive additive loading may increase the separation between adjacent MXene flakes, reduce direct interflake contact, introduce resistive interfacial layers, and increase tunneling barriers and interflake contact resistance. Consequently, formulations with improved printing fidelity may exhibit reduced film conductivity and increased sheet resistance. Conductive polymers and strongly interacting ligands can partially preserve charge‐transport pathways, whereas insulating binders generally require strict concentration control. Additive‐free or minimal‐additive formulations are therefore preferred when electrical conductivity is the primary requirement, while binder‐containing systems may be advantageous where mechanical durability, adhesion, and shape fidelity are more critical. These trade‐offs directly influence device‐level performance, including electrode resistance, sensor sensitivity, power delivery, and electrochemical rate capability. The optimal formulation must therefore be selected by jointly considering rheological performance, interflake connectivity, and the electrical requirements of the intended device.

## 4 Applications

Functional MXene inks engineered via ligand grafting, polymer/ionic‐liquid matrices, and hybrid heterostructures combine high electrical conductivity with tunable rheology, strong interfacial adhesion, and compatibility with inkjet, screen, DIW, and EHD printing [155, 156, 157]. These attributes have enabled rapid progress across printed sensors, energy‐storage components, and EMI shielding, while also extending to triboelectric nanogenerators (TENGs), TFTs, antennas, and emerging flexible display elements [29, 34]. This section discusses the recent developments through a print–process–property–performance lens, emphasizing device reliability (cycling stability, drift, hysteresis), environmental robustness (humidity/oxidation tolerance), and manufacturability (pattern fidelity, yield, roll‐to‐roll readiness).

### 4.1 Sensors

Printed sensors represent one of the most extensively explored application domains for MXene‐based functional inks, leveraging their high conductivity, tunable surface chemistry, and mechanical flexibility to detect a broad spectrum of stimuli ranging from gases and biomolecules to pressure, strain, and humidity [158, 159]. Different types of MXene ink have been employed to fabricate sensors through a variety of printing techniques as shown in Table 5. One representative example is the use of additive‐free MXene ink for constructing an NFC sensing system via DIW. As shown in Figure 7a, the structural architecture of a flexible sensing module integrates MXene patterns directly printed onto a substrate with a gel electrolyte and humidity‐sensitive layer to achieve multifunctional detection. The device operation is further detailed in Figure 7b, where the DIW‐printed MXene sensors are seamlessly coupled with a wireless platform, demonstrated through an NFC‐enabled module capable of simultaneously monitoring temperature and humidity in a battery‐free configuration. The sensor performance is illustrated in Figure 7c–f, which depict temperature‐dependent resistance responses, fast humidity sensing and recovery cycles, resistance‐humidity calibration curves, and long‐term stability across repeated humidity steps. Collectively, Figure 7 highlights three defining advantages of functional MXene inks: (i) the ability to be patterned into high‐resolution, mechanically compliant electrodes via DIW; (ii) tunable surface chemistry that enables selective and stable environmental sensing; and (iii) intrinsic compatibility with flexible substrates and integrated wireless electronics. Within the broader field of printable electronics, the example in Figure 7 demonstrates how ink formulation and DIW printing fidelity directly translate into practical device‐level attributes, including rapid response, reversibility, and reliable system integration paving the way for wearable and IoT‐oriented sensing platforms. Notably, compared to inkjet printing, which excels in high‐resolution droplet deposition but suffers from nozzle clogging and limited solids loading, and screen printing, which enables scalable patterning at the expense of resolution, DIW provides a valuable balance by accommodating higher‐viscosity MXene inks without additives while retaining fine feature control and structural integrity.

**TABLE 5:** Summary of reported MXene‐based printed sensors.

Printing method	Year	MXene type	Target	Limit of detection	Response time	Sensitivity	Working range	Ink formulation/notes	Ref.
Extrusion printing	2022	Ti_3_C_2_T*_x_*	Temperature	NR	NR	≈ 0.066% per °C	20°C–55°C	Additive‐free MXene ink; room‐temperature printing	[94]
Extrusion printing	2022	Ti_3_C_2_T*_x_*	Humidity	NR	≈25 s	NR	20%–80% RH	Additive‐free MXene ink; room‐temperature printing	[94]
Screen printing	2023	Ti_3_C_2_T*_x_*	Ethylene (C2H4) gas	0.084 ppm (84 ppb)	NR	1.16% response at 1 ppm (RF shift)	NR	Additive‐free MXene ink; all‐MXene printed RF resonator	[160]
Screen printing	2022	Ti_3_C_2_T*_x_*	Pressure (piezoresistive)	NR	≈130 ms	NR	Up to 30 kPa	MXene + xanthan gum hybrid functional ink	[115]
Screen printing	2023	Ti_3_C_2_T*_x_*	Pressure (piezoresistive)	≈1 Pa	≈70/81 ms (resp./rec.)	≈771–1435 kPa^−1^	0–100 kPa	MXene‐functionalized, multilayer porous structure; breathable textile	[161]
DIW (3D Printing)	2022	Ti_3_C_2_T*_x_*	Pressure/pulse (triboelectric)	9 Pa	≈80 ms	≈6.03 kPa^−1^	NR	MXene‐based triboelectric ink; self‐powered system	[162]
DIW	2024	Ti_3_C_2_T*_x_* + rGO composite	Pressure (piezoresistive)	Range starts at ∼0.028 kPa	NR	≈166.96 kPa^−1^	≈0.028–100 kPa	Ultralow concentration MXene/rGO functional ink	[163]
DIW	2023	Ti_3_C_2_T*_x_*	Mechanosensing (touch/strain)	NR	0.46 s	Guage factor: 6.57	0‐1%	Additive‐free MXene sediment ink; disposable paper sensors	[164]
Transfer printing	2024	Ti_3_C_2_T*_x_* (PEI/AgNO_3_ cross‐linked)	Humidity	NR	≈6.9 s (resp.)/11.9 s (rec.)	Capacitance response up to ≈820% @ 97% RH	to 97% RH (upper bound reported)	Functionalized MXene ink with PEI/AgNO_3_ interlayer cross‐linking	[165]
Microcontact printing	2016	Ti_3_C_2_T*_x_*	Dopamine; neural activity (FET)	100 e^−9^	NR (fast AP detection)	NR	100 e^−9^ to 50 e^−6^	MXene micropatterns via printing; FET biosensor	[166]
EHD printing	2024	Ti_3_C_2_T*_x_*	Aflatoxin B_1_	5.689 ppb	NR	NR	0.7‐20 ppb	Functional EHD‐printed MXene FET channel ink	[119]
EHD printing	2024	Ti_3_C_2_T*_x_* + ionic liquid hydrogel	Strain (frost‐resistant)	NR	73 ms	Gauge Factor = 4.17	1‐600%	Functional MXene‐*co*‐ionic liquid hydrogel ink	[120]

**FIGURE 7:**
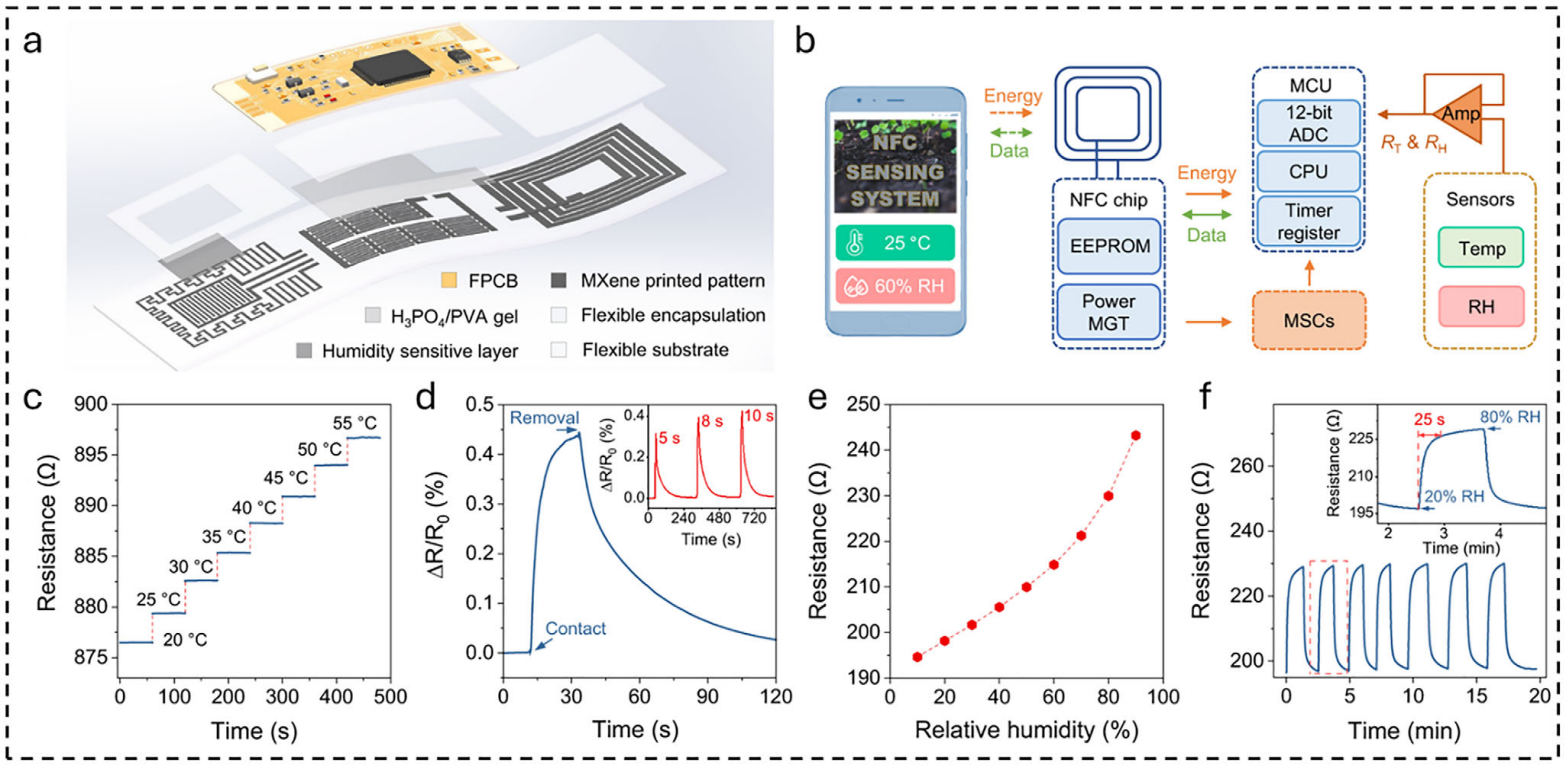
MXene‐based printed multifunctional sensor platform. (a) Schematic of the flexible sensing module comprising a flexible printed circuit board (FPCB), MXene printed electrodes, H_3_PO_4_/PVA gel electrolyte, humidity‐sensitive layer, encapsulation, and substrate. (b) Block diagram of the NFC‐enabled wireless sensing system integrating temperature (T) and relative humidity (RH) sensors with energy harvesting, signal conditioning, and data transmission units. (c) Temperature‐dependent resistance response of the MXene printed sensor measured between 20°C and 55 °C. (d) Humidity sensing dynamics showing relative resistance change (Δ*R*/*R*_0_) under contact/removal cycles (inset: fast response/recovery times of 8–10 s). (e) Steady‐state resistance as a function of relative humidity from 20% to 100% RH. (f) Dynamic response of the printed humidity sensor across repeated RH steps (20%–80%), demonstrating reversible and stable signal behavior (inset: 25 s response at 20%–80% RH). Reproduced with permission [94]. Copyright 2022, Nature.

In another work, MXene printing was demonstrated using EHD deposition, highlighting its potential for both biosensing and soft hydrogel electronics. Figure 8a shows an EHD‐printed field‐effect transistor (FET) biosensor designed for the detection of aflatoxin B1 (AFB1), a potent food‐borne toxin. The sensing mechanism involves sample extraction in 80% methanol from contaminated food, followed by immobilization of anti‐AFB1 antibodies and *L*‐cysteine linkers onto the printed MXene channel. The binding of AFB1 modulates the channel conductivity, which is captured by a source measure unit and processed for quantitative readout, demonstrating the high selectivity and sensitivity of MXene‐based printed FETs for real‐time food safety monitoring. Figure 8b illustrates the fabrication of a multifunctional hydrogel matrix composed of acrylamide (AM), gelatin, glycerol, and ionic liquid, integrated with MXene flakes and crosslinked via MBA and a photoinitiator. Through EHD printing and UV curing, a hierarchical network is created via intermolecular hydrogen bonding and gelation‐induced triple helix structures. Subsequent cooling reinforces the matrix with additional non‐covalent interactions, yielding enhanced elasticity, mechanical integrity, and print fidelity. The incorporation of MXene flakes provides both electronic functionality and structural resilience, making the system attractive for soft electronics, stretchable sensors, and biomedical devices.

**FIGURE 8:**
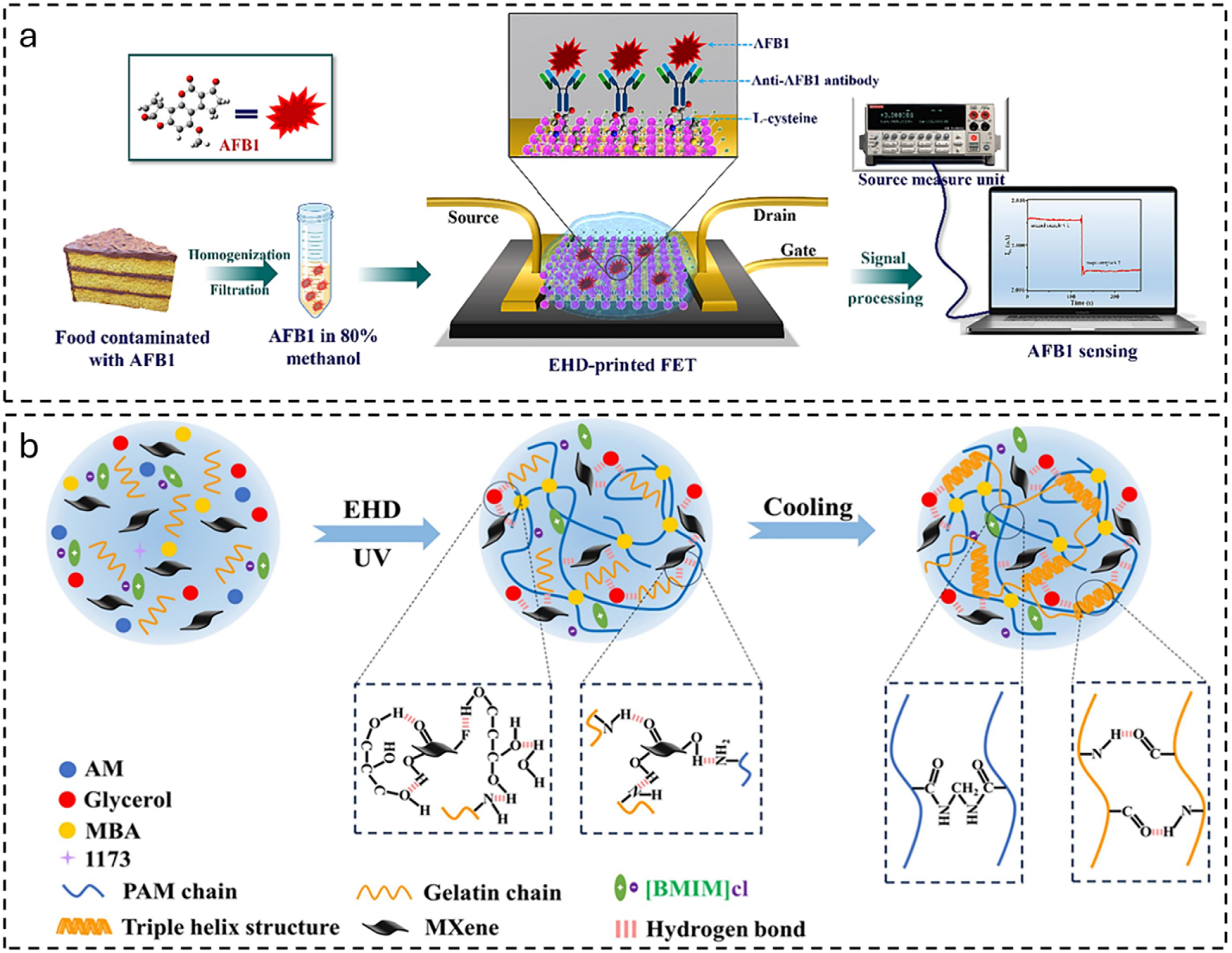
(a) Schematic illustration of an EHD‐printed MXene‐based field‐effect transistor (FET) for sensing aflatoxin B1 (AFB1) in contaminated food. AFB1 is first extracted using methanol from homogenized food samples, followed by detection using an EHD‐printed FET functionalized with anti‐AFB1 antibodies and *L‐*cysteine linkers. The interaction between AFB1 and antibodies modulates the FET signal, which is processed for AFB1 quantification using a source measure unit. Reproduced with permission [119]. Copyright 2024, Elsevier. (b) Fabrication mechanism of MXene‐integrated multifunctional hydrogel via EHD printing and UV curing. Initial formulation includes acrylamide (AM), glycerol, gelatin, crosslinker MBA, photoinitiator, and ionic liquid. Upon UV exposure and EHD printing, crosslinking and hydrogen bonding occur among PAM and gelatin chains, forming triple helix structures. Subsequent cooling further enhances the mechanical integrity via strengthened hydrogen bonds and polymer network formation. Reproduced with permission [120]. Copyright 2024, Elsevier.

Despite these advances, the inks employed in Figure 8 were prepared from ultrathin MXene flakes. Such formulations often suffer from poor rheological control and limited oxidation stability, as shorter flakes present more active surface sites that accelerate degradation. Thus, while EHD‐printed MXene devices achieve high resolution and multifunctionality, their long‐term durability and scalability remain constrained unless complemented by functionalization strategies or protective chemistries. Compared with DIW and screen printing, both of which tolerate higher‐viscosity inks and allow incorporation of functional additives, EHD offers unmatched feature resolution but places stricter demands on ink stability and dispersion control, underscoring the trade‐offs between printing precision and material robustness.

Another representative example of MXene‐based sensing platforms is shown in Figure 9, which contrasts a simple spray‐deposition strategy with a chemically modified functional ink approach. In Figure 9a–d, a piezoresistive sensor is produced by spraying MXene flakes onto PDMS reinforced with air‐laid paper and assembling the layered structure into a flexible pressure‐sensing device. The sensor exhibits pressure‐dependent *I–V* characteristics (Figure 9b), stepwise current changes under varying pressures (Figure 9c), and repeatable cyclic responses during loading/unloading tests (Figure 9d). While this straightforward spray‐coating method demonstrates rapid fabrication and proof‐of‐concept functionality, the reliance on unfunctionalized MXene flakes results in suboptimal rheology and limited oxidation stability, as the exposed active sites of shorter flakes accelerate degradation.

**FIGURE 9:**
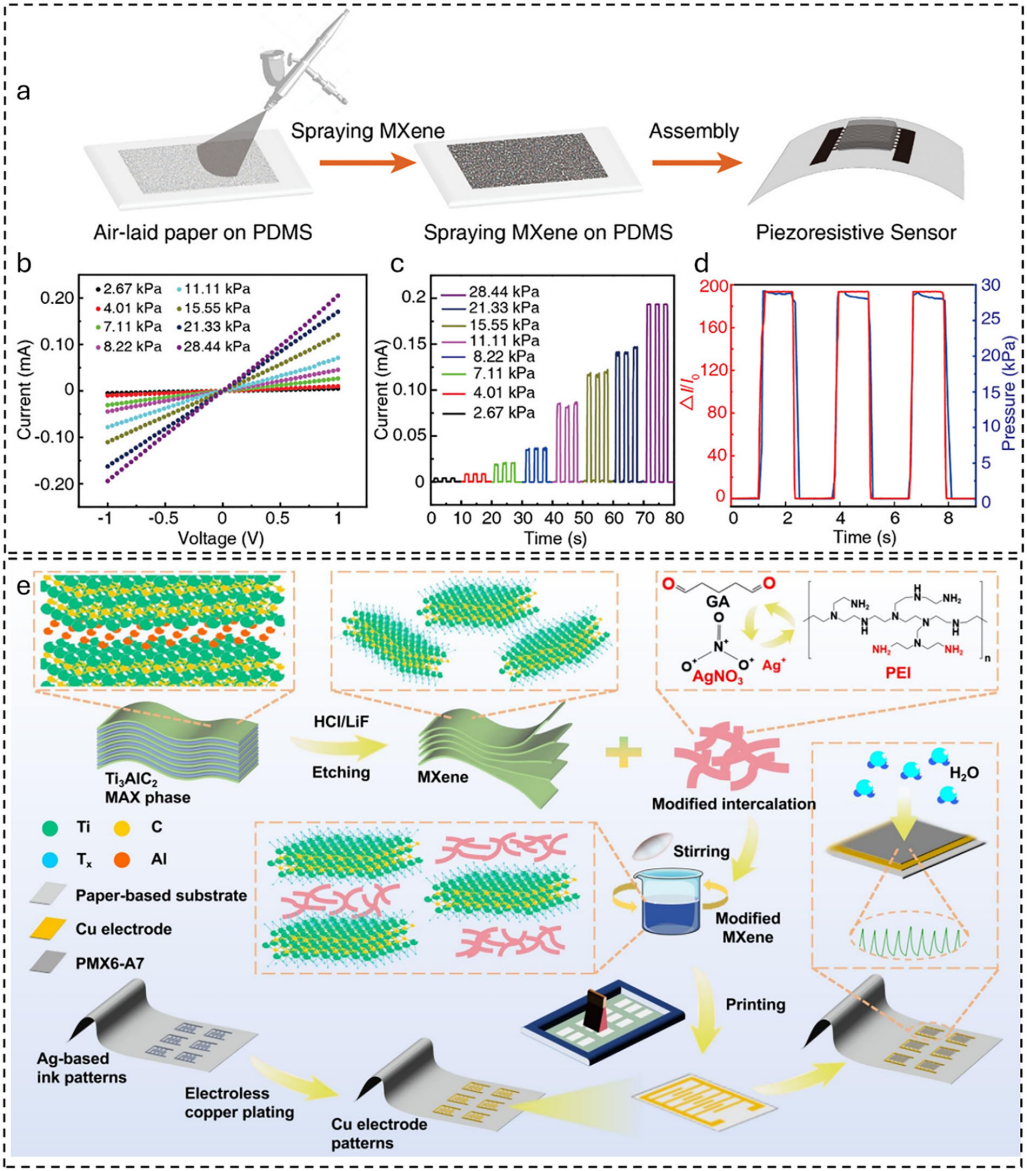
MXene‐based printed and functionalized pressure/humidity sensors. (a) Fabrication schematic of a piezoresistive sensor prepared by spraying MXene flakes onto PDMS supported with air‐laid paper, followed by assembly into a flexible pressure‐sensing device. (b) *I–V* curves of the MXene/PDMS piezoresistive sensor under applied pressures ranging from 2.67 to 28.44 kPa, showing pressure‐dependent current responses. (c) Dynamic current output of the sensor at different applied pressures, demonstrating stepwise and stable signal changes. (d) Cyclic response of the device under repeated loading/unloading at 21.33 kPa, highlighting excellent repeatability and stability. Reproduced with permission [115]. Copyright 2022, Wiley. (e) Schematic of surface‐modified MXene functional inks for humidity sensing: Ti_3_AlC_2_ MAX phase is etched to produce MXene nanosheets, followed by AgNO_3_/PEI‐assisted intercalation and stabilization. The resulting modified MXene is processed into printable inks and integrated with Ag/Cu electrode patterns on paper substrates for humidity‐responsive devices. Reproduced with permission [165]. Copyright 2024, Elsevier.

Breathable, flexible, and highly sensitive pressure sensors are pivotal for wearable electronics (body‐motion monitoring, human–machine interfaces), yet polymer‐backed devices often suffer from low air/vapor permeability and mechanical mismatch that reduce comfort and limit use. Figure 10 addresses these constraints with a fabric‐based, printable MXene architecture. Figure 10a outlines LiF/HCl etching of MAX precursors and mechanical delamination to yield MXene nanosheets suitable for ink formulation; Figure 10b shows representative dispersions and micrographs confirming printability. In Figure 10c, nonwoven fabric serves as both the electrode layer (screen‐printed interdigitated MXene) and the sensing layer (coated with an MXene/AgNW network) using a scalable printing route. The resulting multilayer, porous stack is air/water‐permeable, skin‐conformable, and supports multimodal sensing. Figure 10d schematically depicts signal transduction under external mechanical stimuli, where the MXene and AgNW layers, coupled to interdigitated electrodes, convert pressure into a stable relative current change (Δ*I/I*_0_).

**FIGURE 10:**
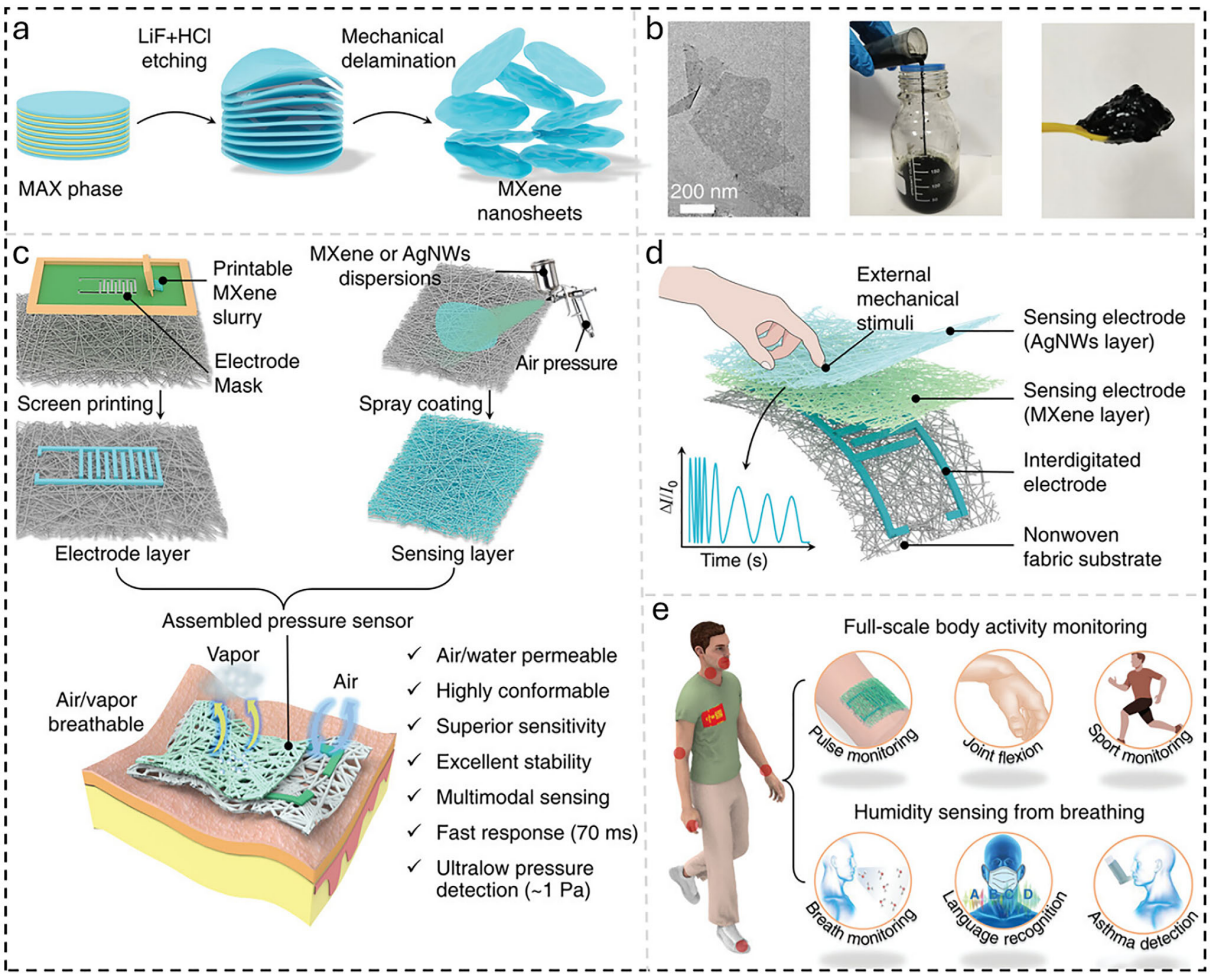
Breathable MXene‐based pressure sensor fabricated on nonwoven fabric. (a,b) Preparation of printable MXene nanosheets. (c) Fabrication of the porous, air‐permeable sensor via screen printing (electrode) and spray coating (sensing layer). (d) Device structure and pressure‐sensing mechanism. (e) Applications in full‐scale body activity and respiratory monitoring, demonstrating high sensitivity, fast response, and multimodal detection. Reproduced with permission [161]. Copyright 2023, Wiley.

Performance and use cases are summarized in Figure 10e. Benefiting from the porous textile architecture, the sensor achieves high sensitivity (≈771–1435 kPa^−^^1^) over a wide 0–100 kPa range, fast 70/81 ms response/recovery, and a low detection limit of ∼1 Pa. The device captures full‐scale human motion‐from subtle pulse beats to large‐amplitude walking/running‐with excellent cycling stability and puncture resistance. Notably, the sensing layer is also humidity‐responsive, enabling breath monitoring and even spoken‐word recognition when embedded in a face mask. Collectively, Figure 10 demonstrates how pairing MXene‐based functional inks with breathable nonwoven fabrics overcomes comfort and permeability limitations of polymer‐based sensors while delivering high sensitivity and fast dynamics. Remaining considerations for translation include long‐term oxidation stability of MXene on textiles and maintaining robust ink‐fiber adhesion under perspiration and repeated laundering‐issues that can be mitigated through surface functionalization, protective overcoats, and roll‐to‐roll compatible printing/curing workflows. Compared to inkjet or DIW, which achieve higher resolution but face challenges with ink viscosity, nozzle clogging, and throughput, screen printing provides a robust, rapid, and low‐cost method ideally suited for scalable manufacturing, where device scalability, durability, and ease of application outweigh the need for fine micro‐patterning.

The application of MXene‐based functional inks in plant‐wearable electronics demonstrates how printed circuits can extend beyond traditional wearable devices into agriculture and food monitoring. Fabrication of flexible ethylene‐detecting antennas using screen‐printed MXene circuits subsequently modified with Pd nanoparticles are outlined in Figure 11a. The process involves sequential plasma cleaning, screen printing of MXene circuits, film modification with Pd nanoparticles for selective ethylene sensing, and final encapsulation with PDMS for mechanical robustness. Morphological characterization (Figure 11b,c) confirms uniform film coverage and well‐defined printed features, while statistical analysis of printed linewidths (Figure 11d) shows minimal variation (<*W*> ≈ 2.5%), underscoring the reproducibility of the printing process.

**FIGURE 11:**
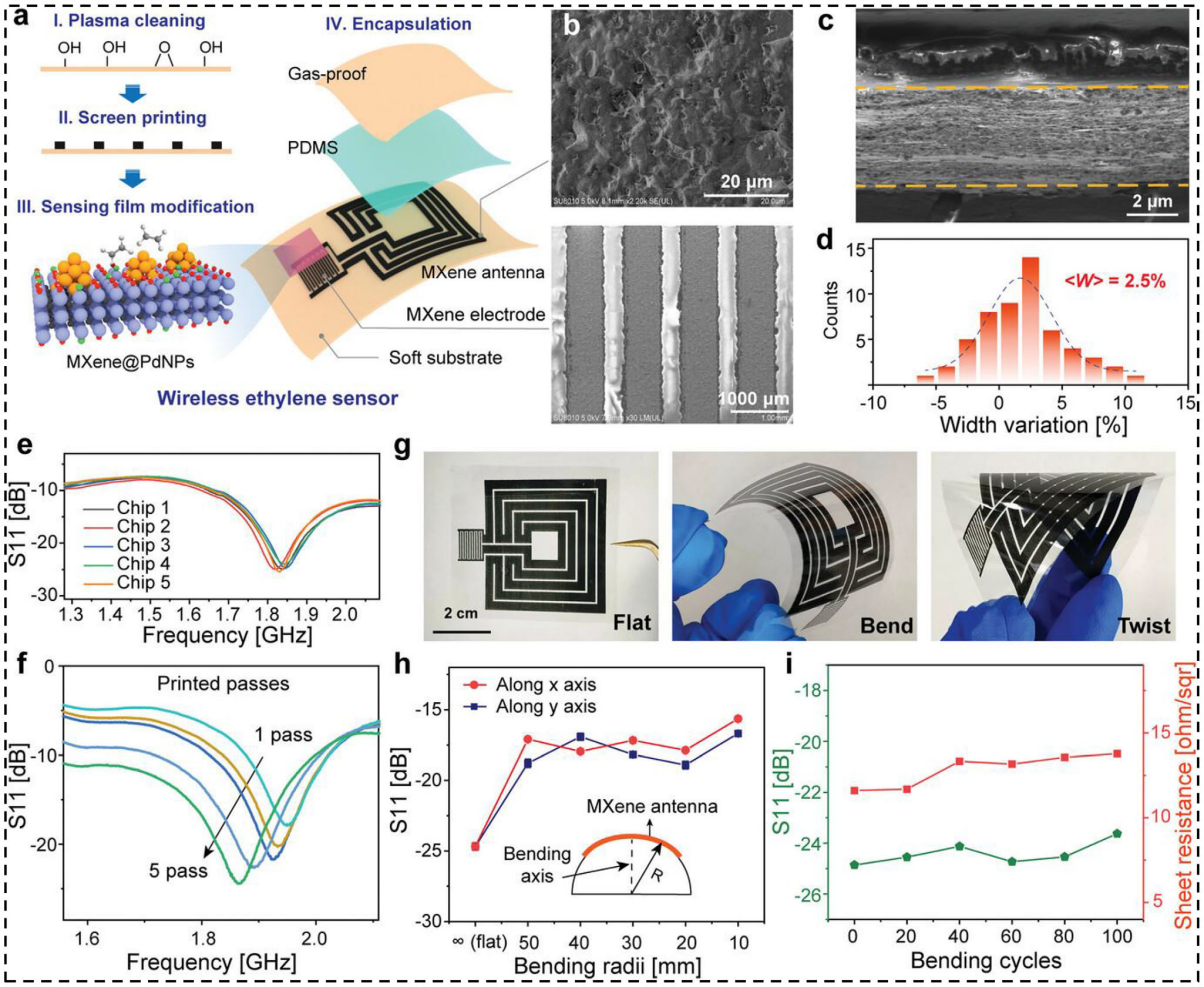
Printed MXene inks for plant wearable/wireless ethylene sensors. (a) Fabrication steps including plasma cleaning, circuit printing, PdNP modification, and PDMS encapsulation. (b,c) Morphological and cross‐sectional images of printed films. (d) Linewidth variation showing high reproducibility (<*W*> ≈ 2.5%). (e,f) Antenna performance with consistent S11 curves across devices and tunability with multiple print passes. (g) Flexibility under flat, bent, and twisted states. (h,i) Stable wireless response and sheet resistance under bending radii and repeated cycling. Reproduced with permission [160]. Copyright 2023, Wiley.

Electrical and wireless performance metrics are presented in Figure 11e,f, where S_11_ curves demonstrate high uniformity across multiple devices and tunability of antenna resonance with increasing print passes. The printed antennas retain stable structural characteristics under mechanical deformation, as shown in Figure 11g, with flat, bent, and twisted configurations. Further bending tests (Figure 11h) reveal consistent antenna performance along different axes and radii, while cycling experiments (Figure 11i) confirm stable sheet resistance and S_11_ responses after repeated mechanical stress. This example illustrates how functional MXene inks, enhanced with Pd nanoparticles, can be processed through scalable screen printing to produce mechanically robust, wireless‐responsive devices. By combining high conductivity, conformability, and chemical selectivity, these antennas enable low‐cost, flexible platforms for real‐time ethylene monitoring in agricultural and supply‐chain contexts. Importantly, the reproducibility and stability demonstrated here point to the readiness of MXene inks for integration into large‐scale wireless IoT sensing systems, bridging the gap between material‐level functionalization and practical device deployment.

Direct ink writing (DIW) of binder‐free MXene sediment inks provides a rapid, low‐cost route to disposable paper mechanosensors, with patterns written directly onto cellulose substrates for customizable geometries and flexible form factors. The test configuration (Figure 12a) probes strain‐dependent resistance changes. Figure 12b shows that sensors printed from sediment‐rich inks exhibit larger Δ*R/R*_0_ than those from multilayer‐only dispersions, reflecting superior electromechanical transduction. Stronger bending responses at 8 mm deflection (Figure 12c) arise from the hierarchical conductive microstructure in s‐MXene inks, interwoven nanosheets, nanoflakes, and nanoparticles, which intensifies pathway disruption under deformation (Figure 12d). The devices combine fast dynamics (∼0.31/0.46 s response/recovery; Figure 12e) with robust durability (Figure 12f) and good reproducibility across multiple prints (Figure 12g). Taken together, the results show that DIW‐processed, additive‐free MXene sediment inks can deliver eco‐friendly, scalable, and high‐performance paper sensors.

**FIGURE 12:**
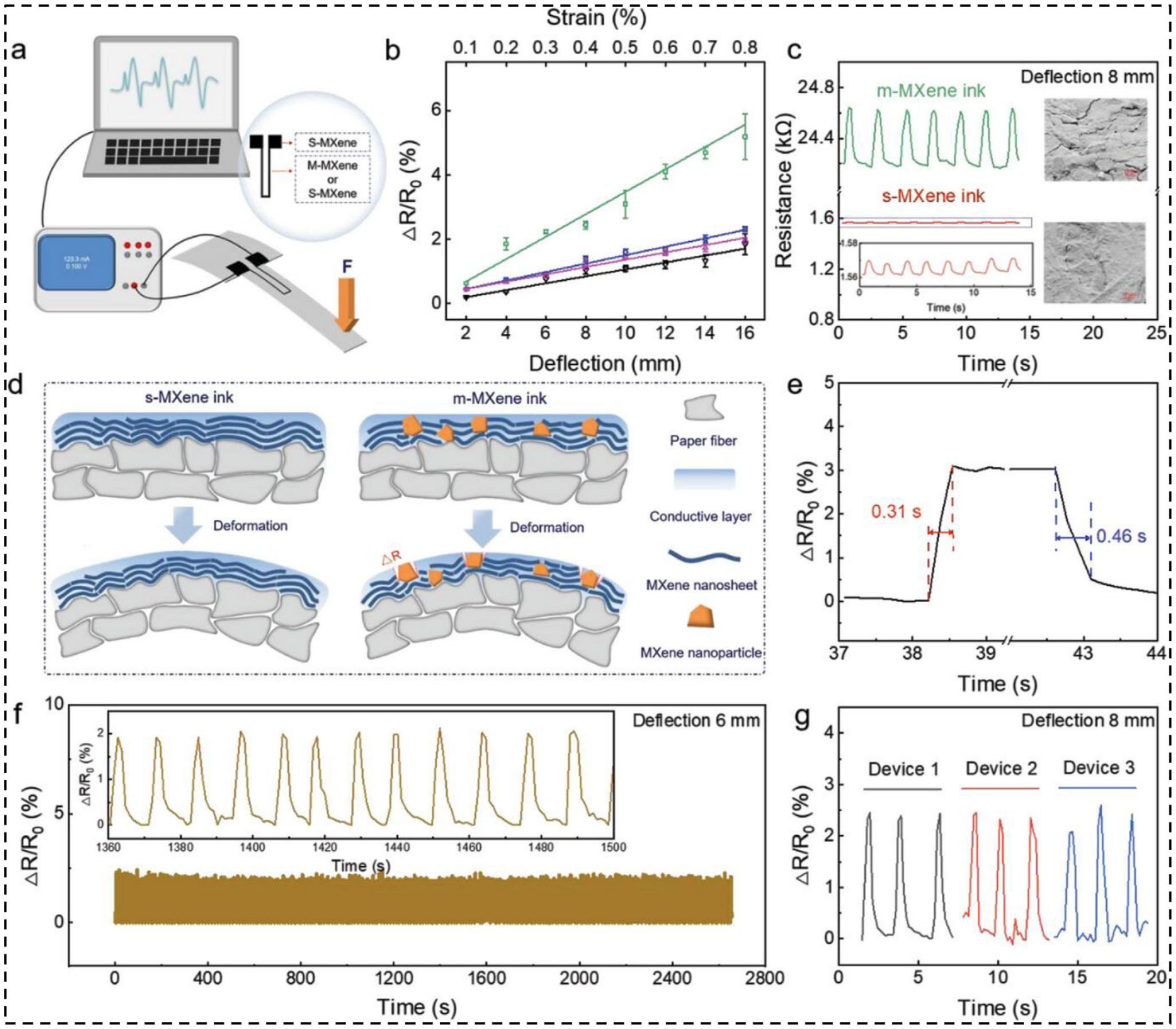
MXene‐based paper mechanosensors fabricated via direct ink writing (DIW). (a) Strain‐sensing measurement setup. (b) Δ*R*/*R*_0_ versus deflection for sensors from different MXene inks. (c) Repeated bending response for multilayer MXene (m‐MXene) versus sediment MXene (s‐MXene) inks. (d) Mechanism: s‐MXene forms a hierarchical network of nanosheets/nanoflakes/nanoparticles that amplifies resistance modulation under strain. (e) Fast response/recovery. (f) Long‐term cycling stability over thousands of cycles. (g) Device‐to‐device reproducibility. Reproduced with permission [164]. Copyright 2023, Wiley.

The exceptional electrical conductivity and high surface area of MXenes make them suitable for printed sensor applications, including wearable strain sensors, gas sensors, and biosensors. For instance, MXene‐based strain sensors exhibit high sensitivity and rapid response times, attributed to the material's conductive pathways that respond linearly to mechanical deformations. Additionally, MXenes can be functionalized to selectively interact with target molecules, enhancing the specificity and sensitivity of printed chemical and biological sensors. For example, an electrochemical biosensor for vitamin D detection was developed by using antibody‐functionalized Ti_3_C_2_T_*x*_ MXene, demonstrating high sensitivity, specificity, and suitability for point‐of‐care testing. The Ti_3_C_2_T*_x_* nanosheets were first amine‐functionalized via electrostatic modification with polyethylenimine (PEI), introducing abundant reactive groups for covalent conjugation of anti‐vitamin D antibodies through glutaraldehyde chemistry. This functional architecture enabled efficient antibody immobilization while preserving biological activity, resulting in an ultralow detection limit and a broad dynamic range. The approach highlights the utility of PEI‐functionalized MXene for enabling stable, solution‐processable inks with improved chemical tunability and potential for integration into printed sensors, transistors, and bioelectronic devices [167].

### 4.2 Energy Storage Devices

MXene inks have been explored for energy storage applications, particularly in supercapacitors and batteries [49, 139, 168, 169]. Their high conductivity and redox‐active sites enable efficient charge transfer, while the layered structure supports ion intercalation [170]. Printed MXene supercapacitors have demonstrated impressive specific capacitance and cycling stability, marking a step forward in the development of flexible, portable energy storage systems [171, 172]. Additionally, hybrid formulations incorporating MXenes and other nanomaterials, such as carbon nanotubes or conductive polymers, have been proposed to further enhance energy storage capacities [173]. MXene‐based inks have recently been developed to serve not only as electrodes but also as multifunctional components that simplify device integration, as shown in Table 6. One representative example is reported by Zheng et al., where multitasking aqueous MXene inks were formulated to act simultaneously as electrodes, interconnectors, current collectors, and conductive binders. As shown in Figure 13a, the additive‐free aqueous MXene ink served as a universal building block, while Figure 13b highlights hybrid formulations (MXene‐LTO and MXene‐LFP inks) tailored for lithium‐ion microbatteries (LIMBs). Direct screen printing of these inks on flexible substrates (Figure 13c) enabled both microsupercapacitors (MX‐MSCs) and MX‐LIMBs, achieving ultrahigh areal capacitance of ≈1.1 F/cm^2^ and serial voltages up to 60 V for MSCs, and ≈154 µWh/cm^2^ areal energy density for LIMBs. The performance benchmarking in Figure 13d further confirms their superiority compared with other reported flexible energy storage devices. The key takeaway is that a single additive‐free MXene ink can be screen‐printed into multiple device roles, greatly simplifying fabrication and supporting the vision of monolithic, multifunctional energy storage systems.

**TABLE 6:** Summary of reported MXene‐based printed energy storage devices.

Printing method	Year	MXene type	Device type/structure	Key performance metrics	Measurement conditions	Cycling/environmental stability	Ink formulation/notes	Ref
Stamp printing	2018	Ti_3_C_2_T_*x*_	All‐MXene solid‐state micro‐supercapacitors on paper	61 mF/cm^2^ at 25 µA/cm^2^ and 50 mF/cm^2^ at 800 µA/cm^2^;	PVA/H_2_SO_4_ gel electrolyte; voltage window 0–0.6 V; CV tested at 5–200 mV/s;	93.7% capacitance after long‐term cycling	Additive‐free aqueous Ti_3_C_2_T_*x*_ ink	[92]
Extrusion Printing	2019	Ti_3_C_2_T_*x*_	Direct‐printed micro‐supercapacitor	Areal capacitance from 3.5 to 43 mF/cm^2^ for <*N*> = 1 to <*N*> = 5; energy density up to 0.32 µW h/cm^2^ at 11.4 µW/cm^2^;	Solid‐state MSCs coated with 3 m H_2_SO_4_/PVA gel electrolyte; voltage window 0–0.5 V;	97% during long‐term cycling	Additive‐free aqueous Ti_3_C_2_T_*x*_ ink in DI water; concentration about 36 mg/mL;	[22]
Inkjet Printing	2019	Ti_3_C_2_T_*x*_	All‐MXene printed micro‐supercapacitors on AlO_x_‐coated PET	Areal capacitance: 1.3 mF/cm^2^ at <*N*> = 2; 12 mF/cm^2^ at <*N*> = 25; volumetric capacitance up to 562 F/cm^3^;	Solid‐state MSCs tested with 3 M H_2_SO_4_/PVA gel electrolyte; voltage window 0–0.5 V;	100% during long‐term cycling	Additive‐free Ti_3_C_2_T_*x*_ inks in NMP, DMSO, DMF, or ethanol; NMP/DMF/DMSO concentrations up to 12.5 mg/mL, ethanol about 0.7 mg/mL;	[22]
3D printing / DIW	2020	Ti_3_C_2_T_*x*_	3D micro‐supercapacitor	Areal capacitance at 2 mV/s: 168.1 mF/cm^2^ for MSC‐1, 685.4 mF/cm^2^ for MSC‐5, and 1035 mF/cm^2^ for MSC‐10;	Two‐electrode configuration; voltage window 0–0.6 V; CV scan rates 2–1000 mV/s;	Electrochemical response remained stable under bending cycles	MXene dispersion 10 mg/mL, concentrated using super absorbent polymer beads to 290 mg/mL, ∼ 28.9 wt.%;	[89]
Screen printing	2019	N‐doped Ti_3_C_2_T_*x*_	all‐MXene interdigital micro‐supercapacitor	Areal capacitance: 70.1 mF/cm^2^ at 10 mV/s and 62.5 mF/cm^2^ at 100 mV/s;	CV tested at 10–100 mV/s; voltage window from figure: 0–0.6 V;	92% capacitance retention after 7000 cycles at 5 mA/cm^2^;	MXene‐N active material + Super P + LA132 binder in DI water;	[28]
3D Extrusion printing	2019	N‐doped Ti_3_C_2_T_*x*_	3D‐printed symmetric SCs using layer‐by‐layer printing	Higher areal capacitance than pristine Ti_3_C_2_, Ti_2_CN, and graphene at matched scan rates;	Electrochemical tests performed in 3 m H_2_SO_4_ electrolyte; CV/GCD/EIS used; key capacitance reported at 10 mV/s	96.2% capacitance retention after 5000 cycles;	MXene‐N + activated carbon (AC) + carbon nanotubes (CNTs) + graphene oxide (GO);	[28]
Screen printing	2021	Ti_3_C_2_T_*x*_	Microsupercapacitors printed mainly on A4 paper	MX‐MSC‐10L: areal capacitance 1108 mF/cm^2^ ≈ 1.1 F/cm^2^; areal energy density 13.8 µWh/cm^2^; power density 2.5 mW/cm^2^	CV measured from 2 to 50 mV/ s; GCD shown at 0.4 mA/cm^2^; cycling tested at 3 mA/cm^2^;	No capacitance fade after 10 000 cycles	Additive‐free aqueous H‐MXene ink; concentration about 10 wt.%, ∼100 mg/mL, ∼3.4 vol%;	[46]
Stepwise screen printing	2021	Ti_3_C_2_T_*x*_ with LTO/LFP hybrid inks	Lithium‐ion microbattery	Areal capacity 88 µAh/cm^2^ at 20 µA/cm^2^; 46 µAh/cm^2^ at 100 µA/cm^2^; areal energy density 154 µWh/cm^2^;	Ionogel electrolyte: LiTFSI/P14TFSI/PVDF‐HFP; GCD at 20–100 µA/cm^2^; redox peaks at 2 and 1.7 V;	82% capacity retention after 1000 cycles	Aqueous MXene‐based LTO and LFP inks: 70 wt.% LTO or LFP + 30 wt.% MXene;	[46]
DIW	2023	PEDOT: PSS/MXene/ethylene glycol gel composite	Programmable micro‐supercapacitor	Areal capacitance 242.4 mF/cm^2^ and volumetric capacitance 5.54 F/cm^3^ at 1 mA/cm^2^; retained 193.6 mF/cm^2^ and 4.43 F/cm^3^ at 10 mA/cm^2^	CV tested mainly from 10–200 mV/s; GCD tested from 1–10 mA/cm^2^; device voltage window 0–0.8 V;	83% capacity retention after 6000 cycles at 10 mA/cm^2^; ∼ 100% capacity retention under bending (180°)	PME1: 16 mL commercial PEDOT: PSS PH1000, 4 mL ethylene glycol, and 42 mg MXene; PME2: 69 mg MXene; PME3: 104 mg MXene;	[40]
Screen printing	2024	Multilayer Ti_3_C_2_T_*x*_ MXene	Flexible planar asymmetric micro‐supercapacitor	Energy density ≈117.6 mWh/cm^3^; power density ≈ 1285.7 mW/cm^3^	CV tested from 5–100 mV/s; GCD tested from 0.4–1.2 mA;	≈72% retention after 1000 cycles	Multilayer Ti_3_C_2_T_*x*_ MXene + activated carbon + ethyl cellulose/terpineol binder‐solvent mixture;	[77]
Printed interdigitated architecture	2024	Cysteine‐functionalized Ti_3_C_2_T_*x*_ MXene	Printed microsupercapacitor	Areal energy density: 1.4–1.43 µWh/cm^2^ at 170.6 µW/cm^2^; 0.74 µWh/cm^2^ at 93.1 µW/cm^2^;	CV shown from 10–100 mV/s; GCD measured at low current densities; key capacitance reported at 1 µA/cm^2^	97% capacitance retention after 10 000 cycles	MX‐C dispersion concentration up to 23.1 mg/mL; viscosity 1.1 Pa s at 1 s^−^^1^	[174]
EHD printing	2024	ADOPA‐functionalized Ti_3_C_2_T_*x*_ MXene	Symmetric on‐chip high‐density micro‐supercapacitor	Volumetric capacitance: 2013 F cm^−^^3^; areal capacitance: 402.7 mF cm^−^^2^;	CV tested from 1–150 mV s^−^^1^; single‐device voltage window 0–0.6 V;	>95% capacitance retention after 10,000 cycles	ADS‐MXene flakes + CMC hybrid solvent solution, weight ratio 65:35;	[172]

**FIGURE 13:**
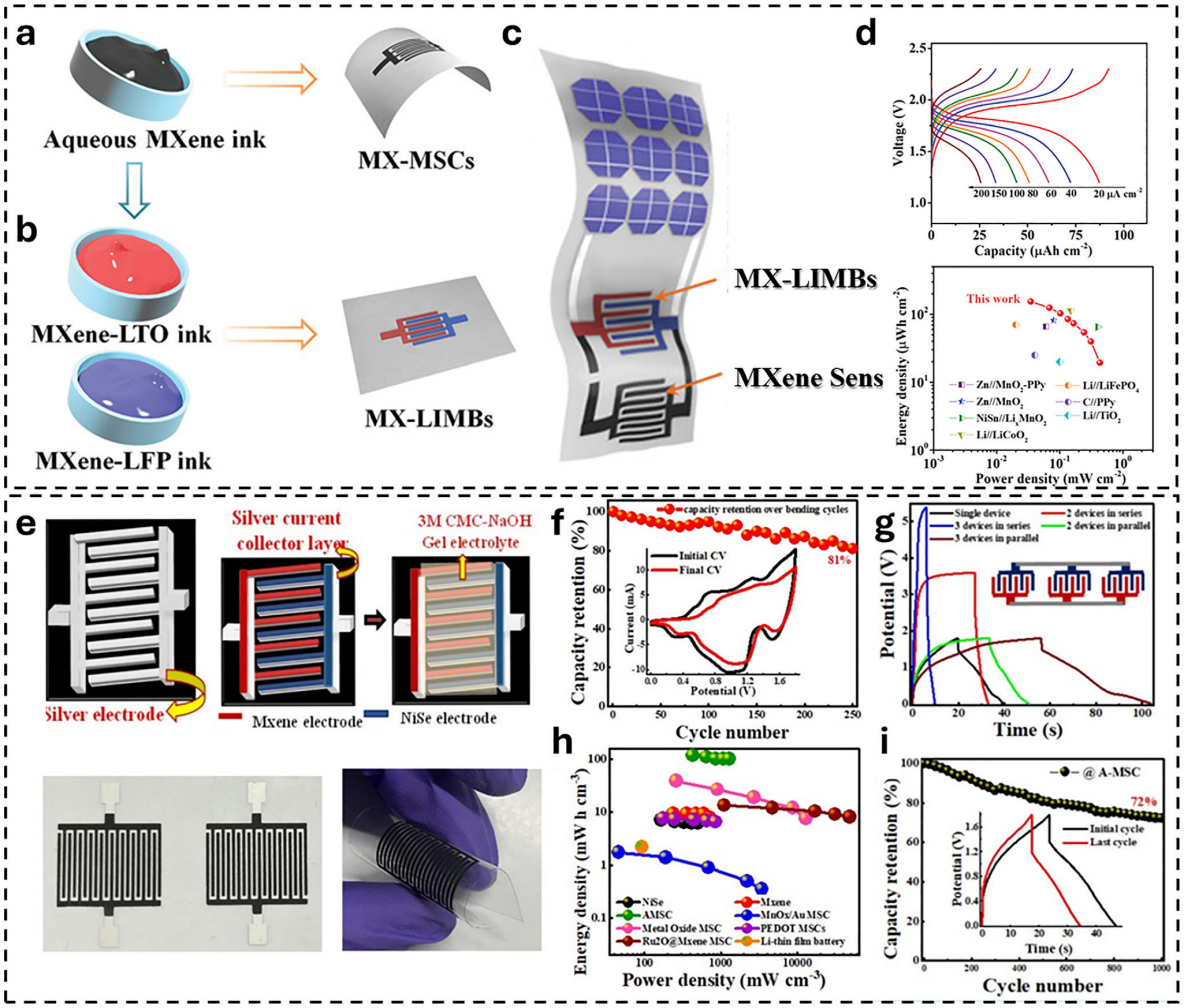
MXene‐based printable inks for integrated energy storage. (a–d) Multitasking aqueous MXene inks screen‐printed as electrodes, interconnectors, and binders for microsupercapacitors (MX‐MSCs) and lithium‐ion microbatteries (MX‐LIMBs). Reproduced with permission [46]. Copyright 2021, Wiley. (e–i) Screen‐printed asymmetric micro‐supercapacitors (A‐MSCs) combining Ti_3_C_2_T_*x*_ MXene as the negative electrode and NiSe as the positive electrode for high‐energy‐density, flexible devices. Reproduced with permission [77]. Copyright 2024, Elsevier.

In a complementary study, Dey et al. demonstrated how hybridization can further enhance MXene‐based storage devices. Their approach involved a mask‐assisted screen‐printing process to fabricate interdigitated electrodes with Ti_3_C_2_T_*x*_ MXene as the negative electrode and NiSe nanoparticles as the positive electrode. The design, illustrated in Figure 13e, produced flexible asymmetric micro‐supercapacitors (A‐MSCs) that retained excellent structural integrity under bending. Electrochemical results in Figure 13f showed stable capacitance under deformation, while Figure 13g demonstrated tunable outputs through series and parallel connections. The Ragone plot in Figure 13h highlights impressive metrics, with an energy density of ≈117.6 mWh/cm^3^ and power density of ≈1285.7 mW/cm^3^, and long‐term testing in Figure 13i confirmed ≈72% retention after 1000 cycles in aqueous electrolyte. By combining MXene's rapid ion transport with NiSe's high surface reactivity, these screen‐printed hybrid inks successfully address the energy‐power trade‐off that often limits micro‐supercapacitors.

Altogether, Figure 13a–i emphasizes two complementary strategies: multitasking aqueous MXene inks that simplify device architecture by consolidating multiple roles, and hybrid MXene//NiSe inks that push the performance boundaries of micro‐supercapacitors. Both rely on screen printing as the enabling deposition method, underscoring its scalability and compatibility with flexible substrates. In terms of ink formulation, additive‐free MXene inks are highly attractive for their multifunctionality and simplicity, but they still face challenges of oxidative instability and limited rheological control. Hybrid inks, while more complex, provide improved stability and enhanced energy storage performance. Thus, ink design and printing choice directly determine the balance between scalability, durability, and electrochemical performance in MXene‐based energy storage devices.

Efforts to expand the functionality of MXene inks have included both elemental doping and molecular surface engineering. One approach, presented by Li et al. (2019), developed nitrogen‐doped MXene inks through a combined assembly‐annealing route. As shown in Figure 14a, the resulting MXene‐N (nitrogen‐doped) inks can be directly deployed in screen printing for planar devices and extrusion printing for 3D architecture, providing a versatile toolkit for different device geometries. Practical demonstrations in Figure 14b show flexible printed electrodes conforming to skin and wearable substrates, while electrochemical benchmarking in Figure 14c reveals significantly enhanced areal capacitance compared with Ti_3_C_2_, Ti_2_CN, and exfoliated graphene, especially at high scan rates. This highlights how ink formulation through N‐doping directly improves electrochemical performance, while maintaining printability across multiple techniques. A complementary strategy is illustrated in Figure 14d–f, where Wang et al. report cysteine‐stabilized MXene inks designed to overcome oxidative degradation. In this method, Ti_3_C_2_ MXene flakes are functionalized with cysteine molecules, which prevent surface oxidation while simultaneously allowing metal‐selective binding and improved aqueous stability (Figure 14d). The resulting dispersions remain stable for extended periods and can be processed into printable inks for microsupercapacitors. Device schematics and photographs (Figure 14e) demonstrate how the inks are integrated into printed interdigitated architecture, and the electrochemical data (Figure 14f) confirm improved capacitance retention compared with unmodified MXenes, even at high current densities.

**FIGURE 14:**
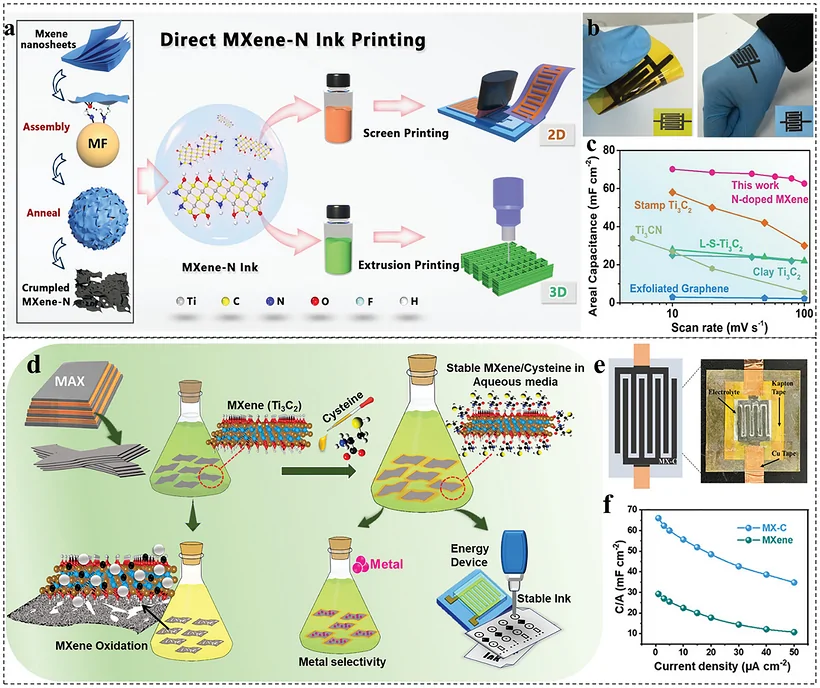
Functional MXene inks for advanced energy storage. (a) Schematic illustration of N‐doped MXene ink preparation via assembly and annealing, followed by direct use in screen printing (2D patterns) and extrusion printing (3D architectures). (b) Photographs of flexible printed devices using the MXene‐N inks, demonstrating conformal adhesion to curved/wearable substrates. (c) Electrochemical benchmarking showing the enhanced areal capacitance of N‐doped MXene inks compared to pristine Ti_3_C_2_, Ti_2_CN, and graphene‐based references across different scan rates. Reproduced with permission [28]. Copyright 2019, Wiley. (d) Process route for cysteine‐modified MXene inks, where molecular functionalization suppresses oxidation, stabilizes aqueous dispersions, and provides metal selectivity. (e) Device schematic and optical image of printed interdigitated microsupercapacitors fabricated with cysteine‐stabilized MXene inks. (f) Electrochemical performance comparison, highlighting improved capacitance retention of MX‐C inks over unmodified MXenes at higher current densities. Reproduced with permission [174]. Copyright 2024, Wiley.

In summary, Figure 14a–f highlights that rational ink design, via elemental doping or surface functionalization, can markedly improve the stability, performance, and versatility of MXene‐based printable devices. Importantly, both studies underscore that the success of screen and extrusion printing is inseparable from ink chemistry, since rheological control and oxidation resistance dictate whether MXene inks can be reliably translated into high‐performance, flexible energy storage systems.

Further related to ligand‐functionalized MXene inks, another study shows how catechol chemistry and EHD printing can be translated into practical micro‐energy devices, as shown in Figure 15. In Figure 15a, Ti_3_C_2_T_*x*_ was functionalized with ADOPA to yield solvent‐compatible ADS‐MXene, which was then formulated as a hybrid‐solvent, lightly crosslinkable ink to suppress oxidation, limit restacking, and tune viscosity and surface tension for printing. The same ink was patterned onto silicon, glass, PI, and PET, and interdigitated electrodes were stacked with a gel electrolyte to form fingertip‐scale micro‐supercapacitors, confirming substrate breadth and room‐temperature processing (Figure 15b). EHD printing served as the enabling method: operation in the cone‐jet regime decoupled feature size from nozzle diameter, stabilized line edges at low flow, and minimized coffee‐ring defects, which preserved percolation, lowered ESR, and supported high areal capacitance. Benchmarking against recent reports using the radar plot in Figure 15 shows that the combination of ligand‐functionalized MXene ink and EHD printing provides improved dispersion and oxidation stability, sustained conductivity retention during aging, fine patterning resolution, and high areal device density. These results demonstrate the rationale for integrating molecular‐level surface stabilization with precision printing to produce dense, low‐resistance micro‐supercapacitor architectures on diverse substrates. EHD printing is particularly suitable for fabricating critical fine‐feature patterns, while future implementation of multi‐nozzle systems could improve throughput and support larger‐area manufacturing.

**FIGURE 15:**
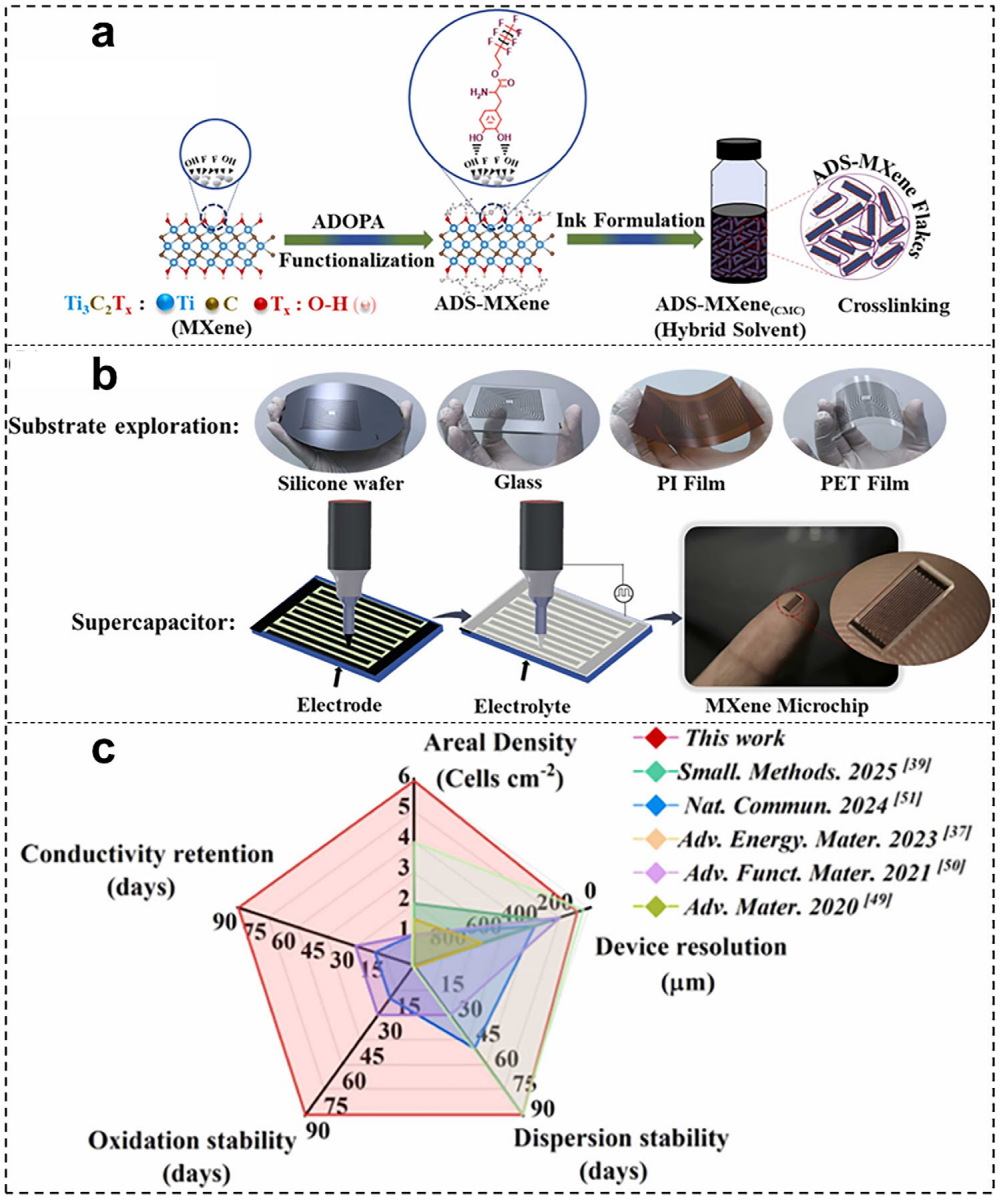
Functional MXene inks for printed micro‐energy storage. (a) Surface engineering and ink design: Ti_3_C_2_T_*x*_ is functionalized with ADOPA ligands to yield solvent‐compatible MXene (ADS‐MXene), formulated into a hybrid‐solvent ink and cross‐linked to produce stable, printable flakes. (b) Printing and device demonstrations: the ink is patterned on diverse substrates silicon wafer, glass, PI film, and PET film to form interdigitated electrodes and gel electrolyte layers, enabling compact MXene micro‐supercapacitors (inset: fingertip‐scale microchip). (c) Literature‐normalized benchmarking (radar plot) comparing this system with recent reports on areal cell density (cells/cm^2^), device resolution (µm), dispersion stability (days), oxidation stability (days), and conductivity retention (days). Reproduced with permission [172]. Copyright 2026, Elsevier. Note: Embeded references refer to ref. 172's bibliography.

Despite these advances, the stability metrics reported in Figure 15 remain limited to days‐to‐months timescales, which are substantially shorter than the year‐scale service lifetimes expected for industrial electronic devices. Ink shelf life, nanosheet oxidation resistance, and operational device reliability also represent distinct stability requirements and should be evaluated separately. Practical translation therefore requires standardized accelerated‐aging protocols, including elevated‐temperature and humidity storage, thermal cycling, prolonged electrical operation, mechanical fatigue, and application‐specific exposure to light, oxygen, moisture, and chemical environments. Conditions such as 85°C and 85% relative humidity can provide useful comparative evidence of degradation resistance [30]; however, accelerated‐aging results must be correlated with real‐time storage and operational testing before reliable service‐life predictions can be established. Future studies should therefore report both material‐level stability and device‐level performance retention under clearly defined aging conditions.

### 4.3 Electromagnetic Interference (EMI) Shielding

With the proliferation of electronic devices, EMI shielding has become a critical requirement for maintaining signal integrity and protecting sensitive electronic components [175]. MXene inks offer promising solutions for printed EMI shielding due to their metallic conductivity and tunable thickness. Recent studies show that printed MXene films can achieve high EMI shielding effectiveness even at low thicknesses, outperforming traditional metal‐based solutions as shown in Table 7. Moreover, the flexibility and processability of MXene inks provide opportunities for lightweight, conformable EMI shields compatible with wearable electronics.

**TABLE 7:** Summary of reported MXene‐based printed EMI shielding.

Printing method	Year	MXene Type	Structure/application	Key performance metrics	Working band/bandwidth	Ink formulation/notes	Ref
DIW/direct ink writing	2021	Ti_3_C_2_T_*x*_	3D porous MXene for tunable EMI shielding	Conductivity up to 5323 S/m; tunable EMI SE 25–80 dB;	X‐band	MXene/AlOOH hybrid inks;	[113]
Screen printing	2022	Ti_3_C_2_T_*x*_	Multifunctional coatings for EMI shielding,	Avg. EMI SE 40.1 dB in X‐band; Film conductivity around 480 S/cm;	8.2–12.4 GHz, X‐band	Aqueous MXene/xanthan gum hybrid ink;	[115]
Spray coating	2024	Graphene@MXene composite	Flexible polymer‐supported THz shielding film	MXene/graphene layered structure, of 1.63 µm thickness, achieved shielding effectiveness (*SE*) of 47 dB	Terahertz band	Spray‐deposited composite ink containing graphene, MXene, and PVP	[125]
DIW/direct ink writing	2023	Ti_3_C_2_T_*x*_/sodium alginate	Aerogel mesh variant MS	Conductivity 2.85 × 10^3^ S/m; tunable EMI SE 45–100 dB;	X‐band	Aqueous MXene/sodium alginate ink; ion‐reinforced printed network	[176]
DIW/direct ink writing	2023	Ti_3_C_2_T_*x*_/sodium alginate/Ca^2^^+^ reinforced	Aerogel mesh variant MSCa0.2	EMI shielding effectiveness ≈64.2 dB	X‐band	Calcium‐ion reinforcement strengthens printed MXene/alginate architecture	[176]
Screen printing	2023	ADOPA‐functionalized Ti_3_C_2_T_*x*_	Fabric‐based EMI‐shielding layer	*SE*_T_ > 80 dB; absorption‐dominant shielding	X‐band (8–12 GHz)	Catechol/perfluoroalkyl ligand design makes MXene solvent‐processable	[30]
Coating/printable organic‐solvent deposition	2023	ADOPA‐functionalized Ti_3_C_2_T_*x*_	Coated EMI‐shielding film on arbitrary substrate	*SE*_T_ > 80 dB; absorption‐dominant shielding	X‐band	Universal‐ligand chemistry translated from dispersion to scalable coating/printing	[30]
Coating/solvent evaporation	2023	Janus Ti_3_C_2_T_*x*_ with polystyrene grafts	Large‐area coating on hydrophilic substrate	*SE*_T_ increases with thickness across X‐band; exact maximum NR	X‐band	Additive‐free concentrated organic Janus‐MXene ink	[177]
Coating/solvent evaporation	2023	Janus Ti_3_C_2_T_*x*_ with polystyrene grafts	Large‐area coating on hydrophobic substrate	*SE*_T_ increases with thickness across X‐band	X‐band	Amphiphilic Janus surfaces broaden dispersibility across polar and nonpolar media	[177]
Extrusion printing/additive manufacturing	2022	Ti_3_C_2_T_*x*_/PEDOT: PSS	Printed conductive hydrogel lattice	Electrical conductivity up to 1525.8 S/m at ∼97 wt.% water; EMI‐shielding application demonstrated	X‐band (8–12 GHz)	Hydrogen‐bonded/electrostatically stabilized MXene‐PEDOT: PSS aqueous ink	[178]
Direct ink writing, DIW	2025	Ti_3_C_2_T_*x*_/Fe_3_O_4_/CCMC with AgNW/CCMC	DIW‐printed honeycomb aerogels	EMI shielding about 80 dB at 35% printing fill density	X‐band	Ti_3_C_2_T_*x*_/Fe_3_O_4_/CCMC‐AgNW/CCMC	[179]
Aerosol jet printing,	2025	MXene/Ag‐Ag nanocomposite ink;	Ultrathin 3D‐conformal EMI shielding films on complex substrates	Printed resolution about 10 µm; SSE/t 52 591.4 dB/cm^2^/g at 500 nm thickness with only 0.9 mg mass increase	X‐band	MXene/Ag–Ag hybrid ink; ethylene glycol solvent	[180]

Functional MXene inks offer a route to customizable, multifunctional EMI shielding architectures when combined with suitable binders and rheological control. Zhang et al. developed an aqueous MXene/sodium alginate ink that can be directly processed via direct ink writing (DIW) into aerogel meshes with controlled geometries (Figure 16a). The addition of multivalent ions reinforced the printed networks through hierarchical crosslinking, yielding structures with exceptional mechanical stability in wet environments, maintaining integrity for over 60 days. Importantly, these inks preserved MXene's electrical conductivity, enabling additional functionalities such as Joule heating and tunable EMI shielding, where shielding efficiency could be adjusted by varying mesh design.

**FIGURE 16:**
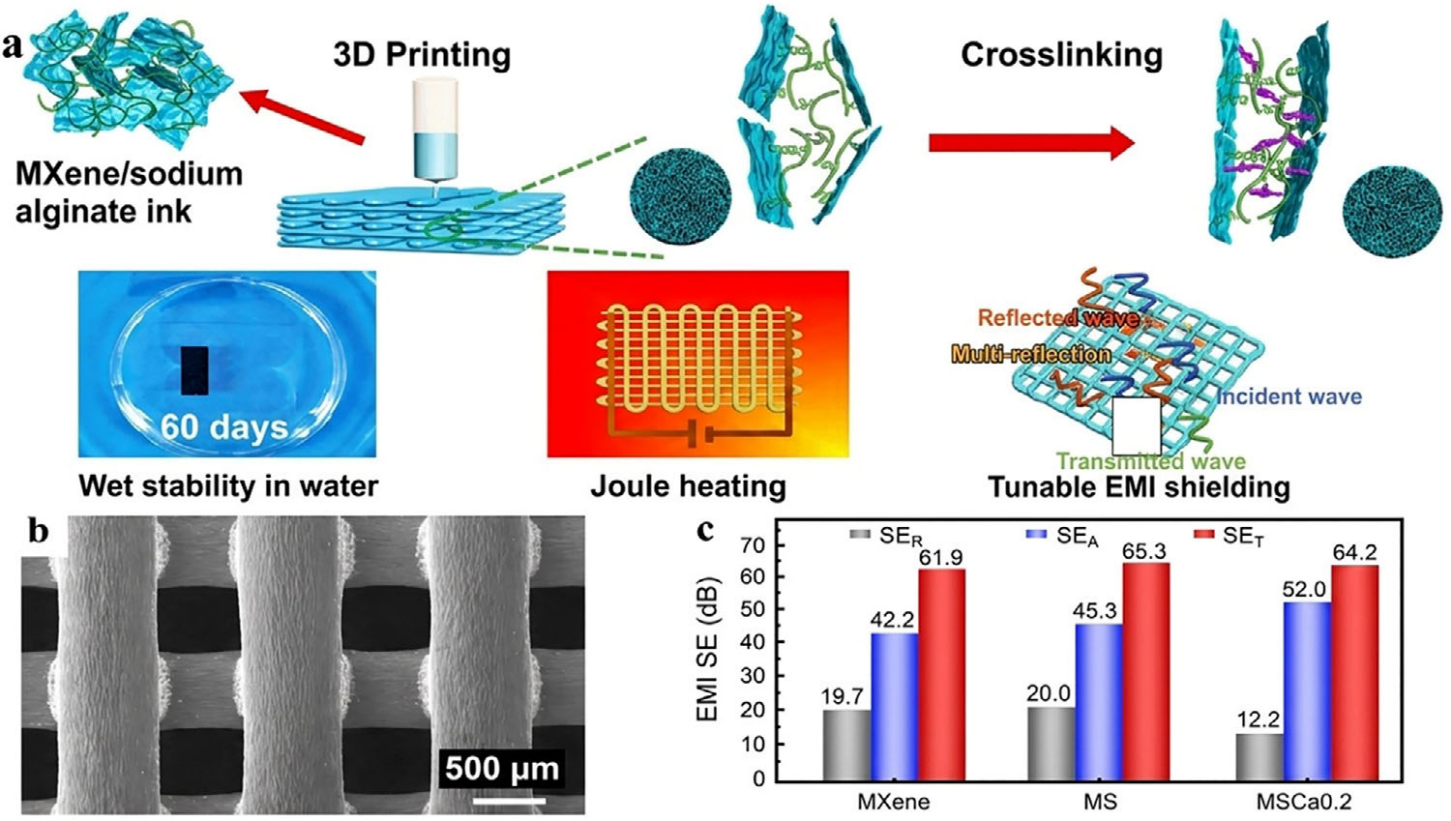
Functional MXene/sodium alginate inks for 3D‐printed EMI shielding aerogels. (a) Schematic of MXene/alginate ink formulation, DIW‐based printing, and ion crosslinking, with demonstrations of long‐term wet stability, Joule heating, and tunable EMI shielding. (b) SEM image of the printed aerogel mesh showing uniform periodic struts. (c) EMI shielding effectiveness (SE) comparison of pristine MXene, MS, and MSCa0.2 aerogels, highlighting tunable absorption‐ and reflection‐dominated contributions. Reproduced with permission [176]. Copyright 2023, Elsevier.

The structural fidelity of the printed aerogels is evident in Figure 16b, where SEM imaging reveals a well‐ordered, periodic mesh critical for reliable performance. EMI shielding performance (Figure 16c) demonstrates efficiencies of 45–65 dB, with tunability achieved by adjusting structure and composition. Overall, this study highlights how functional MXene‐based inks, when combined with DIW, can yield mechanically robust, multifunctional aerogels that couple environmental stability with high EMI shielding performance, an advance that underscores the importance of ink chemistry in achieving reliable, customizable 3D‐printed devices.

A major bottleneck in MXene‐based printable electronics is the limited solvent compatibility of pristine flakes, which hinders their processing into functional inks suitable for coating and scalable printing. To overcome this, Ko et al. (2023) designed functional MXene inks by anchoring catechol‐based ligands (DOPA) onto Ti_3_C_2_T_*x*_ surfaces and further esterifying with perfluorinated alkyl chains to form ADOPA‐modified MXenes (Figure 17a). This molecular design endowed MXenes with dual solubility: hydrophilic catechols maintained surface adhesion, while hydrophobic tails imparted solubility in a wide range of organic solvents. As demonstrated in Figure 17b, the resulting AD1‐MXene dispersions were stable in ethanol, acetone, DMF, DMSO, NMP, and other organic solvents, unlike pristine MXenes, which rapidly precipitated. This modification was also extended to multiple MXene chemistries (Ti_2_CT*_x_*, Nb_2_CT*_x_*, V_2_CT*_x_*, Mo_2_TiC_2_T*_x_*, etc.), with UV–vis spectra confirming stable, uniform dispersions. Such solvent‐compatible MXene inks are highly relevant for screen printing and coating techniques, which often require rheologically tunable inks compatible with organic solvent systems. The printability of these inks was further demonstrated by screen‐printed AD1‐Ti_3_C_2_T*_x_* patterns on fabrics (Figure 17c), with optical and electron microscopy confirming well‐adhered and continuous conductive films at both micro‐ and nanoscale. Importantly, EMI shielding tests highlighted the functional advantage of the modification. As seen in Figure 17d, AD1‐Ti_3_C_2_T*_x_* achieved shielding efficiencies above 80 dB across the X‐band (8–12 GHz), outperforming comparable functional MXenes. Analysis of total shielding (SE_T_), reflection (SE_R_), and absorption (SSE_A_) contributions (Figure 17e) revealed absorption‐dominated behavior, which is particularly desirable for reducing secondary electromagnetic pollution.

**FIGURE 17:**
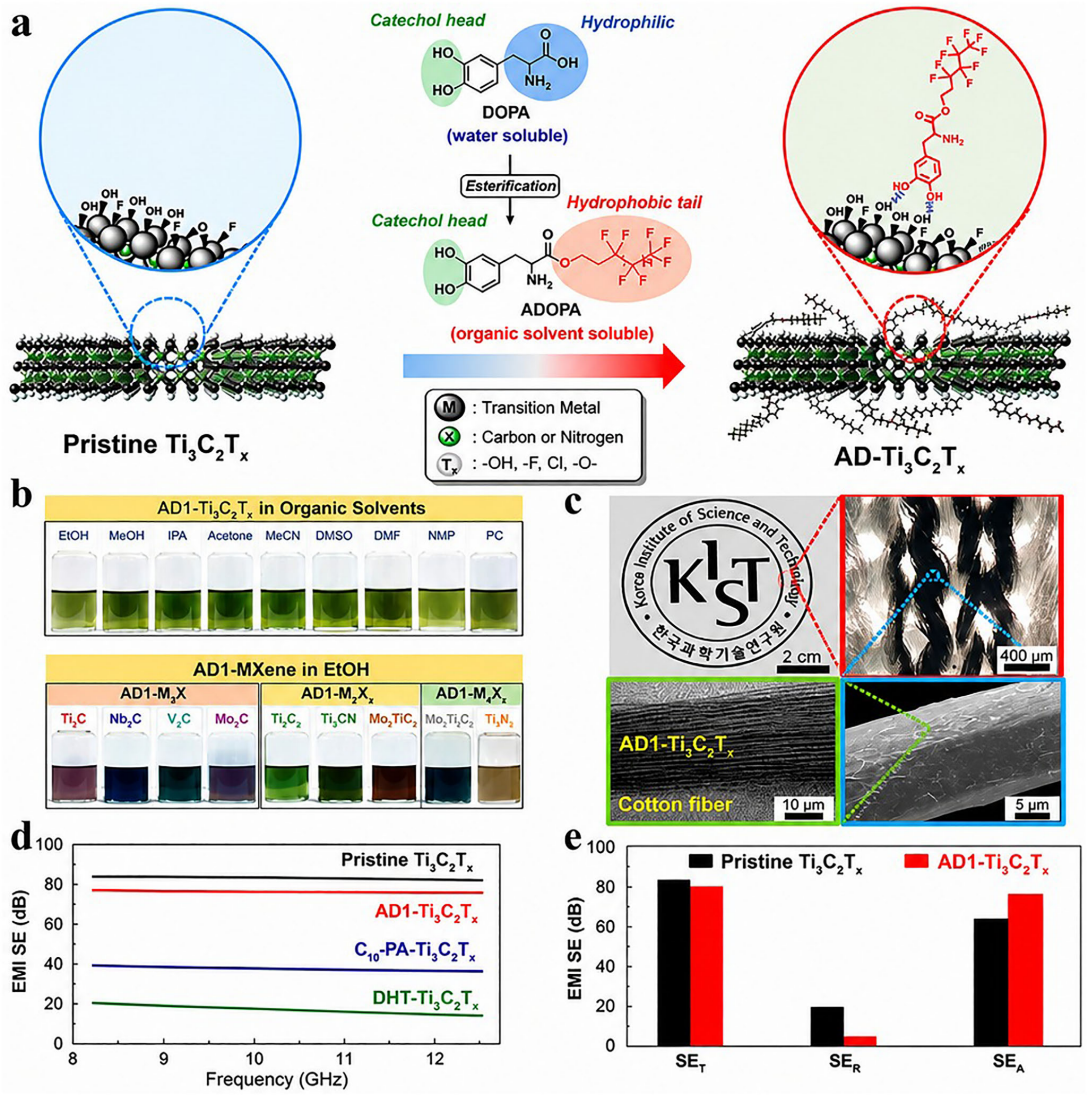
Functionalization strategy for solvent‐processable MXene inks and their EMI shielding applications. (a) Surface modification of Ti_3_C_2_T*_x_* with amphiphilic ADOPA molecules introduces a hydrophobic tail while retaining catechol anchoring groups, yielding MXenes soluble in organic solvents (AD‐Ti_3_C_2_T*_x_*). (b) Photographs of AD1‐Ti_3_C_2_T*_x_* dispersed in a wide range of organic solvents. (c) Digital photograph and optical microscope (red frame), SEM (blue frame), and TEM (green frame) images of fabric screen‐printed with AD1‐Ti_3_C_2_T*_x_* ethanol dispersions. (d) EMI shielding performance comparison of pristine and modified MXenes, and (e) shielding efficiency components (SE_T_, SE_R_, and SE_A_), highlighting the absorption‐dominated behavior of functionalized MXenes. Reproduced with permission [30]. Copyright 2023, ACS.

Collectively, this work illustrates how functional MXene inks, engineered for solvent compatibility, bridge the gap between materials design and scalable printing methods such as screen printing and coating. By enabling stable dispersions in a broad solvent window, this approach ensures that MXene‐based EMI shielding layers can be fabricated with high throughput, structural uniformity, and excellent electromagnetic performance, marking a crucial step toward practical, large‐area printed electronics.

A key limitation of conventional aqueous MXene inks is their narrow substrate/solvent window and susceptibility to oxidation, which constrains printing/coating choices. This study addresses the problem by asymmetrically grafting polystyrene chains onto MXene flakes to create Janus MXene (J‐MXene) with one hydrophilic and one hydrophobic face. As schematized in Figure 18a, the Janus design enables broad dispersibility in polar and non‐polar media and yields additive‐free, concentrated organic inks that cast into uniform, adherent membranes by simple coating/solvent‐evaporation. The amphiphilic surface also slows oxidation kinetics, extending ink shelf life and improving film robustness on both hydrophilic and hydrophobic substrates. Performance tunability follows from controllable film thickness: Figure 18b shows SE_T_ increasing with thickness across the X‐band, enabling application‐specific trade‐offs between weight and shielding. Film photographs in Figure 18c reveal that J‐MXene coatings maintain integrity under handling/solvent contact better than pristine MXene films. Consistently, the 24 h retention of SE_T_ in Figure 18d is substantially higher for J‐MXene, evidencing the chemical/oxidative stability imparted by the Janus surface. By converting MXene into a Janus, solvent‐processable functional ink, this work makes large‐area coating and scalable printing (blade, bar, stencil/screen compatible) far more practical across diverse substrates. The trade‐off is potential conductivity loss from polymer grafts, so ligand loading and post‐treatment must be optimized to balance printability, stability, and electrical performance.

**FIGURE 18:**
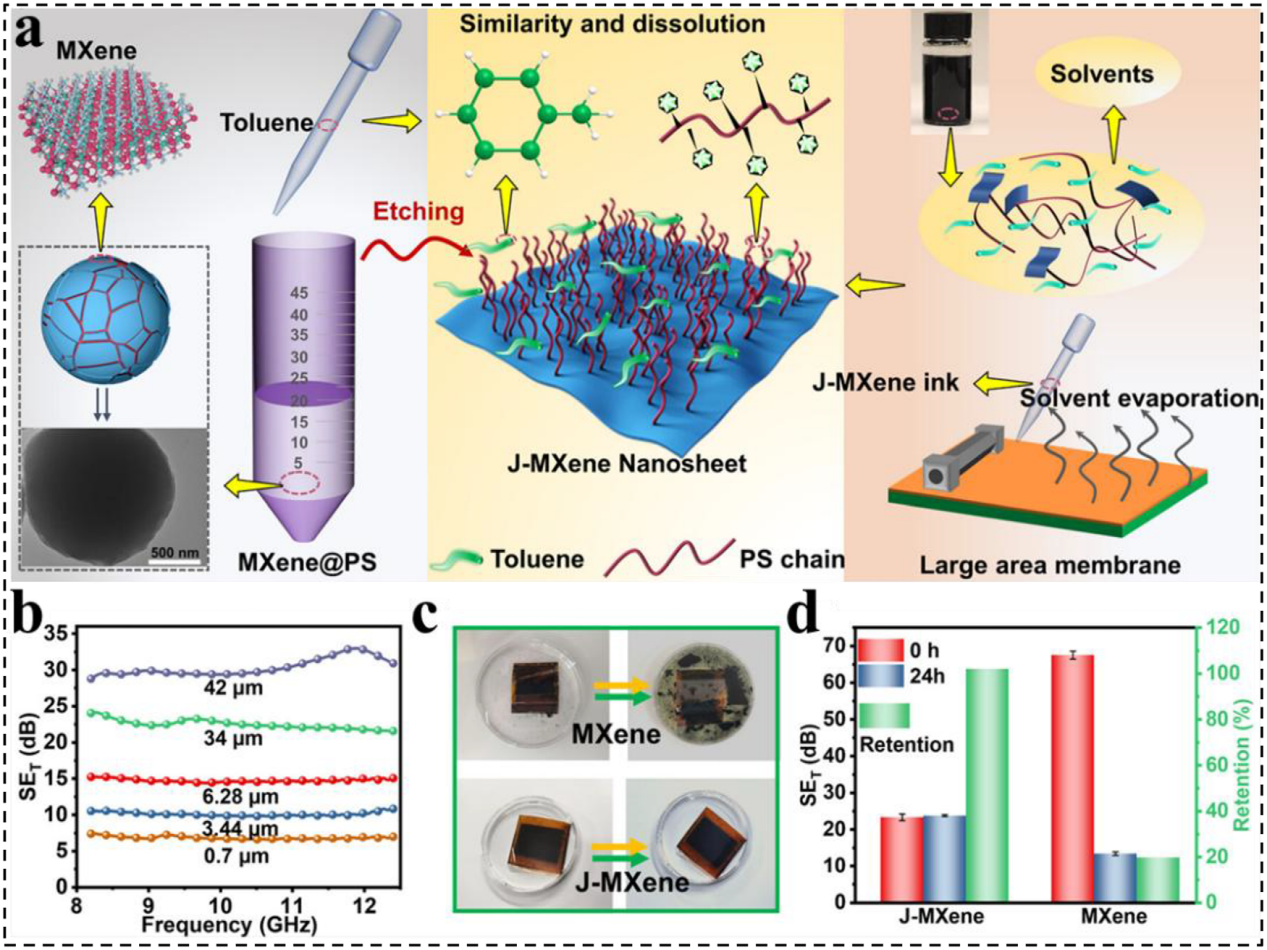
Janus MXene organic inks for large‐area coating and EMI shielding. (a) Schematic of Janus MXene (J‐MXene) preparation by grafting polystyrene chains to Ti_3_C_2_T*_x_*, yielding flakes with asymmetric hydrophilic–hydrophobic surfaces for dispersibility in both polar and nonpolar solvents; the resulting J‐MXene inks enable uniform large‐area coatings through solvent evaporation. (b) EMI shielding performance of J‐MXene films with varying thickness across 8–12 GHz. (c) Optical images comparing MXene and J‐MXene films deposited on substrates, demonstrating uniformity and stability. (d) Stability and retention of EMI shielding efficiency (SE_T_) after 24 h, showing J‐MXene films maintain superior performance compared to pristine MXene coatings. Reproduced with permission [177]. Copyright 2023, ACS.

The integration of MXenes with conductive polymers has enabled the development of functional inks specifically tailored for extrusion‐based 3D printing. In one such work, freeze‐dried PEDOT: PSS powders were combined with Ti_3_C_2_T*_x_* MXene suspensions to produce MXene‐PEDOT: PSS functional inks, stabilized through hydrogen bonding and electrostatic interactions (Figure 19a). Compared with polymer‐only systems, these functional inks exhibited enhanced viscosity and yield stress (Figure 19b,d–f), ensuring the shear‐thinning and viscoelastic balance required for high‐fidelity extrusion printing. With such rheological tuning, the functional inks could be printed into diverse geometries, including microlattices and overhanging architectures (Figure 19c). Rheological analysis further confirmed the inks’ stability during extrusion (Figure 19g), while freeze‐drying transformed the printed hydrogels into mechanically robust, conductive structures (Figure 19h–k). SEM cross‐sections revealed distinct filaments with well‐preserved edges, highlighting how MXene incorporation improves shape fidelity and print resolution.

**FIGURE 19:**
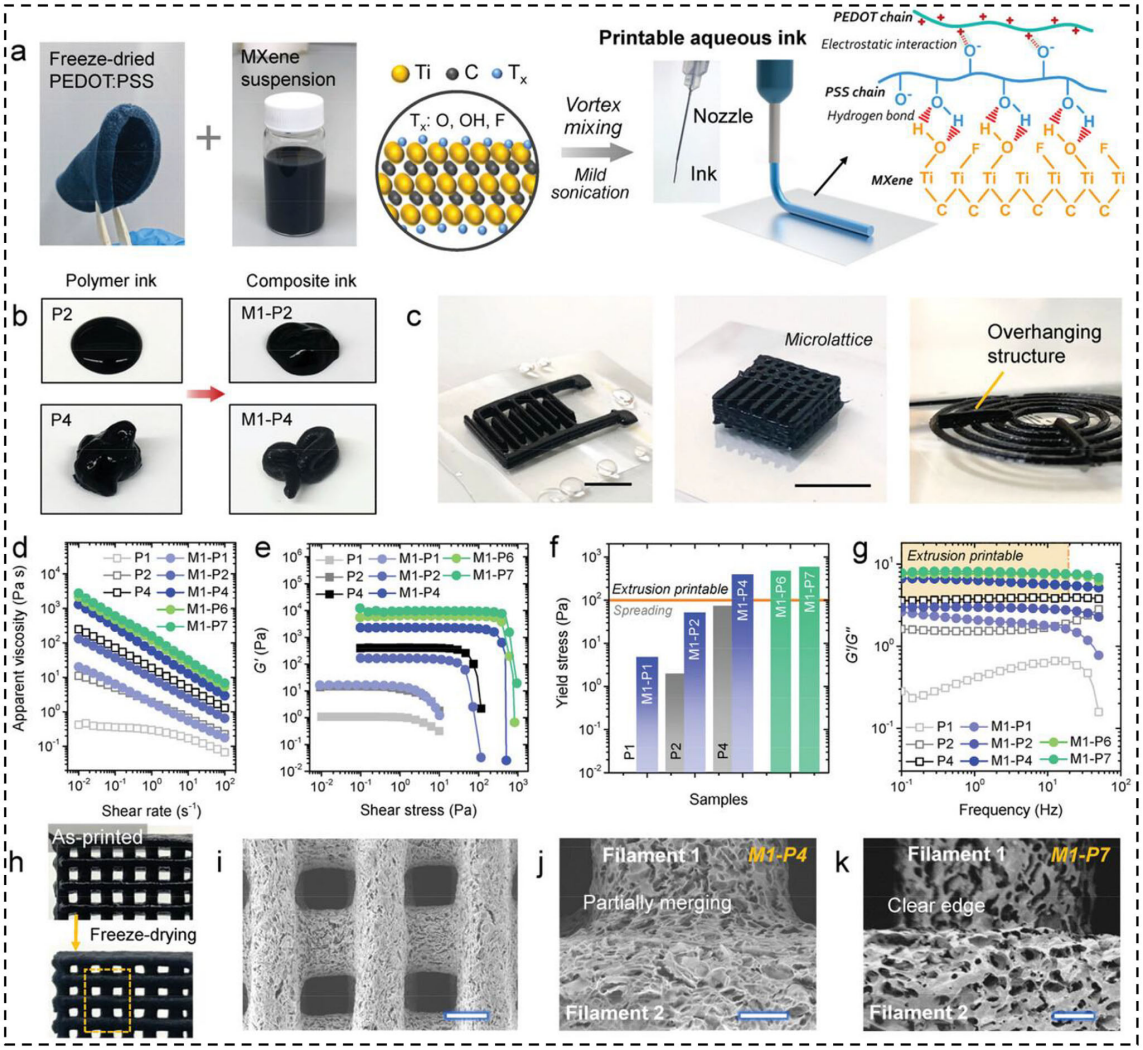
MXene‐PEDOT: PSS functional inks for extrusion printing of conductive hydrogels. (a) Preparation of printable aqueous functional inks via mixing freeze‐dried PEDOT: PSS and Ti_3_C_2_T*_x_* MXene suspensions, forming hydrogen‐bond/electrostatic interactions between MXene flakes and polymer chains. (b) Digital photographs of polymer‐only inks versus MXene‐PEDOT: PSS functional inks showing improved rheological behavior. (c) Printing demonstrations of MXene‐PEDOT: PSS functional inks into lattice and overhanging structures. (d–f) Rheological evaluation, including apparent viscosity, storage modulus, and yield stress, confirming extrusion printability. (g) Frequency‐dependent viscoelastic properties ensuring high shape fidelity. (h–k) Optical and SEM images of as‐printed and freeze‐dried lattices, with cross‐sectional views revealing filament morphology and inter‐filament interfaces. Reproduced with permission [178]. Copyright 2022, Wiley.

The resulting printed hydrogels achieved conductivities up to 1525.8 S/m at ∼97 wt.% water content, while also exhibiting flexibility, stretchability, and fatigue resistance. These multifunctional properties demonstrate the potential of functional MXene inks for producing customizable EMI shielding materials, addressing the dual challenge of electrical performance and design freedom in additive manufacturing. This study exemplifies how rationally engineered functional inks of MXene‐PEDOT: PSS systems directly translate into scalable, extrusion‐printed hydrogels with both high conductivity and tunable architecture, paving the way for next‐generation wearable devices.

### 4.4 Other Emerging Applications

Beyond the core domains of sensing, energy storage, and EMI shielding, MXene‐based functional inks are increasingly being applied to a wide spectrum of emerging technologies. The unique combination of high electrical conductivity, mechanical flexibility, tunable surface chemistry, and solution processability has allowed MXenes to be engineered into printable formulations for unconventional device platforms. For instance, in TENGs, MXene inks serve as efficient charge‐collecting and transfer layers, where their high surface area and conductivity significantly improve energy‐harvesting performance [53, 181]. In TFTs, printed MXene source, drain, and gate contacts enable low‐voltage operation with enhanced carrier mobility, demonstrating their potential for low‐power and flexible electronics [182]. Similarly, photonic and optoelectronic devices have benefited from MXene inks, exploiting their plasmonic resonances and tunable optical properties for dynamic modulation and light–matter interaction [83, 183, 184].

Another growing direction is in printed antennas and wireless components. MXene inks have been used to fabricate near‐field communication (NFC) antennas, taking advantage of their conformability and low electromagnetic loss to produce lightweight, flexible, and high‐performance antenna structures. Taken together, these advances illustrate how functional MXene inks extend beyond traditional electronic domains, driving innovation in energy harvesting, flexible logic circuits, photonics, and wireless communication. This expanding application landscape underscores the versatility of MXene inks and highlights their growing role in shaping next‐generation printed, multifunctional device architectures.

While MXenes are widely recognized for their suitability in TENGs, the work by Q. Yi et al. stands out for its translation of Ti_3_C_2_T_*x*_ into a 3D‐printable functional ink optimized for wearable energy‐harvesting devices (Figure 20). This work represents a milestone in translating MXene functional inks into fully 3D‐printable, wearable, and self‐powered platforms. By tailoring the rheological behavior of Ti_3_C_2_T*_x_* MXene inks to achieve shear‐thinning flow for smooth extrusion, rapid shape recovery for pattern fidelity, and viscosity high enough to prevent structural collapse, the authors enabled the direct printing of robust, flexible electrodes on skin‐like SEBS substrates. This ink engineering preserved flake alignment and conductivity, producing electrodes with low sheet resistance and excellent mechanical durability, critical for repetitive deformation during wearable use.

**FIGURE 20:**
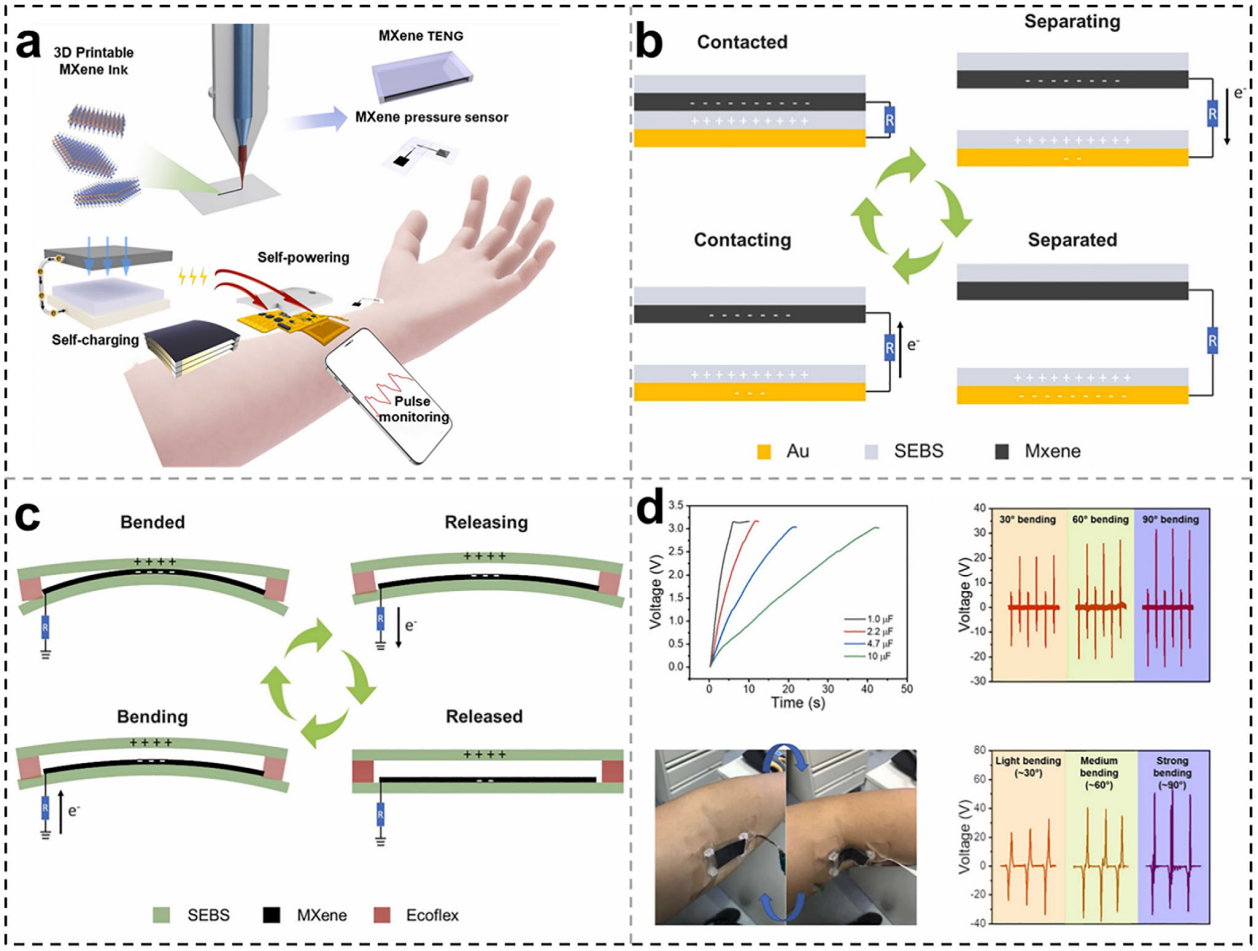
MXene‐based triboelectric nanogenerators (TENGs) for wearable and self‐powered health monitoring. (a) Schematic of a 3D‐printable Ti_3_C_2_T_*x*_ MXene functional ink used to fabricate flexible TENGs and pressure sensors integrated on a stretchable SEBS substrate. (b,c) Operating principle of the MXene TENG under contact‐separation and bending‐release modes, where alternating charge transfer between MXene, SEBS, and Ecoflex layers generates current. (d) Integrated self‐powered system combining TENG, rectifier, and capacitor circuits, demonstrating stable voltage generation. Continuous radial artery pulse (RAP) monitoring is achieved with high sensitivity (∼6.03. Reproduced with permission [162]. Copyright 2022, Elsevier.

As shown in Figure 20a–c, the printed MXene electrodes formed multilayered TENG architectures that efficiently converted biomechanical motion into electricity under both contact‐separation and bending‐release cycles. The integrated wearable platform (Figure 20d) went further, coupling the TENG to a rectifier‐capacitor circuit to provide continuous, battery‐free operation. Remarkably, the system demonstrated on‐demand radial artery pulse (RAP) waveform monitoring, powered entirely by harvested biomechanical energy. Its performance, power density of ∼816.6 mW/m^2^, sensitivity of ∼6.03 kPa^−^^1^, detection limit of ∼9 Pa, and rapid ∼80 ms response sets a benchmark for self‐powered physiological monitoring. Crucially, this example shows that functional ink design is the enabler of system‐level performance. Without the rheologically optimized MXene inks, printing would have been hampered by nozzle clogging, dispersion instability, or film cracking, undermining device reliability. Instead, the carefully engineered ink allowed MXene to act simultaneously as the electrode, sensing layer, and interconnect material bridging energy harvesting and wireless communication within one printable, skin‐compatible platform.

In addition to energy harvesting and sensing, MXene functional inks have proven vital in photonics, where their broadband optical absorption, ultrafast carrier dynamics, and solution processability make them ideal for printed saturable absorbers (SAs). By formulating stable MXene inks compatible with inkjet printing, uniform films with controllable thickness were deposited directly onto optical substrates (Figure 21a–c). This printability allows precise tuning of layer number and optical density, critical for tailoring nonlinear optical properties. The resulting MXene films exhibited excellent SA characteristics, including high modulation depth, low saturation intensity, and ultrafast recovery times, enabling both Q‐switching and mode‐locking operations across multiple wavelengths (Figure 21d,e). Notably, the printed MXene‐SAs were seamlessly integrated into fiber and solid‐state laser systems (Figure 21f,g), achieving stable ultrafast pulse generation, wide spectral coverage, and reproducibility over extended cycling.

**FIGURE 21:**
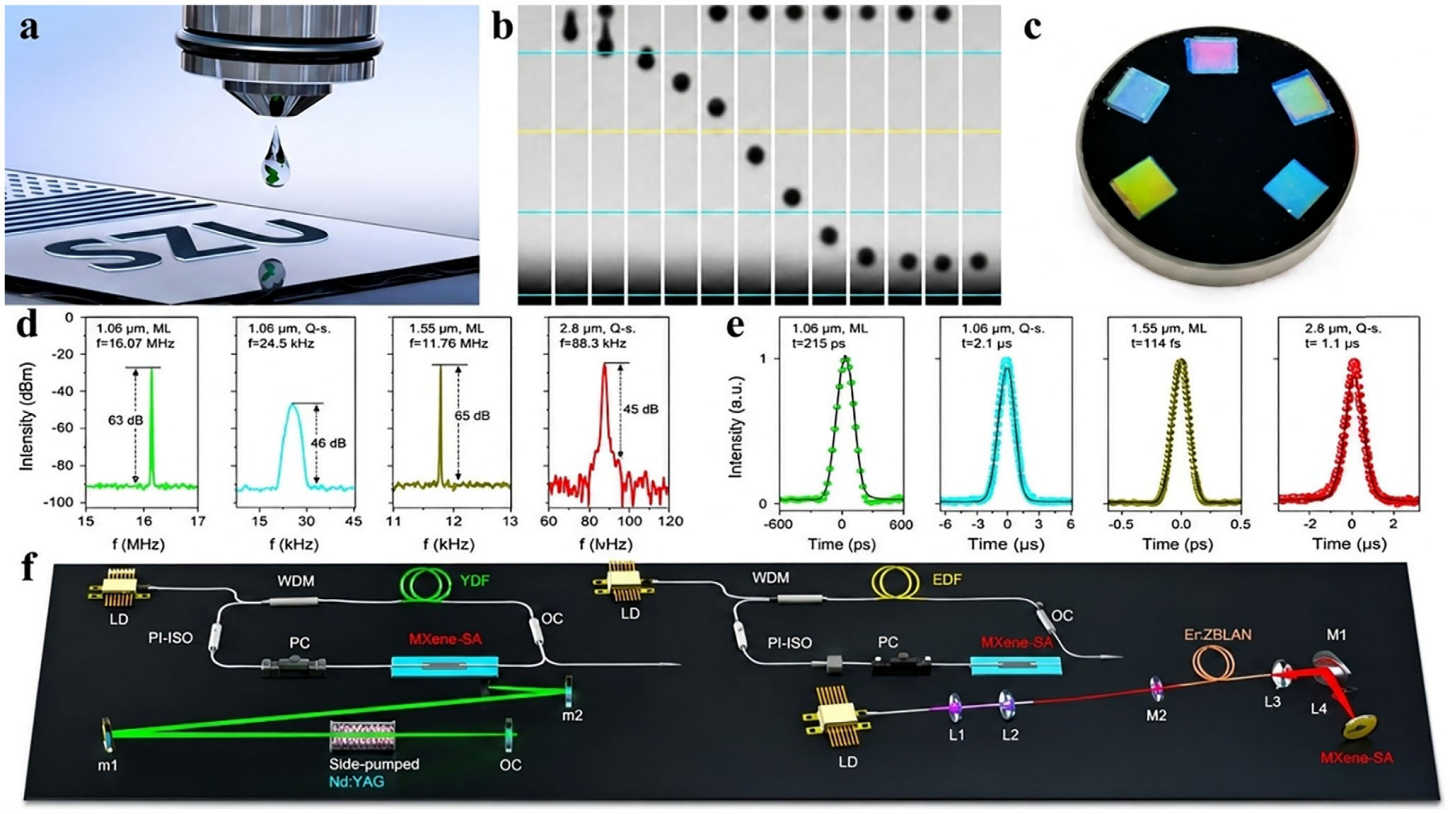
Inkjet‐printed MXene inks for ultrafast photonics. (a) Schematic of inkjet deposition of MXene nanosheets onto substrates for integration into laser resonators. (b) Optical micrographs of printed MXene droplet ejection sequence, demonstrating uniform spot size and pattern control. (c) Fabricated MXene films with tunable thickness (4–20 layers). (d) Frequency spectra of the MXene‐based saturable absorber (SA) under different laser modes, highlighting strong modulation depth and low noise. (e) Temporal pulse profiles showing ultrashort durations down to 100 fs. (f,g) Integration of printed MXene films into fiber and free‐space laser resonators, enabling broad spectral band ultrafast laser operations from near‐ to mid‐infrared regimes. Reproduced with permission [121]. Copyright 2019, Nature.

From a materials perspective, the success of these devices is directly linked to the functional ink design: dispersions with the right viscosity, flake size distribution, and stability ensured droplet uniformity during printing and prevented aggregation‐induced scattering losses. Unlike conventional vacuum‐deposited or mechanically transferred SAs, MXene inks provide a low‐cost, scalable, and customizable approach for integrating nonlinear optical elements into diverse photonic platforms. This study demonstrates how functional inks can bridge MXene's intrinsic optoelectronic properties with practical device architectures, positioning them as a promising class of printable photonic materials for ultrafast optics and optical communications.

In wireless and IoT electronics, the precision and reliability of printed antennas are often dictated by the quality of the functional inks. One study introduces a room‐temperature direct printing strategy for flexible wireless electronics, where additive‐free MXene inks are engineered for high precision and multifunctionality. The Ti_3_C_2_T_*x*_ aqueous inks were regulated to contain >90% single layers with narrow size distribution, ensuring stable flow, excellent jetting performance, and metallic‐level conductivity (∼6900 S/cm) in ultrafine‐printed tracks (Figure 22a,b). Importantly, these inks required no annealing or additives, simplifying processing and maintaining compatibility with heat‐sensitive substrates. The printed MXene antennas exhibited outstanding electronic performance, with Q factors rising and resistance decreasing with thickness (Figure 22d), alongside robust mechanical flexibility validated under strain (Figure 22e). Integration into functional modules demonstrated seamless NFC communication and wireless energy transfer, powering LEDs (Figure 22f) and enabling practical NFC‐tag applications for environmental and object sensing (Figure 22g,h). This work highlights how functional MXene inks, when properly stabilized and size‐controlled, can overcome long‐standing issues in printed wireless systems such as poor precision and conformality, unlocking scalable, room‐temperature fabrication of next‐generation IoT and wearable devices.

**FIGURE 22:**
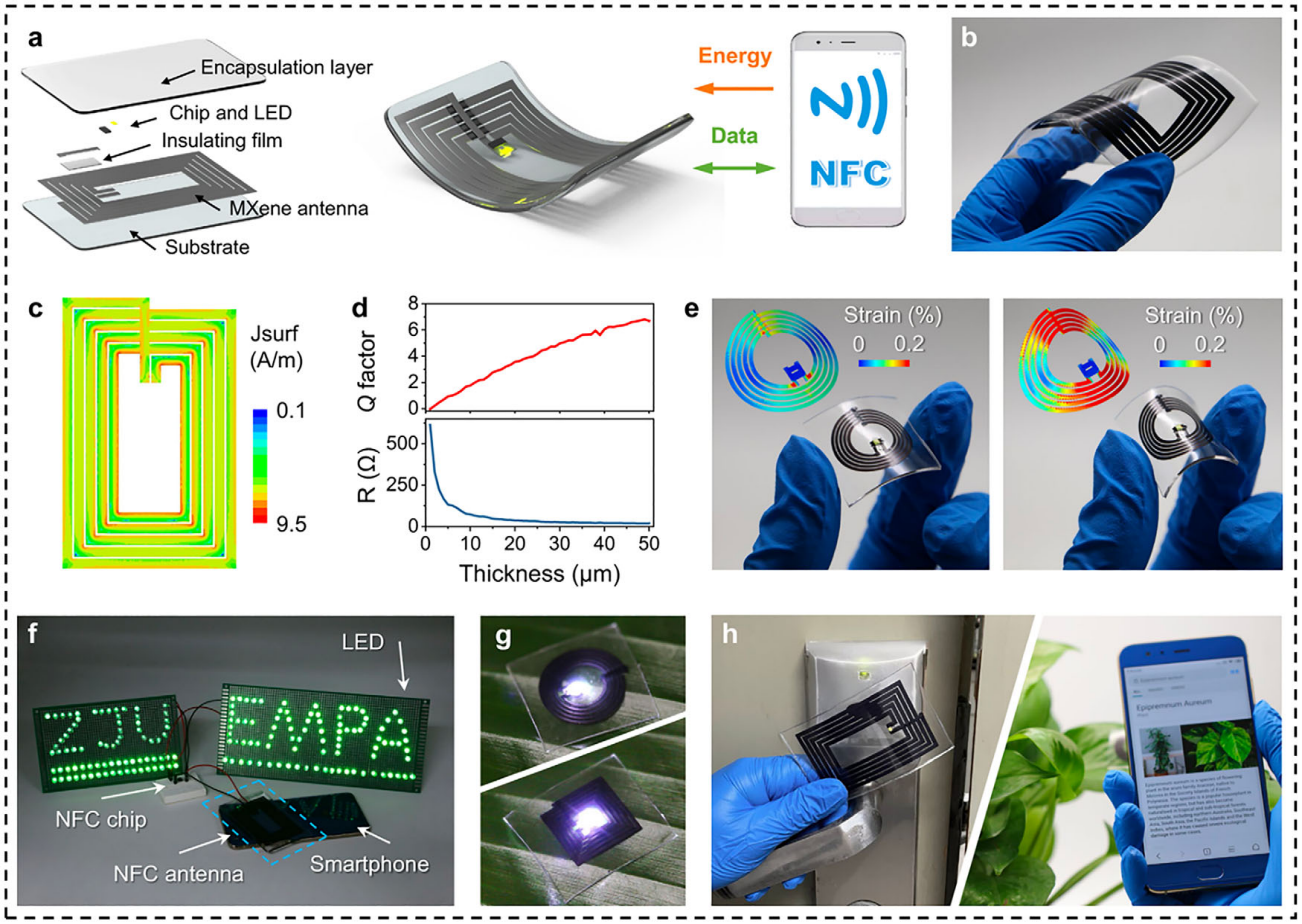
High‐resolution, room‐temperature printing of MXene NFC antennas and wireless electronics. (a) Schematic of the device architecture showing a printed MXene antenna integrated with a chip, LED, and encapsulation layers for energy and data transfer via NFC. (b) Photograph of a flexible, printed MXene NFC antenna. (c) Surface current density simulation validating efficient charge transport. (d) Q factor and resistance variation as a function of antenna thickness. (e) Mechanical strain tests confirm conformal flexibility without significant current disruption. (f) Demonstration of a printed MXene NFC circuit powering LEDs via wireless energy transfer from a smartphone. (g,h) Practical demonstrations of printed MXene NFC tags for smart sensing and wireless identification applications. Reproduced with permission [94]. Copyright 2022, Nature.

Scaling MXene inks into memory and logic devices requires inks that are not only conductive but also chemically stable, printable at high resolution, and environmentally robust. Here, alkylated‐DOPA‐functionalized MXene dispersions in ethanol provided the stability and rheological control necessary for EHD printing, which allows precise deposition with sub‐300 µm resolution and wafer‐scale uniformity (Figure 23a,b). Unlike conventional Au or Al electrodes, which require costly vacuum deposition, EHD‐printed MXene electrodes were entirely solution‐processed, enabling direct integration with ferroelectric PVDF‐HFP layers and ZTO semiconductors into one‐transistor‐one‐memory (1T1M) cells (Figure 23c,d).

**FIGURE 23:**
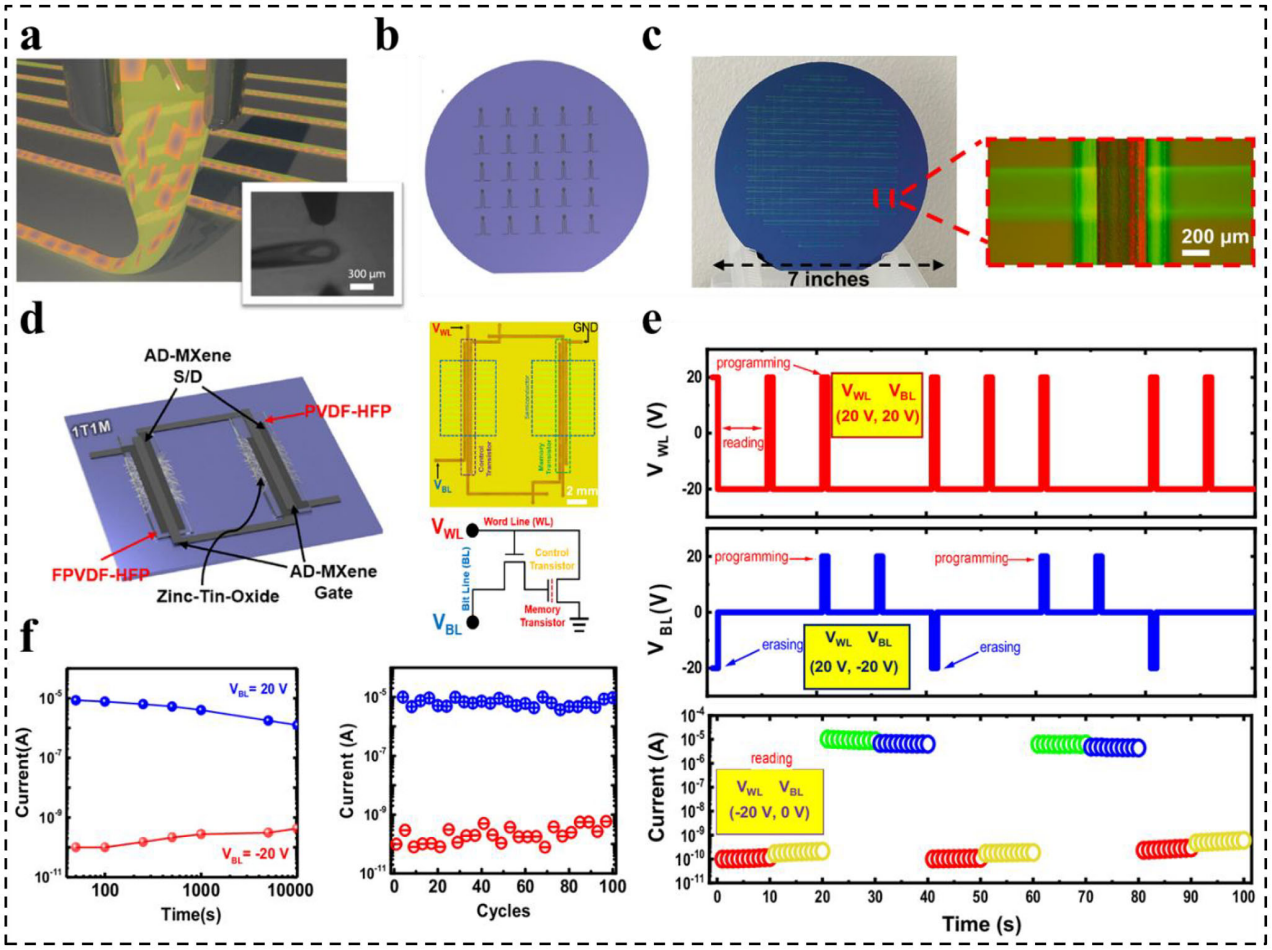
MXene‐based ferroelectric memory devices fabricated via wafer‐scale printing techniques. (a) Optical image of an EHD‐printed MXene thin‐film transistor array with finely aligned channels. (b) Wafer‐scale fabrication of MXene TFT arrays on silicon wafers, with a zoomed view showing electrode/channel structures. (c) Circuit schematic of the printed memory array architecture, with word line (WL), bit line (BL), and gate addressing for memory states. (d) Structure of a one‐transistor‐one‐memory (1T1M) cell, integrating alkylated‐DOPA (AD)‐functionalized MXene as source/drain and gate electrodes, a PVDF‐HFP ferroelectric layer, and a ZTO semiconductor channel. (e) Electrical operation under programming, erasing, and reading pulses demonstrates reliable logic state transitions. (f) Device performance tests confirm long retention, stable endurance across 100 switching cycles, and reproducibility of on/off states. Reproduced with permission [38]. Copyright 2024, Nature.

The electrical data (Figure 23e,f) confirmed that the printed MXene electrodes supported reliable programming, erasing, and reading operations, with long retention and stable cycling performance. Importantly, the hydrophobic functionalization of MXene reduced oxidation compared to pristine dispersions, ensuring device reliability over time. From a broader perspective, the adoption of EHD printing represents a critical advance: it bridges the gap between MXene's intrinsic properties and wafer‐scale device fabrication, combining low‐cost solution processing with the high precision typically associated with lithographic techniques. Compared to other printable conductors like PEDOT: PSS, MXene offers superior conductivity and breakdown strength, while avoiding the performance losses common in polymer‐based inks. This highlights how functional MXene inks, optimized for EHD printing, can be used in next‐generation microelectronics.

## 5 Challenges and Perspectives

1. For printed MXene technologies, the central scientific challenge is tuning material lifetime to the application. Many targets in energy storage, sensing, antennas, and interconnects require years of stable operation, whereas bio‐integrated or transient systems benefit from programmed degradation on much shorter timescales. MXenes, especially Ti_3_C_2_T_*x*_, are unusually sensitive to the environment and surface terminations, so lifetime engineering must couple surface functionalization (e.g., catechol shells, hydrophobic/Janus grafts), heterostructure barriers, interlayer pillaring, and defect control with device‐level encapsulation. In parallel, biocompatibility and long‐term toxicity must be established for different flake sizes/terminations and residual chemistries, and end‐of‐life plans for printed MXene devices should prioritize recyclability, solvent recovery, and safe transformation or capture of metal/halide species.
2. Maximizing device figures of merit still hinges on two MXene‐specific issues: restacking and charge/ion transport. After exfoliation, capillary drying and van der Waals forces drive layer re‐aggregation, shrinking accessible surface area and blocking ion percolation. Practical countermeasures include intercalants and spacers, porous/3D architectures via DIW + freeze‐drying, and shear‐assisted alignment that preserve pathways while keeping sheet resistance low. On the electronic side, printed performance depends on low contact resistance, continuous percolation, and controlled drying/annealing, which in turn require rheology‐by‐design: mapping viscosity, yield stress, *G*′/*G*″, surface tension, and solvent volatility to the demands of inkjet, EHD, screen, DIW, and aerosol‐jet processes. Additive‐free MXene inks enable metallic conductivity at room temperature, but typically need adhesion promoters or barrier stacks to ensure durability.
3. Competition and synergy with other ink families will shape adoption. Metal nanoparticle and liquid‐metal inks excel in conductivity but often need high‐temperature sintering or suffer oxidation; conductive polymers print easily yet have higher resistivity and RF loss; carbon inks (graphene/CNT) are robust and low‐cost but frequently require reduction/densification. MXenes sit between these, offering sinter‐free, high‐conductivity printing and the ability to form hybrid inks (with polymers, oxides, ionic media, or 2D semiconductors) that deliver application‐specific combinations of adhesion, elasticity, EMI absorption dominance, and ion buffering. The ability to mix dissimilar materials is a core advantage of the ink paradigm and will likely yield the most competitive MXene systems.
4. Commercialization introduces additional challenges. First, standardization is required because MXene batches with different termination ratios, impurity levels, lateral flake sizes, thickness distributions, oxidation states, and residual intercalants are often reported under the same material designation, which can undermine reproducibility and industrial confidence. Second, current MXene ink production involves several cost‐intensive steps, including MAX‐phase precursor synthesis, selective etching, repeated washing, delamination, stabilization, ink formulation, and quality control [101, 140]. In comparison, silver‐based inks benefit from mature supply chains and high electrical conductivity, but they involve high noble‐metal cost and often require post‐print sintering to achieve efficient particle–particle contact [185]. Carbon‐based inks, including graphene and carbon nanotube formulations, are generally more attractive in terms of material abundance, mechanical flexibility, and low‐temperature processability, but they often show lower conductivity than silver and may require higher loading, repeated printing, or post‐treatment [186]. MXene inks may offset part of their upstream production cost through low‐temperature, often sinter‐free processing and by combining electrical conductivity with electrochemical, sensing, or EMI‐shielding functionality. Accordingly, the “10× rule” should be interpreted as a practical adoption criterion rather than a universal economic threshold: MXene inks must offer a decisive advantage in performance, manufacturing simplicity, cost, or multifunctionality to compete with established conductive‐ink technologies.
5. Environmental footprint, circularity, and end‐of‐life recovery: The sustainability of printed MXene electronics depends on the full lifecycle, from MAX‐phase synthesis and etching to ink formulation, device operation, and disposal (Table 8). Conventional production often uses fluoride‐containing chemistry and repeated washing steps that generate acidic, metal‐containing waste and consume substantial water and energy. Greener approaches should therefore prioritize safer etchants, higher yields, solvent and water recovery, and continuous processing. Oxidation control is also important because longer ink shelf life and device lifetime reduce material loss and replacement demand. At end‐of‐life, design‐for‐disassembly, selective delamination, binder removal, and substrate reuse could facilitate recovery. Transition metals from Ti‐, V‐, Nb‐, and Mo‐based MXenes may be reclaimed through oxidation, leaching, precipitation, ion exchange, or solvent extraction, although their large‐scale feasibility remains to be established.
6. The technology‐readiness assignments shown in Figure 24 were developed using the nine‐level Technology Readiness Level framework established by NASA and standardized under ISO 16290. Each printed MXene technology was assigned the highest level for which sufficient evidence was identified in the published literature. TRL 3–4 was assigned to laboratory proof‐of‐concept and validated device demonstrations; TRL 5–6 required testing under application‐relevant conditions or demonstration of an integrated prototype; and TRL 7–9 was reserved for technologies demonstrated, qualified, or successfully operated in an intended operational environment. The scoring is based on the criteria set in Table 9. The assessment therefore reflects the maturity of specific printing‐process combinations rather than the general maturity of MXenes as a material class [187, 188].

**TABLE 8:** Environmental footprint, circularity, and end‐of‐life recovery of MXene.

Lifecycle stage	Main environmental concern	Potential mitigation strategy
MAX‐phase production	Energy‐intensive precursor synthesis	Lower‐temperature synthesis and improved precursor yield
Etching	HF or fluoride‐containing waste	Electrochemical, alkali, molten‐salt, or optimized low‐fluoride routes
Washing and delamination	High water and energy consumption	Closed‐loop water reuse and continuous separation
Functionalization and ink formulation	Organic solvents and additives	Safer solvents, solvent recovery, and minimal‐additive inks
Storage and printing	Oxidation‐related material loss	Passivation, controlled atmosphere, and encapsulation
Device end‐of‐life	Mixed‐material waste and difficult separation	Design‐for‐disassembly and selective layer delamination
Metal recovery	Dilute and mixed transition‐metal streams	Leaching, selective precipitation, ion exchange, and solvent extraction

**FIGURE 24:**
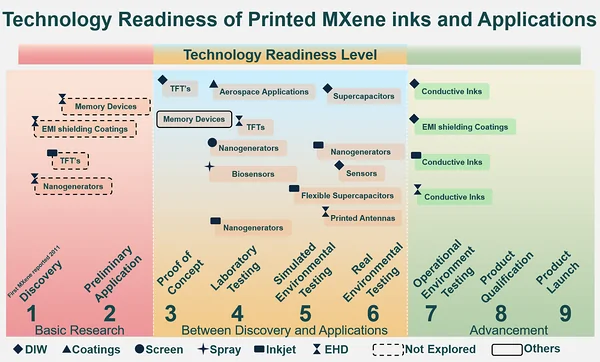
Technology readiness of printed MXene applications classified by printing method. TRLs were assigned by the authors using adapted NASA and ISO 16290 criteria and the highest level of evidence reported for each method‐application combination. Entries identified as research gaps indicate combinations for which no direct MXene‐printing study was found.

**TABLE 9:** The technology readiness level map scoring criteria.

TRL	Printed‐MXene‐specific interpretation	Minimum evidence expected
1	Basic principles observed	MXene material properties or mechanism discussed without device demonstration
2	Concept formulated	Device concept proposed; target function identified; printing route named but not validated
3	Proof of concept	First printed or coated device demonstration with basic functionality shown
4	Laboratory validation	Quantitative figure of merit reported on benchtop samples; at least basic process description available
5	Relevant‐environment validation	Device tested on relevant substrate or under application‐relevant conditions; repeat measurements reported
6	Prototype demonstration	Integrated prototype demonstrated with meaningful performance, repeatability, and preliminary environmental or cycling data
7	Operational prototype	System‐level demonstrator tested in realistic use case, field‐like environment, or integrated module
8	Qualified preproduction system	Manufacturing controls, specification windows, qualification testing, and near‐pilot integration established
9	Operational deployment	Technology proven in sustained real‐world use or commercial/industrial operation

Notably, the current TRL map for printed MXenes indicates an uneven but promising landscape (Figure 24): conductive inks/pastes, EMI‐shielding coatings, and supercapacitors are nearing deployment, while printed sensors, antennas, and nanogenerators are in mid‐TRL maturation and memory devices remain early‐stage [189, 190]. To accelerate progress, we see four near‐term priorities:

- Lifetime engineering through oxidation‐resistant chemistries and robust barrier stacks tailored to target lifetimes (years vs. weeks).
- Greener, continuous synthesis and in‐line delamination/washing to cut cost and variability.
- Rheology‐by‐design toolkits, ideally AI‐assisted, linking formulation → print window → feature size → device metrics; and
- Standardized metrology and qualification (printability indices, SE_T_/SE_R_/SE_A_ reporting, contact‐resistance protocols, bend/wash/humidity cycling).

7. Because printed electronics typically tolerate lower performance thresholds than CMOS/optoelectronics yet value cost, flexibility, and form factor, MXene inks are well positioned to fill a growing market gap. With coordinated advances in materials standardization, scalable green synthesis, data‐driven rheology, and end‐of‐life stewardship, printed MXenes can transition from compelling demonstrations to reliable, manufacturable platforms for flexible, wearable, and multifunctional devices.

## 6 Conclusion

MXene‐based functional inks have rapidly advanced into a transformative platform for next‐generation printable electronics, uniquely combining metallic conductivity, tunable surface chemistry, and solution processability with compatibility across diverse printing modalities. This review has underscored their successful deployment in sensors, energy storage devices, EMI shielding, wireless communication modules, and self‐powered systems, highlighting how ink formulation and printing fidelity directly govern device performance. Despite this progress, persistent challenges remain, particularly oxidative degradation in ambient environments, batch‐limited and hazardous synthesis routes, and the rheological complexities of tailoring inks for high‐resolution printing while preserving conductivity and adhesion. Encouragingly, emerging strategies including antioxidant stabilization, catechol‐based and Janus surface chemistries, continuous‐flow and green synthesis methods, and data‐driven rheological design are beginning to address these limitations. Coupled with advances in precision printing techniques such as EHD jetting and aerosol jet printing, MXene inks are increasingly demonstrating wafer‐scale, conformal, and multifunctional device integration. Looking ahead, interdisciplinary collaboration spanning materials chemistry, fluid mechanics, device engineering, and machine learning will be essential to transition MXene inks from laboratory‐scale demonstrations to reliable, industrial‐grade platforms. As the global demand for lightweight, flexible, and sustainable electronic systems continues to accelerate, MXene inks are poised to become a cornerstone material system, bridging nanoscale innovations with scalable manufacturing to enable the next generation of intelligent and multifunctional printed electronics.

## Abbreviations

**Materials & Chemistry**
ADOPA: alkylated 3,4‐dihydroxy‐*L‐*phenylalanine
CNF: cellulose nanofiber
CVD: chemical vapor deposition
CMC: carboxymethyl cellulose
CCMC: CaCl_2_‐crosslinked CMC
DA: dopamine
DOPA: 3,4‐dihydroxy‐*L*‐phenylalanine
FOTS: perfluorooctyl trichlorosilane
HF: hydrofluoric acid
J‐MXene: Janus MXene (asymmetrically grafted MXene)
LFP: lithium iron phosphate (LiFePO_4_)
LTO: lithium titanate (Li_4_Ti_5_O_12_)
MAX: M_n+1_AX_n_ phases (layered carbides/nitrides; A = group 13/14 element)
MBA: *N*, *N*′‐methylenebisacrylamide
MXene: 2D transition‐metal carbides/nitrides/carbonitrides (e.g., Ti_3_C_2_T*_x_*)
PANI: polyaniline
PCAT: pyrocatechol
PDMS: polydimethylsiloxane
DMAEMA: dimethylaminoethyl methacrylate
PEDOT: PSS: poly(3,4‐ethylenedioxythiophene): poly(styrenesulfonate)
PEI: polyethylenimine
PPy: polypyrrole
PVDF‐HFP: poly(vinylidene fluoride*‐co‐*hexafluoropropylene)
SEBS: styrene‐ethylene‐butylene‐styrene
T_*x*_
: surface terminations on MXene (─F, ─OH, ─O)

**Solvents, Electrolytes & Reagents**
DMF: *N*, *N*‐Dimethylformamide
DMSO: dimethyl sulfoxide
NMP: *N*‐Methyl‐2‐pyrrolidone
PC: propylene carbonate
TBAOH: tetrabutylammonium hydroxide

**Printing & Fabrication Methods**
DIW: direct ink writing (extrusion printing)
EHD: electrohydrodynamic (jet) printing

**Rheology & Dimensionless Groups**
Ca: capillary number
Oh: Ohnesorge number
Re: Reynolds number
We: Weber number
Z: inverse Ohnesorge number (*Z* = 1/Oh)

**Devices, Functions & Application Areas**
A‐MSC: asymmetric micro‐supercapacitor
EMI: electromagnetic interference
FET: field‐effect transistor
IoT: Internet of Things
LIMB: lithium‐ion microbattery
MSC: micro‐supercapacitor
MXetronics: MXene‐based electronics
NFC: near‐field communication
RF: radio frequency
TENG: triboelectric nanogenerator
TFT: thin‐film transistor

**Electromagnetic Metrics**
Q: quality factor
SE / SE_T_
: shielding effectiveness/total shielding effectiveness
SE_A_ / SE_R_
: shielding effectiveness by absorption/by reflection
S_11_
: input reflection coefficient (scattering parameter)

**Nanomaterials & Composites (Mentioned in Tables/Examples)**
AgNW: silver nanowire
PdNP: palladium nanoparticle
rGO: reduced graphene oxide

**Characterization & Test Conditions**
RH: relative humidity
SEM: scanning electron microscopy
TEM: transmission electron microscopy
UV–vis: ultraviolet–visible spectroscopy
X‐band: 8–12 GHz microwave band

## Author Contributions

H.A., D.B., and D.C. conceptualized and supervised the review; S.A. and F.S. prepared the initial and revised paper; H.H. and M.A. edited the revised draft; D.C. and F.S. revised the paper; H.A., D.B., and D.C. acquired funding for this work.

## Conflicts of Interest

The authors declare no conflicts of interest.

## Data Availability

The data that support the findings of this study are available from the corresponding author upon reasonable request.
